# Analysis of the Volatile Components in Different Parts of Three Species of the Genus *Amomum* via Combined HS–SPME–GC–TOF–MS and Multivariate Statistical Analysis

**DOI:** 10.3390/foods13121925

**Published:** 2024-06-18

**Authors:** Jingjing Gu, Meiquan Yang, Mingju Qi, Tianmei Yang, Li Wang, Weize Yang, Jinyu Zhang

**Affiliations:** 1 Medicinal Plants Research Institute, Yunnan Academy of Agricultural Sciences, Kunming 650200, China; jjgu2023@126.com (J.G.); lwang20@126.com (L.W.); yangweize116@163.com (W.Y.); 2 School of Agriculture, Yunnan University, Kunming 650504, China

**Keywords:** genus *Amomum*, volatile compounds, HS–SPME–GC–TOF–MS, differential metabolites

## Abstract

The study used headspace solid-phase microextraction coupled with gas chromatography-time-of-flight mass spectrometry (HS–SPME–GC–TOF–MS) to analyze volatile compounds in leaves and fruits of *Amomum tsaoko*, *Amomum paratsaoko*, and *Amomum koenigii*. The composition and aroma of distinct metabolites were analyzed using multivariate statistical methods. A total of 564 volatile compounds were identified from three species of the genus *Amomum*, which were further divided into nine categories: terpenoids, carboxylic acids, alcohols, hydrocarbons, aldehydes, ketones, phenols, ethers, and other compounds. Terpenoids and alcohols were the most abundant. The content and types of compounds vary in *A. tsaoko*, *A. paratsaoko*, and *A. koenigii*, so mixing or substituting them is not advisable. We selected 45 metabolites based on the criteria of the variable importance in projection values (VIP > 1.5) and one-way ANOVA (*p* < 0.05). The top 19 metabolites with the most significant VIP values were chosen. Interestingly, (*Z*)-2-decenal was only found in *Amomum koenigii*, while nitroethane and nonanal were only present in cultivated *A. tsaoko*. Additionally, linalool, cineole, and (*D*)-limonene were the main components affecting the aroma of three species of the genus *Amomum*. The volatile components identified in this study provide a theoretical basis for analyzing the unique flavor of *A. tsaoko*, *A. paratsaoko,* and *A. koenigii*.

## 1. Introduction

The genus *Amomum* belongs to the family Zingiberaceae and includes over 150 species of perennial herbaceous plants. These plants are mainly found in tropical regions of Asia and Oceania, with around 24 species in southern China [1]. Most genus *Amomum* plants possess distinctive aromas and are rich in phytochemicals, making them traditional ethnic plants. The fruits of these plants are rich in volatile oils, including flavonoids, terpenoids, diarylheptanoids, coumarins, and other compounds [2]. These compounds have pharmacological activities such as antioxidant [3,4], anti-inflammatory [5,6], anti-allergic [7,8,9], and antibiotic properties [10]. Throughout history, most of the plants of this genus have been used as medicinal herbs or spices.

*Amomum tsaoko* is concentrated in Yunnan, China. *The Pharmacopoeia of the People’s Republic of China* records that dry and mature fruits used as medicine contain a unique aroma with a pungent and slightly bitter taste [11]. Therefore, its fruit is often used as spices and medicinal materials and was included in the list of medicinal and food sources by the National Health Commission in 2014. Although many studies have reported volatile compounds extracted from fruits (more than 209 types), including terpenoids, alcohols, organic acids, and phenols [12], there is currently little or no understanding of the compounds present in the fruits and leaves of *A. tsaoko, A. paratsaoko*, and *A. koenigii*. Investigation and research have found that *A. tsaoko* is challenging to distinguish due to its similarity in morphology to plants of the same genus [13]. In the market, *Amomum paratsaoko* and *Amomum koenigii* are often used as substitutes for *A. tsaoko*, leading to a lack of clinical efficacy and medication safety guarantee. In addition, different species within plants of the genus *Amomum* have different odors, such as the intensely pungent aroma of *A. tsaoko* and the fresh aroma of *A. paratsaoko*. However, the odor variation in genus *Amomum* plants has yet to be systematically studied. Studying this phenomenon helped us better understand the unique aroma of *Amomum* plants.

“Plant odor” describes the fragrance given off by volatile organic compounds produced through secondary metabolism in a plant. Due to their low molecular weight, low boiling point, and low polarity, these compounds pose a challenge when quantifying and analyzing plant volatile odors [14]. Based on the aforementioned characteristics, it is worth noting that traditional extraction methods like steam distillation, organic solvent extraction, and supercritical CO_2_ fluid extraction may potentially harm plant samples. Furthermore, factors such as temperature fluctuations, duration of exposure, and other variables can introduce contamination to volatile substances, which consequently impacts their authenticity [15,16]. Moreover, different extraction methods yield different volatile compounds, even within the same species [17]. Further research has shown that volatile compounds may differ significantly between species and organs within the same genus [18,19].

In recent years, headspace solid-phase microextraction (HS–SPME) has been the primary method for collecting volatile substances, with the advantages of simple, fast, and sensitive operation [20]. In addition to being widely used to collect plant odor [21], such as *hemerocallis fulva* [22], and green tea [23], it is also often used to collect volatile substances in *Ficus carica* L. [24] and flavor substances in Baijiu [25], etc. Compared with solid-phase microextraction methods (SPME), HS–SPME is widely used for separating and preconcentrating volatile compounds before gas chromatography-time-of-flight mass spectrometry (GC–TOF–MS) analysis, using headspace air injection to avoid reagent contamination caused by desorption using organic solvents.

This study used headspace solid-phase microextraction coupled with gas chromatography-time-of-flight mass spectrometry (HS–SPME–GC–TOF–MS) to identify the flavor compounds of three species in the genus *Amomum*. Considering the convenience of carrying the adsorbent, which is conducive to the capture and enrichment of volatile organic compounds (VOCs) in the sample and is more accurate for subsequent analysis, an adsorbent was selected to gather VOCs from plants.

The study aimed to analyze the relative metabolite content of VOCs present in three species of genus *Amomum* plants in their natural state. The objective was to provide a theoretical basis for analyzing the aroma components of genus *Amomum* plants and contribute to understanding the aroma system of ethnic plants.

## 2. Materials and Methods

### 2.1. Materials

To minimize the differences in chemical composition caused by the plants themselves, *Amomum tsaoko*, *Amomum paratsaoko*, and *Amomum koenigii* were all collected from mature and fragrant fruits, mostly obovate and oval-shaped, with mature leaves of bright green color, with a smooth surface, and free from diseases, pests, and damage. The leaves of *A. tsaoko* and *A. paratsaoko* were oval shaped, while *A. koenigii* leaves were lanceolate (Figure S1). These three plants all prefer warm and humid climates, mostly growing in sparse forests and shaded conditions. In this study, 18 fresh samples from 3 species were dried to a constant weight using an electric hot air drying oven (model DHG–9240, Shanghai Yiheng Scientific Instrument Co., Ltd., Shanghai, China) at 50 °C and stored in a sealed bag at room temperature. Therefore, the samples were all identified by researcher Dr. Jinyu Zhang (Institute of Medicinal Plants, Yunnan Academy of Agricultural Sciences, Kunming, China).

In the period of September to October 2022, a technique called headspace solid-phase microextraction was utilized to extract volatile components from the fruits and leaves of *A. tsaoko*, *A. paratsaoko*, and *A. koenigii*, which included leaves and fruits. An amount of 0.5 g of each sample was added to a 20 mL headspace bottle; the bottle was then sealed and capped. Each sample in the study was a mixture of 12 leaves or 18 fruits from three different plants. There were three biological replicates in each sample. The sample extraction method utilized in the study was solid-phase microextraction (SPME) separation technology. The CTC three-in-one automatic sampler was equipped with a 50/30 μmDVB/CARonPDMS extraction head. The sample was shaken at 250 rpm for 15 min at 50 °C, and the extraction time was 30 min. The resolution time was 5 min, and the GC cycle time was 50 min for sample processing [26].

### 2.2. CG–MS Analysis

This study analyzed the extract using the American Agilent 5977B gas chromatography-mass spectrometer. The chromatography column was DB-wax (30 m × 0.25 mm × 0.25 µm). The injection temperature was set to 260 °C, and the split ratio was not split. The carrier gas was helium, with a purity of 99.999% and 1 mL/min flow rate. The initial column temperature was 40 °C, maintained for 5 min, increased to 220 °C at 5 °C/min, then increased to 250 °C at 20 °C/min, and maintained for 2.5 min. The interface temperature was set to 260 °C. The ion source temperature was 230 °C, and the fourth pole was 150 °C. The ionization method was EI+, 70 ev. The scanning method was full scan, and the quality range was 20–400 [27].

The relative content of each metabolite was calculated using the concentration and peak area of the internal standard. Qualitative analysis was conducted by comparing the compound fragment information collected by mass spectrometry with the standard sample information in the NIST2014 spectral library. Identification was carried out by comparing the mass to charge ratio, retention time, relevant literature, and other reference information of ion fragments, and the CG–MS results were evaluated.

### 2.3. Data Analysis

The data were preprocessed through square root transformation and Pareto scaling before modeling. MetaboAnalyst https://www.metaboanalyst.ca (accessed on 3 December 2023) was used to conduct the orthogonal projection of principal component analysis (PCA) and partial least squares discriminant analysis (PLS–DA) to obtain an overview of the entire data. PCA was used to summarize the data and detect outliers, while PLS–DA was used to maximize the separation between comparison groups. To ensure that the PLS–DA results were not overfitting, a test was conducted 1000 times. Permutation testing was used as an external validation method to analyze the model for overfitting and determine its reliability [28]. Finally, a heat map of volatile compounds was drawn to analyze the clustering situation.

Differential compounds were screened using variable importance in projection (VIP) thresholds and *p* values to identify differences in compounds across different regions. The correlation between plant and chemical components was analyzed using partial least squares (PLS). Single-factor tests were used to analyze the exact part (leaves or fruits), while independent sample T–tests were used to compare the compound content of different parts (leaves and fruits) for the same species. The statistical analyses were performed using SPSS 27.0.1, and a *p*-value < 0.05 was considered statistically significant.

## 3. Results and Discussion

### 3.1. Identification and Quantitation of Volatiles from Three Genus Amomum Species by GC–MS

**Figure 1:**
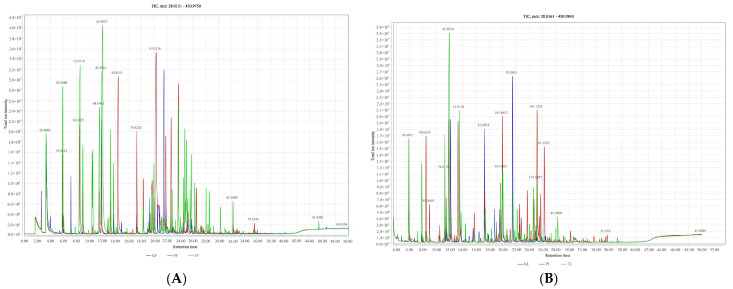
Total ion Chromatogram (TIC) of fruits and leaves of three species of the genus *Amomum* ((**A**) fruit; (**B**) leaves; T: *A. tsaoko*; P: *A. paratsaoko*; K: *A. koenigii*).

The leaves and fruits of *A. tsaoko*, *A. paratsaoko*, and *A. koenigii*, were abundant in metabolites. The results of the metabolite database, mass spectrometry fragment data, and literature search are shown in Table S1. A total of five hundred sixty-four volatile compounds were detected, including one hundred fifty-two terpenoids, one hundred thirty-nine carboxylic acids, sixty-five alcohols, fifty-two hydrocarbons, fifty-one aldehydes, thirty-six ketones, twelve phenols, nine ethers, and forty-seven other compounds. The total ion chromatograms (TICs) of these compounds are shown in Figure 1, where different colored lines represent different species

**Figure 2:**
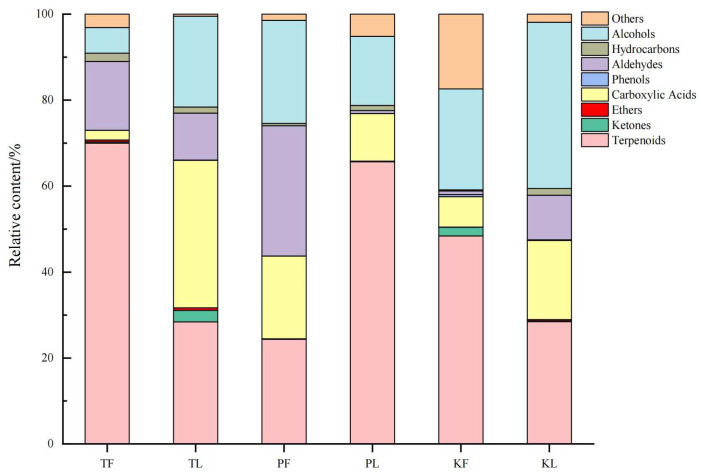
The relative contents (%) of different volatile compounds in three species of *Amomum* (T: *A. tsaoko*; P: *A. paratsaoko*; K: *A. koenigii*; L: Leaf; F: Fruit).

The content and types of terpenoid compounds in the leaves and fruits of these three species are the highest (Figure 2). The variation trend of compound content aligns with the relative composition (%). Hydrocarbons and ethers are typically colorless and odorless, contributing minimally to the flavor. There are differences in the compounds of *A. tsaoko*, *A. paratsaoko*, and *A. koenigii* among different parts and within the same species. Volatile metabolites in TF are mainly terpenoids (70%), followed by aldehydes (16%); volatile metabolites in TL are mainly carboxylic acids (34%), followed by terpenoids (28%) and alcohols (21%); volatile metabolites in PF are mainly terpenoids (24%), carboxylic acids (19%), aldehydes (30%), and alcohols (24%), with a relatively uniform distribution of relative content. Volatile metabolites in PL are mainly terpenoids (66%), followed by alcohols (16%); volatile metabolites in KF are mainly terpenoids (48%), followed by alcohols (23%); volatile metabolites in KL are mainly terpenoids (28%) and alcohols (39%), followed by carboxylic acids (18%).

### 3.2. Comparative Analysis of Volatile Metabolites in Different Parts of Three Species of the Genus Amomum

**Figure 3:**
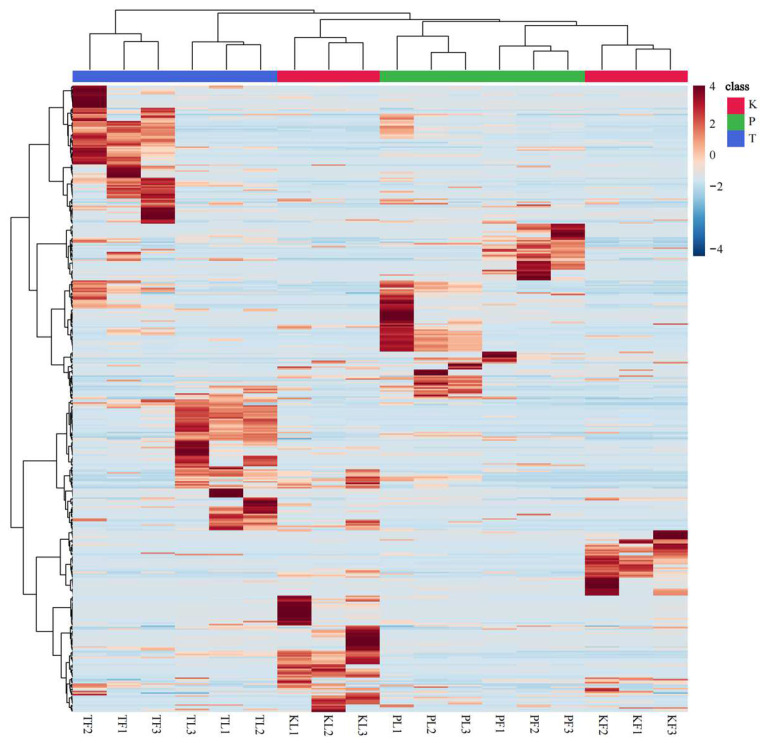
Heatmap of GC–MS determination of volatile components of three species of the genus *Amomum* from different parts. (The color of the heat map represents the relative content of the metabolites. High and low expression levels of metabolites are shown in red and blue, respectively).

A heat map of volatile compounds in the leaves and fruits of *A. tsaoko*, *A. paratsaoko*, and *A. koenigii* plants was created using MetaboAnalyst better to understand their content differences (Figure 3). The Euclidean clustering results indicated variations in the volatile metabolites in different parts of the same species and different parts of different species, which is consistent with the previous analysis that there are significant differences in volatile compounds between *A. tsaoko* and *A. paratsaoko* [29].

In the fruits, cineole has therapeutic effects on respiratory diseases, cardiovascular diseases, and digestive system diseases [30], was the metabolite with the highest relative content in *A. tsaoko*. Comparatively, (*Z*)-2-decenal, a fatty aldehyde with antibacterial and insecticidal effects [31], was the metabolite with the highest relative content in *A. paratsaoko*. Linalool, a common natural monoterpene alcohol that exists widely in many aromatic plants and has various biological activities, such as anti-cancer, antibacterial, neuroprotective, anti-anxiety, anti-depression, anti-stress, liver protection, kidney protection, and lung protection activities [32], was the metabolite with the highest relative content in *A. koenigii*. For the leaves, the metabolite with the highest relative content in *A. tsaoko* leaves was consistent with that in *A. tsaoko* fruits. The metabolite with the highest relative content in *A. paratsaoko* was (-)-limonene, which has antibacterial, anti-cancer, anti-inflammatory, and insecticidal activities [33]. Meanwhile, 2-hexenal, the metabolite with the highest relative content in *A. koenigii*, is found in various fruits, vegetables, and spices, and has the function of seasoning and antibacterial activities [34] (Tables S2 and S3). These findings indicate that there are significant differences in the types and relative contents of volatile metabolites between different species or different parts of the same species, and the pharmacological effects of these major volatile metabolites are inconsistent. Therefore, it is not recommended to use them interchangeably. It is worth noting that non-medicinal parts (such as leaves) produce a large amount of waste during the growth and harvest of plants. If they are not properly utilized, they will not only cause environmental pollution but also waste valuable biological resources. However, existing studies have confirmed that these non-medicinal parts are rich in bioactive ingredients and are cheap, readily available, and extremely valuable resources. Therefore, strengthening the research and utilization of these non-medicinal parts is of great significance for maximizing resource utilization and environmental protection.

In a study that analyzed the chemical makeup of different species within the same genus, researchers found significant differences in the volatile metabolites of each species. Moreover, there were variations in the types and contents of the volatile metabolites even within different parts of the same species. While the types of volatile metabolites were similar within the fruits of the same species, their concentrations differed. Apart from species differences, environmental factors can also affect the composition of volatile metabolites. This conjecture is supported by research that examined the effects of different environments on the volatile oil of *A. tsaoko* [35]. The geographic environment can also impact plant flavors [36]. The chemical composition of plants becomes more similar when they grow in geographically closer environments [37].

### 3.3. PCA Analysis of Volatile Components

**Figure 4:**
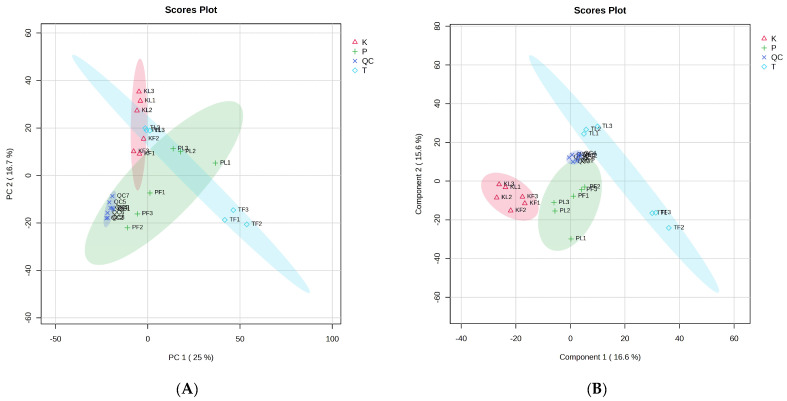
PCA score plot and PLS–DA score plot of three species of the genus *Amomum*. ((**A**) PCA score plot of *A. tsao-ko*, *A. paratsaoko*, and *A. koenigii* from different parts; (**B**) PLS–DA score plot of *A. tsao-ko*, *A. paratsaoko*, and *A. koenigii* from different parts).

PCA is a method for data dimensionality reduction, which recombines the original variables into several new unrelated variables to extract and reflect critical information about the actual variables [38]. In this study, PCA was performed on the GC–MS results of *A. tsaoko* and adulterant plant leaves and fruits in order to differentiate the differences between samples from different species. PC1 and PC2 accounted for 25% and 16.7% of the total variance, respectively (Figure 4A). In PC1, *A. tsaoko* and adulterant plant samples showed clear differentiation along the PC1 axis. The samples from different species could be effectively distinguished, which was consistent with the results of the clustering analysis and enabled precise classification based on species. Since PCA is an unsupervised model validation method, it cannot eliminate intra-group noise. Therefore, PLS–DA was conducted to address this issue.

### 3.4. PLS–DA Analysis of Volatile Components

Compared to PCA, PLS–DA is a supervised pattern recognition method used to describe the separation trend between different parts of *A. tsaoko* and adulterant plants. The PLS-DA score plot showed that the three groups of samples were distributed on both sides of PC1, and PC1 and PC2 explained 16.6% and 15.6% of the total variance, respectively (Figure 4B). The leading component regression coefficient Q^2^ of the model was 0.798 > 0.5, indicating the model’s predictive solid ability. At the same time, R^2^ was 0.971, indicating that the model is an excellent fit. Considering that the supervised classification model is prone to overfitting, 1000 permutation tests were conducted to verify its accuracy (Figure S2); the results indicate that the model did not overfit and the evaluation model is reliable and effective. Therefore, it can be used to analyze the volatile components of different genus *Amomum* plants and for metabolite screening.

### 3.5. Analysis of Characteristic Compounds

Multiple statistical analysis was used to screen key biomarkers of volatile compounds in different plant parts. Therefore, we screened 45 odor active compounds from the samples of the genus *Amomum* utilizing the analysis of variance statistical method (*p* < 0.05) and the projection variable importance value (VIP > 1.5). The top 19 most significant differential metabolites: linalool, (*Z*)-2-decenal, nitroethane, (*E*)-2-octenal, cineole, (*E*)-,4,8-dimethyl-1,3,7-nonatriene, .gamma.-muurolene, 2-hexenal, copaene, m-cymene, methyl caproate, neral, (*D*)-limonene, l-pinocarveol, cis-linalool oxide, nonanal, myrtenal, alloaromadendrene, and (*E*)-dec-2-enyl acetate were selected to distinguish between *A. tsaoko*, *A. paratsaoko*, and *A. koenigii*.

The study analyzed 19 components in samples of three species of the genus *Amomum* (fruits and leaves), and the results are shown in Table 1. Among these components, (*Z*)-2-decenal is unique to *A. koenigii*, while nitroethane and nonnal are unique to *A. tsaoko*. Meanwhile, linalool, (*E*)-2-octanal, cineole, (*E*)-,4,8-dimethyl-1,3,7-nonatriene, copaene, and (*D*)-limonene were found in the samples of these three species. In addition, 2-hexenal was found only in leaves, but not in fruits; neral was found to be present in both the TF and KF. The rang of cineole in the fruit of *A. tsaoko* is the highest, which is significantly higher than that of the other two species, and the content in the leaves of *A. paratsaoko* is also higher than that of the other two species. In general, there are significant differences in the rang of compounds in samples from different species and parts. However, linalool, cineole, and (*D*)-limonene are the most characteristic components. Their content in different species and parts is higher than that of other components.

The differences in the types and contents of volatile components in fruits and adulterants are the reasons for the unique aroma of each species. In fact, their unique aroma is not the result of the action of a single volatile component but rather the impact of the action of multiple volatile components. Cineole has a camphor-like odor and an excellent spicy taste similar to mint [39]. It is also used as a flavor and aroma enhancer in food, cleaning products, and cosmetic formulations [40]. Furthermore, cineole is also commonly found in *eucalyptus* [41], *mint* [42], and some *citrus* [43]; the content varies among different species.

(*D*)-limonene is a colorless transparent liquid with a pleasant citrus odor, which is widely used in cosmetics, food, and spices. (*D*)-limonene is a spice and insecticide that exists in the peel of various citrus plants. It is the primary aroma component of citrus, and its content is usually between 70% and 48%, which helps to produce its fruit flavor [43,44]. In addition, *A. tsaoko*, *A. paratsaoko*, and *A. koenigii* contain a large number of alcohols and aldehydes, which have a strong odor. Five aldehydes were screened out: (*Z*)-2-decanal, (*E*)-2-octenal, 2-hexenal, neral, nonanal. (*E*)-2-octenal has the characteristic odor of green, nut, and fat. Previous studies have shown that this compound is a promising artificial mixture of nematicides. Neral only exists in TF, and it has a lemon flavor. It is roughly the same as that of aldehydes confirmed in previous studies [44]. Aldehydes contain a carbonyl group (C=O), and the carbonyl group has strong polarity. This polarity can cause the oxygen atoms of aldehyde molecules to attract the surrounding hydrogen atoms and form hydrogen bonds. Hydrogen bonds make aldehyde molecules more closely bound to olfactory receptors, resulting in a strong pungent odor, which is conducive to forming a unique flavor with *A. tsaoko*, *A. paratsaoko*, and *A. koenigii*.

**Table 1:** Significance analysis of the average relative content of 20 substances with high VIP scores.

Compound Name	Class	Average Relative Content/%						*p*-Value	VIP	Odor Description	Reference
		TF	TL	PF	PL	KF	KL				
Cineole	Terpenoids	16.5 ± 3.64 Aa	5.62 ± 3.24 Ab	1.68 ± 0.39 Ba	5.03 ± 4.2 Aa	3.72 ± 0.89 Ba	0.1 ± 03 Aa	0.000	3.13	Pungent, Cooling, Spicy	[45]
(*E*)-,4,8-Dimethyl-1,3,7-nonatriene		0.02 ± 0 Ba	0 ± 0 Ba	0 ± 0 Ba	0.01 ± 0 Ba	0.59 ± 0.34 Aa	0.35 ± 0.11 Aa	0.000	3	-	-
.gamma.-Muurolene		3.61 ± 1.2 Aa	0.06 ± 0.05 Bb	0.08 ± 0.01 Bb	0.97 ± 0.44 Ab	0 ± 0 Ba	0.02 ± 0.01 Bb	0.016	2.79	-	-
Copaene		2.36 ± 0.88 Aa	0.95 ± 0.37 Ba	0.53 ± 0.2 Ba	3.49 ± 1.59 Aa	0.01 ± 0 Ba	0.02 ± 0 Ba	0.000	2.36	-	-
m-Cymene		1.05 ± 0.8 Aa	0.51 ± 0.08 Aa	0 ± 0 Ba	0.25 ± 0.22 ABa	0 ± 0 Ba	0.14 ± 0.12 Ba	0.000	2.26	-	-
(*D*)-Limonene		16.5 ± 5.64 Aa	5.62 ± 3.24 Ab	1.68 ± 0.39 Ba	5.03 ± 4.2 Aa	3.72 ± 0.89 Ba	0.1 ± 0.03 Ab	0.017	2.22	Fresh, Citrus	[46]
Myrtenal		0.27 ± 0.12 Aa	0.06 ± 0.01 Aa	0.01 ± 0 Ba	0 ± 0 Aa	0 ± 0 Ba	0 ± 0 Aa	0.000	2.01	Refreshing, Spicy-herbaceous odor	[47]
Alloaromadendrene		0.05 ± 0.03 Aa	0 ± 0 Aa	0 ± 0 Ba	0.09 ± 0.01 Aa	0 ± 0 Ba	0 ± 0 Aa	0.002	2.19	-	-
(*Z*)-2-Decenal	Aldehydes	4.41 ± 3.7 Aa	0 ± 0 Aa	1.42 ± 1.16 Aa	0 ± 0 Aa	0 ± 0 Aa	0 ± 0 Aa	0.013	3.64	-	-
Neral		0.14 ± 0.05 Aa	0.24 ± 0.14 Aa	0 ± 0 Aa	0 ± 0 Aa	0.14 ± 0.08 Aa	0 ± 0 Aa	0.034	2.34	Lemon-like	[44]
(*E*)-2-Octenal		1.46 ± 1.16 Aa	0.17 ± 0.09 Aa	0.72 ± 0.53 Aa	0.03 ± 0.02 Ba	0.01 ± 0 Aa	0.01 ± 0 Aa	0.008	3.07		
2-Hexenal		0 ± 0 Ba	0.16 ± 0.01 Bb	0 ± 0 Aa	0.06 ± 0.04 Ba	0 ± 0 Ba	3.54 ± 1.6 Aa	0.019	2.62	Fatty, Green	[34]
Nonanal		0.14 ± 0.05 Aa	0.24 ± 0.14 Aa	0.24 ± 0.14 Aa	0 ± 0 Aa	0 ± 0 Ba	0 ± 0 Aa	0.002	2.19	Orange-rose odor Floral, Waxy, Green	[48]
I-Pinocarveol	Alcohols	0.24 ± 0.15 Aa	0.37 ± 0.09 Aa	0.02 ± 0.01 Aa	0 ± 0 Ba	0 ± 0 Aa	0.01 ± 0.1 Aa	0.000	2.25	-	-
cis-Linalool Oxide		1.42 ± 0.2 Aa	0.15 ± 0.07 Aa	0.29 ± 0.05 Ba	0 ± 0 Bb	0.34 ± 0.18 Ba	0.09 ± 0.2 Ba	0.002	2.19	-	-
Methyl caproate	Esters	0.03 ± 0.02 ABa	0 ± 0 Ca	0.06 ± 0.03 Aa	1.01 ± 0.22 Ab	0 ± 0 Ca	0.5 ± 0.21 Ba	0.004	2.29	Pineapple, Ethereal	[49]
(*E*)-Dec-2-enyl acetate		0.06 ± 0.05 Aa	0.06 ± 0.02 Aa	0.09 ± 0.05 Aa	0 ± 0 Ba	0 ± 0 Aa	0 ± 0 Ba	0.002	2.01	-	-
Nitroethane	Other	0 ± 0 Aa	0 ± 0 Aa	0 ± 0 Aa	0 ± 0 Aa	4.65 ± 4.03 Aa	0 ± 0 Aa	0.019	3.23	Mild, Fruity	-

Note: different capital letters indicate significant differences in the same part of different species at the 0.05 level. In comparison, different lowercase letters indicate significant differences in other parts of the same species at the 0.05 level.

### 3.6. Correlation between Plant Parts and Chemical Components

**Figure 5:**
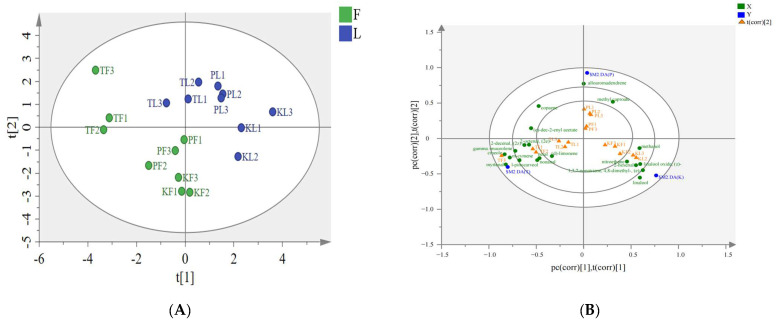
PLS–DA loading plots derived from 20 volatile compounds of different parts ((**A**) PLS–DA score chart of *A. tsaoko*, *A. paratsaoko*, and *A. koenigii* from different parts; (**B**) PLS–DA biplot chart of *A. tsaoko*, *A. paratsaoko*, and *A. koenigii* from different parts).

Partial least squares (PLS) regression analysis is used to evaluate the correlation between three species of genus *Amomum* plants and 20 selected chemical components. The mass intensities of compounds and related plant parts are set as X and Y variables, respectively. The leaves and fruits of plants are divided into two groups along the Y-axis (Figure 5A), while the TF is distinguished from the two species of KF and PF, which is consistent with the results in Figure 3.

Most of the 19 compounds screened were highly correlated with TF and TL; cineole, neral, (*Z*)-2-decenal, and (*E*)-2-octenal have a higher correlation, so these substances may be an essential source of the unique odor of *A. tsao-ko*. In the relevant research reports of *A. tsaoko*, terpenes are the main component, and eucalyptus oil is the most critical component [12]. Although terpenoids may have a significant effect on the specific odor of *A. tsao-ko*, their mechanism of action remains to be further studied. KL and KF contain more alcohols, aldehydes, and esters.

## 4. Conclusions

This study had employed the techniques of headspace solid-phase microextraction (HS–SPME) and gas chromatography-time-of-flight mass spectrometry (GC–TOF–MS), along with multivariate statistical analysis, to analyze the fruits and leaves of *A. tsaoko*, *A. paratsaoko,* and *A. koenigii*. We identified 564 metabolites in the fruits and leaves, mostly comprising terpenes and alcohols. Further calculations of the relative content of fruits and leaves from the three *Amomum* species revealed that cineole, (*Z*)-2-decenal, and linalool were the volatile metabolites with the highest relative content in the fruits of *A. tsaoko*, *A. paratsaoko,* and *A. koenigii*, respectively; while cineole, (-)-limonene, and 2-hexenal were the highest in their leaves. This indicates significant differences in the types and relative contents of volatile metabolites among different species and within the same species in different parts. Given the diverse pharmacological effects of these primary volatile metabolites, it is not recommended to use them interchangeably. Additionally, non-medicinal parts (leaves) are rich in bioactive compounds; thus, research on and utilization of these parts should be strengthened. Through the application of PLS-DA, it was found that linalool, cineole, and (*d*)-limone significantly contribute to the flavor of three species of the genus *Amomum*. In summary, these findings provide a theoretical basis for characterizing specific flavors associated with *A. tsaoko*, *A. paratsaoko,* and *A. koenigii*.

## Data Availability

The original contributions presented in the study are included in the article/Supplementary Materials, further inquiries can be directed to the corresponding author.

## Supplementary Materials

The following supporting information can be downloaded at: https://www.mdpi.com/article/10.3390/foods13121925/s1, Figure S1. The fruits and leaves of three species of genus *Amomum*. Figure S2. Cross validation and permutation tests of PLS–DA model. A. Cross validation; B. Permutation tests. Table S1. Identification of compounds in the fruits and leaves of three species of the genus *Amomum* using HS–SPME–GC–TOF–MS. Table S2. The main volatile metabolites in the leaves of three species of genus *Amomum.* Table S3. The main volatile metabolites in the fruits of three species of genus *Amomum.*

## References

[1] Ji K.L., Fan Y.Y., Ge Z.P., Sheng L., Xu Y.K., Gan L.S., Li J.Y., Yue J.M. (2019). Maximumins A-D, Rearranged Labdane-Type Diterpenoids with Four Different Carbon Skeletons from *Amomum maximum*. J. Org. Chem..

[2] Cai R., Yue X., Wang Y., Yang Y., Sun D., Li H., Chen L. (2021). Chemistry and bioactivity of plants from the genus *Amomum*. J. Ethnopharmacol..

[3] Subba B., Seling T.R., Kandel R.C., Phuyal G.P. (2017). Assessment of Antimicrobial and Antioxidant Activities of *Amomum subulatum* Roxb. Of Nepal. Asian J. Pharm. Clin. Res..

[4] Nurcholis W., Sya’bani Putri D.N., Husnawati H., Aisyah S.I., Priosoeryanto B.P. (2021). Total flavonoid content and antioxidant activity of ethanol and ethyl acetate extracts from accessions of *Amomum compactum* fruits. Ann. Agric. Sci..

[5] Kim J.G., Jang H., Le T.P.L., Hong H.R., Lee M.K., Hong J.T., Lee D., Hwang B.Y. (2019). Pyranoflavanones and Pyranochalcones from the Fruits of *Amomum tsao-ko*. J. Nat. Prod..

[6] Lee S., Lee J.C., Subedi L., Cho K.H., Kim S.Y., Park H.J., Kim K.H. (2019). Bioactive compounds from the seeds of *Amomum tsaoko* Crevost et Lemaire, a Chinese spice as inhibitors of sphingosine kinases, SPHK1/2. RSC Adv..

[7] Choi H.G., Je I.G., Kim G.J., Choi H., Kim S.H., Kim J.A., Lee S.H. (2015). Anti-allergic Inflammatory Activities of Compounds of Amomi Fructus. Nat. Prod. Commun..

[8] Kim Y.Y., Je I.G., Kim M.J., Kang B.C., Choi Y.A., Baek M.C., Lee B., Choi J.K., Park H.R., Shin T.Y. (2017). 2-Hydroxy-3-methoxybenzoic acid attenuates mast cell-mediated allergic reaction in mice via modulation of the FcepsilonRI signaling pathway. Acta Pharmacol Sin..

[9] Yang S., Xue Y., Chen D., Wang Z. (2022). *Amomum tsao-ko* Crevost & Lemarié: A comprehensive review on traditional uses, botany, phytochemistry, and pharmacology. Phytochem. Rev..

[10] Li Y., Lin Y., Shin S., Yu L., Yi W. (2010). Studies on the Antioxidant Components and Activities of the Methanol Extracts of Commercially Grown Hemerocallis *Fulva* L. (Daylily) in Taiwan. J. Food Biochem..

[11] Chinese Pharmacopoeia Commission (2020). Pharmacopoeia of the Peoples Republic of China.

[12] Hong S.S., Lee J.H., Choi Y.H., Jeong W., Ahn E.K., Lym S.H., Oh J.S. (2015). Amotsaokonal A–C, benzaldehyde and cycloterpenal from *Amomum tsao*-*ko*. Tetrahedron Lett..

[13] Wen H., Yang T., Yang W., Yang M., Wang Y., Zhang J. (2023). Comparison of Metabolites and Species Classification of Thirteen *Zingiberaceae* Spices Based on GC-MS and Multi-Spectral Fusion Technology. Foods.

[14] Fernie A.R., Pichersky E. (2015). Focus Issue on Metabolism: Metabolites, Metabolites Everywhere.

[15] Theis N., Adler L.S. (2012). Advertising to the enemy: Enhanced floral fragrance increases beetle attraction and reduces plant reproduction. Ecology.

[16] Issa M.Y., Mohsen E., Younis I.Y., Nofal E.S., Farag M.A. (2020). Volatiles distribution in jasmine flowers taxa grown in Egypt and its commercial products as analyzed via solid-phase microextraction (SPME) coupled to chemometrics. Ind. Crops Prod..

[17] Guan W., Li S., Yan R., Tang S., Quan C. (2007). Comparison of essential oils of clove buds extracted with supercritical carbon dioxide and other three traditional extraction methods. Food Chem..

[18] Chen J., Wang W., Kong J., Yue Y., Dong Y., Zhang J., Liu L. (2022). Application of UHPLC-Q-TOF MS based untargeted metabolomics reveals variation and correlation amongst different tissues of *Eucommia ulmoides* Oliver. Microchem. J..

[19] Yisimayili Z., Chao Z. (2022). A review on phytochemicals, metabolic profiles and pharmacokinetics studies of the different parts (juice, seeds, peel, flowers, leaves and bark) of *pomegranate* (*Punica granatum* L.). Food Chem..

[20] Ch R., Chevallier O., McCarron P., McGrath T.F., Wu D., Nguyen Doan Duy L., Kapil A.P., McBride M., Elliott C.T. (2021). Metabolomic fingerprinting of volatile organic compounds for the geographical discrimination of rice samples from China, Vietnam and India. Food Chem..

[21] Li Q., Ma X., Cheng J., Luo Y. (2013). Quantitative studies of floral color and floral scent. Biodivers. Sci..

[22] Zhou X., Zhu S., Wei J., Zhou Y. (2023). Volatile metabolomics and chemometric study provide insight into the formation of the characteristic cultivar aroma of *Hemerocallis*. Food Chem..

[23] Liu P., Zheng P., Gong Z., Feng L., Gao S., Wang X., Teng J., Zheng L., Liu Z. (2020). Comparing characteristic aroma components of bead-shaped green teas from different regions using headspace solid-phase microextraction and gas chromatography-mass spectrometry/olfactometry combined with chemometrics. Eur. Food Res. Technol..

[24] Zidi K., Kati D.E., Bachir-bey M., Genva M., Fauconnier M.L. (2021). Comparative Study of Fig Volatile Compounds Using Headspace Solid-Phase Microextraction-Gas Chromatography/Mass Spectrometry: Effects of Cultivars and Ripening Stages. Front. Plant Sci..

[25] Wang Z., Wang S., Liao P., Chen L., Sun J., Sun B., Zhao D., Wang B., Li H. (2022). HS-SPME Combined with GC-MS/O to Analyze the Flavor of Strong Aroma Baijiu Daqu. Foods.

[26] Fiehn O., Kind T., Weckwerth W. (2007). Metabolite Profiling in Blood Plasma. Metabolomics: Methods and Protocols.

[27] Fiehn O., Wohlgemuth G., Scholz M., Kind T., Lee D.Y., Lu Y., Moon S., Nikolau B. (2008). Quality control for plant metabolomics: Reporting MSI-compliant studies. Plant J..

[28] Szymańska E., Saccenti E., Smilde A.K., Westerhuis J.A. (2011). Double-check: Validation of diagnostic statistics for PLS-DA models in metabolomics studies. Metabolomics.

[29] Qin H., Wang Y., Yang W., Yang S., Zhang J. (2021). Comparison of metabolites and variety authentication of *Amomum tsao-ko* and *Amomum paratsao-ko* using GC-MS and NIR spectroscopy. Sci. Rep..

[30] Cai Z.M., Peng J.Q., Chen Y., Tao L., Zhang Y.Y., Fu L.Y., Long Q.D., Shen X.C. (2021). 1, 8-Cineole: A review of source, biological activities, and application. J. Asian Nat. Prod. Res..

[31] Caboni P., Ntalli N.G., Aissani N., Cavoski I., Angioni A. (2012). Nematicidal activity of (*E, E*)-2, 4-decadienal and (*E*)-2-decenal from *Ailanthus altissima* against *Meloidogyne javanica*. J. Agric. Food Chem..

[32] An Q., Ren J.N., Li X., Fan G., Qu S.S., Song Y., Li Y., Pan S.Y. (2021). Recent updates on bioactive properties of linalool. Food Funct..

[33] Erasto P., Viljoen A.M. (2008). Limonene–A review: Biosynthetic, ecological and pharmacological relevance. Nat. Prod. Commun..

[34] Ma D., Lin T., Zhao H., Li Y., Wang X., Di S., Liu Z., Liu M., Qi P., Zhang S. (2024). Development and comprehensive SBSE-GC/Q-TOF-MS analysis optimization, comparison, and evaluation of different mulberry varieties volatile flavor. Food Chem..

[35] Li G., Lu Q., Wang J., Hu Q., Liu P., Yang Y., Li Y., Tang H., Xie H. (2021). Correlation Analysis of Compounds in Essential Oil of *Amomum tsaoko* Seed and Fruit Morphological Characteristics, Geographical Conditions, Locality of Growth. Agronomy.

[36] Hou Z.W., Wang Y.J., Xu S.S., Wei Y.M., Bao G.H., Dai Q.Y., Deng W.W., Ning J.M. (2020). Effects of dynamic and static withering technology on volatile and nonvolatile components of Keemun black tea using GC-MS and HPLC combined with chemometrics. LWT.

[37] Zhang S.S., Guo S., Zheng Z.J., Liu S.J., Hou Y.F., Ho C.T., Bai N.S. (2021). Characterization of volatiles in *Allium tenuissimum* L. flower by headspace-gas chromatography-olfactometry-mass spectrometry, odor activity values, and the omission and recombination experiments. LWT.

[38] Pu Z.H., Wang B.S., Zhang S.Y., Sun F.H., Dai M. (2022). A review on quality control, toxicity and clinical application of *Amomum tsao-ko* Crevost & Lemarié. Pharmacol. Res.-Mod. Chin. Med..

[39] Yang Z., Xu S., Zhang W., An T., Liu L. (2019). Analysis on the content and main chemical components of volatile oils from *Amomum tsao-ko* Stems and Leaves in Yunnan province. J. Chin. Med. Mater..

[40] Gilles M., Zhao J., An M., Agboola S. (2010). Chemical composition and antimicrobial properties of essential oils of three Australian *Eucalyptus* species. Food Chem..

[41] Zhao S., Zhang D. (2014). Supercritical CO_2_ extraction of *Eucalyptus* leaves oil and comparison with Soxhlet extraction and hydro-distillation methods. Sep. Purif. Technol..

[42] Park Y.J., Baskar T.B., Yeo S.K., Arasu M.V., Al-Dhabi N.A., Lim S.S., Park S.U. (2016). Composition of volatile compounds and in vitro antimicrobial activity of nine *Mentha* spp.. SpringerPlus.

[43] González-Mas M.C., Rambla J.L., López-Gresa M.P., Blázquez M.A., Granell A. (2019). Volatile Compounds in Citrus Essential Oils: A Comprehensive Review. Front. Plant Sci..

[44] Liang M., Zhang Z., Wu Y., Wang R., Liu Y. (2023). Comparison of *Amomum tsaoko* crevost et Lemaire from four regions via headspace solid-phase microextraction: Variable optimization and volatile characterization. Ind. Crops Prod..

[45] Vyry Wouatsa N.A., Misra L., Venkatesh Kumar R. (2014). Antibacterial Activity of Essential Oils of Edible Spices, *Ocimum canum* and *Xylopia aethiopica*. J. Food Sci..

[46] Zhogoleva A., Alas M., Rosenvald S. (2023). Characterization of odor-active compounds of various pea preparations by GC-MS, GC-O, and their correlation with sensory attributes. Future Foods.

[47] Wen S., Sun L., Zhang S., Chen Z., Chen R., Li Z., Lai X., Zhang Z., Cao J., Li Q. (2023). The formation mechanism of aroma quality of green and yellow teas based on GC-MS/MS metabolomics. Food Res. Int..

[48] Giuliani C., Bottoni M., Santagostini L., Spada A., Papini A., Milani F., Fico G. (2023). *Teucrium fruticans* L., a Multi-Scale Study: From Trichomes to Essential Oil. Chem. Biodivers..

[49] Liu N., Shen S., Huang L., Deng G., Wei Y., Ning J., Wang Y. (2023). Revelation of volatile contributions in green teas with different aroma types by GC-MS and GC-IMS. Food Res. Int..

