# Supplementary material for: Analysis of the Volatile Components in Different Parts of Three Species of the Genus Amomum via Combined HS–SPME–GC–TOF–MS and Multivariate Statistical Analysis
Source: Foods. 2024 Jun 18;13(12):1925. doi: 10.3390/foods13121925 (PMC11202860; doi:10.3390/foods13121925)
Supplement: Supplementary file 1 [file foods-13-01925-s001.zip › foods-2991685-supplementary.pdf]

Article title: Analysis of the volatile components in different parts of three species of the genus *Amomum* via combined HS-SPME-GC-TOF-MS and multivariate statistical analysis

Authors: Jingjing Gu, Meiquan Yang, Mingju Qi, Tianmei Yang, Li Wang, Weize Yang, Jinyu Zhang

The following Supporting Information is available for this article:

Figure S1. The fruits and leaves of three species of genus *Amomum*

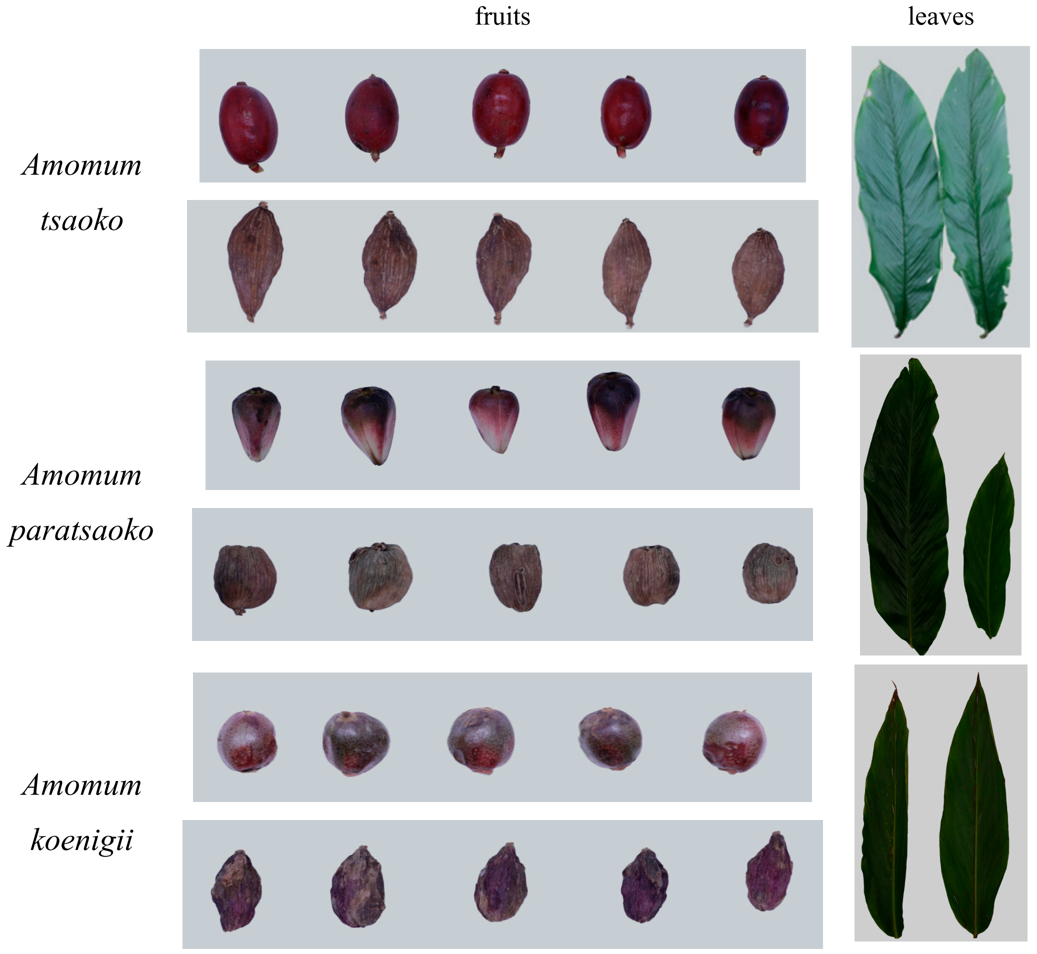

Table S1. Identification of compounds in the fruits and leaves of three species of the genus *Amomum* using HS-SPME-GC-TOF-MS.

name	CAS	Class	RT	PF			PL			KF			KL			TF			TL		
				PF1	PF2	PF3	PL1	PL2	PL3	KF1	KF2	KF3	KL1	KL2	KL3	TF1	TF2	TF3	TL1	TL2	TL3
2-aminoethanol	141-43-5	Others	1.68	0.00	0.00	0.00	89400143946.00	85703185517.00	0.00	0.00	0.00	0.00	0.00	0.00	0.00	0.00	0.00	0.00	0.00	0.00	0.00
Ethanol	64-17-5	Alcohols	3.97	1018246246.00	833029512.00	0.00	0.00	0.00	0.00	10521551050.00	23062994558.00	9855009738.00	0.00	0.00	0.00	1142172635.00	2025045315.00	2176308566.00	0.00	0.00	0.00
N-butyl alcohol	71-36-3	Alcohols	10.10	0.00	0.00	0.00	0.00	0.00	0.00	0.00	0.00	0.00	174910461.00	113654631.00	197019463.00	0.00	0.00	0.00	4277054.00	38155299.00	9705306.00
(*D*)-limonene	5989-27-5	Terpenoids	11.52	0.00	0.00	0.00	0.00	0.00	0.00	0.00	0.00	0.00	0.00	0.00	0.00	0.00	0.00	0.00	18489805216.00	28034464140.00	29967357174.00
Cineole	470-82-6	Terpenoids	11.88	117641404785.00	64396951509.00	77797751824.00	341726761208.00	118827397889.00	103781469824.00	129579984482.00	148263195704.00	119671930268.00	3872903249.00	1874753452.00	2309611531.00	851225966571.00	289068761208.00	648193761208.00	267798761208.00	77601114372.00	301296557135.00
N-tridecane	629-50-5	Hydrocarbons	14.85	1056636005.00	567657899.00	794274664.00	547380260.00	592189327.00	668469177.00	229231868.00	222467253.00	279079984.00	0.00	297048502.00	297234762.00	269931286.00	425883500.00	555444665.00	1196665951.00	1233164493.00	1003131153.00
Nonanal	124-19-6	Aldehyde	17.39	0.00	0.00	0.00	0.00	0.00	0.00	0.00	0.00	0.00	0.00	0.00	0.00	6377047870.00	2428218262.00	5659193813.00	9537611946.00	18783367733.00	0.00
N-tetradecane	629-59-4	Hydrocarbons	17.67	213751741.00	25044667.00	189267385.00	1527776524.00	1424997065.00	1670750685.00	294851788.00	84755408.00	65901120.00	390895057.00	156972882.00	141357904.00	0.00	0.00	0.00	1625456793.00	1617866995.00	741424199.00
Acetic acid	64-19-7	Carboxylic acids	19.09	5562952257.00	3460595017.00	3877506823.00	491265125.00	16761476860.00	17444672722.00	4376641329.00	6376584298.00	4563853288.00	0.00	0.00	0.00	0.00	0.00	0.00	0.00	0.00	0.00
2-ethyl-1-hexanol	104-76-7	Alcohols	20.10	0.00	0.00	0.00	0.00	0.00	0.00	0.00	0.00	0.00	291100513.00	0.00	124218752.00	0.00	0.00	0.00	0.00	0.00	0.00
Benzaldehyde	100-52-7	Aldehyde	20.65	4216968634.00	0.00	86234081899.00	0.00	0.00	0.00	2558206628.00	2740139351.00	3531905041.00	4624185177.00	1594081657.00	1495127263.00	1914951620.00	2190826270.00	2689163862.00	11527335175.00	9233400374.00	12677903853.00
Butyrolactone	96-4	Carboxyli	23.20	0.00	0.00	0.00	52182691	70309883	62781544	71802422	90709103	83893148	83777598	80318677	11137626	0.00	0.00	0.00	0.00	17885911	32566011

	8-0	c acids					3.00	2.00	3.00	3.00	4.00	8.00	.00	.00	3.00					07.00	2.00
Naphthalene	91-20-3	Hydrocarbons	25.78	0.00	0.00	0.00	0.00	0.00	0.00	0.00	0.00	0.00	276884967.00	575739689.00	172187244.00	0.00	0.00	0.00	0.00	0.00	0.00
Benzyllcohol	100-51-6	Alcohols	28.84	408149911.00	158327295.00	185096295.00	0.00	0.00	0.00	523913805.00	353428745.00	744779068.00	0.00	138836065.00	627145678.00	0.00	0.00	0.00	471673248.00	483027916.00	347878630.00
2-propen-1-one, 1-(4-morpholinyl)-	5117-12-4	Carboxylic acids	32.58	0.00	0.00	0.00	119131817.00	1072086864.00	83369645.00	0.00	0.00	0.00	0.00	0.00	0.00	0.00	0.00	0.00	0.00	0.00	0.00
Caprolactam	105-60-2	Terpenoids	34.99	0.00	0.00	0.00	0.00	0.00	0.00	0.00	0.00	0.00	165946039.00	61814683.00	69494693.00	0.00	0.00	0.00	0.00	0.00	0.00
N-propanol	71-23-8	Alcohols	6.70	0.00	0.00	0.00	0.00	0.00	0.00	0.00	0.00	0.00	0.00	7436463.00	72929735.00	0.00	0.00	0.00	151622712.00	170369829.00	12966089.00
4-xylene	106-42-3	Hydrocarbons	9.41	27536717.00	0.00	13026690.00	0.00	0.00	0.00	0.00	276403795.00	128256935.00	0.00	43675709.00	0.00	0.00	0.00	0.00	2478180075.00	87810802.00	49416256.00
N-undecane	1120-21-4	Hydrocarbons	8.50	0.00	0.00	0.00	0.00	0.00	0.00	0.00	0.00	0.00	18025381187.00	1562577774.00	948031012.00	0.00	0.00	0.00	780934630.00	0.00	915348222.00
Formaldehyde	50-00-0	Aldehyde	1.79	0.00	0.00	0.00	0.00	0.00	0.00	0.00	0.00	0.00	0.00	0.00	0.00	0.00	0.00	0.00	16757997309.00	0.00	16875897731.00
Acetaldehyde	75-07-0	Aldehyde	1.97	98135383.00	187045685.00	135631161.00	146438102.00	235960337.00	115477657.00	132650301.00	160306657.00	181053029.00	5151648428.00	191412489.00	269312229.00	44155158.00	0.00	138880723.00	121478333.00	202577671.00	185134109.00
Methyl cetate	79-20-9	Carboxylic acids	2.58	10961456813.00	9948700505.00	8265036180.00	6029753324.00	5156136279.00	6586527840.00	12557773883.00	8744073984.00	16681051481.00	11850077196.00	3803795177.00	3379235708.00	3145062780.00	2989760691.00	4553417896.00	6349021688.00	7448336039.00	7261613093.00
2-chloroethanol	107-07-3	Alcohols	3.38	120383085366.00	216103000000.00	85752036315.00	118244000000.00	107786162563.00	0.00	0.00	0.00	0.00	52660639313.00	90872572026.00	22873707455.00	0.00	0.00	0.00	0.00	0.00	0.00
Diacetyl	431-	Ketones	4.75	0.00	0.00	0.00	41997105	0.00	56076188	0.00	0.00	0.00	0.00	0.00	0.00	0.00	0.00	0.00	0.00	0.00	0.00

	03-8						4.00		8.00												
Tricyclene	508-32-7	Terpenoids	5.44	0.00	12696739.00	12663917.00	0.00	0.00	0.00	2815086646.00	4328305541.00	2028090662.00	195315796.00	229904534.00	72745090.00	165028154.00	86918713.00	124195805.00	512205374.00	392928449.00	449974049.00
1,5-dimethyl-6-oxa-bicyclo[3.1.0]hexane	82461-31-2	Others	5.59	150501459.00	57171637.00	79426103.00	0.00	0.00	0.00	0.00	0.00	0.00	0.00	0.00	0.00	0.00	0.00	0.00	0.00	0.00	0.00
Alpha-pinene	80-56-8	Terpenoids	5.88	84809696524.00	63590521397.00	68127032697.00	0.00	57392946286.00	45078828189.00	26131114303.00	25355278806.00	16343707614.00	50788206174.00	45168121548.00	27441158393.00	0.00	144975000000.00	172988000000.00	25504370073.00	51841864437.00	79968316601.00
Chloroform	67-66-3	Others	5.92	9558313443.00	6587412215.00	7565364249.00	16659462249.00	5967538776.00	4389749053.00	2964202765.00	2888561877.00	0.00	0.00	0.00	0.00	253930174898.00	18602733081.00	19011645129.00	0.00	0.00	0.00
Beta-thujene, (+/-)-	28634-89-1	Hydrocarbons	6.06	8444199761.00	4770076497.00	5000975700.00	16519233530.00	6260578269.00	3845642029.00	2120587857.00	1732084304.00	1259764907.00	0.00	0.00	0.00	39701673996.00	12911500989.00	17314217100.00	0.00	0.00	0.00
(*E*)-Crotonaldehyde	123-73-9	Aldehyde	6.43	66517472.00	54006193.00	75808516.00	0.00	117646769.00	7054171130.00	65910451.00	131196286.00	88016227.00	111212814.00	35596847.00	432037198.00	0.00	0.00	0.00	0.00	0.00	0.00
Alpha-fenchene	471-84-1	Terpenoids	6.92	0.00	0.00	0.00	0.00	203846964.00	257878216.00	211743340.00	72230054.00	76585765.00	119074346.00	88974550.00	43625672.00	0.00	0.00	0.00	83840621.00	68650968.00	96318082.00
Camphene	79-92-5	Terpenoids	7.16	671756238.00	0.00	431158149.00	0.00	0.00	0.00	53398231917.00	74675036880.00	42149424672.00	0.00	0.00	0.00	11306510788.00	2571270695.00	4066328739.00	7366805635.00	5935830345.00	7141974195.00
Hexanal	66-25-1	Aldehyde	7.81	1093853628.00	1674375849.00	1224704647.00	2133682045.00	2438011310.00	2008114467.00	401705048.00	415514294.00	530771202.00	13207143010.00	4446302187.00	6518423643.00	4141885931.00	1750215022.00	6467815377.00	21351904645.00	31608829359.00	56564475701.00
1-octanol	111-87-5	Alcohols	21.80	17787245472.00	141249000000.00	108532696955.00	174082560.00	671751652.00	2450423206.00	0.00	0.00	0.00	0.00	0.00	0.00	0.00	0.00	0.00	7841010118.00	859026588.00	514630089.00
Sabinene	3387-41-5	Terpenoids	8.99	61320607249.00	37455524251.00	44205083457.00	95408707790.00	29441130916.00	28999165150.00	2895566923.00	1577739680.00	1894758296.00	873025417.00	616502607.00	120560457.00	129882377074.00	79343810825.00	78973165415.00	1515813957.00	2218581322.00	2064571052.00
Carene	1346	Terpenoi	9.89	49023532	13561496	24132913	0.00	21752769	20803741	0.00	0.00	0.00	0.00	0.00	0.00	40108252	0.00	43355818	0.00	35811642	41776201

	6-78-9	ds		9.00	6.00	.00		4.00	0.00							30.00		50.00		.00	.00
1-methoxyoctane	929-56-6	Ethers	10.21	75849927.00	0.00	71350332.00	0.00	0.00	0.00	0.00	0.00	0.00	0.00	0.00	0.00	61277596.00	3758587392.00	0.00	0.00	0.00	0.00
Alpha-phellandrene	99-83-2	Terpenoids	10.43	14779147451.00	2292892057.00	111011088.00	98268004.00	8725546073.00	4688371195.00	286728461.00	438003249.00	257248101.00	133056469.00	0.00	40649495.00	99886404543.00	558296381.00	77257774798.00	140783524.00	327765063.00	326247534.00
Myrcene	123-35-3	Terpenoids	10.53	19661935531.00	5764800539.00	7252040004.00	45664767698.00	16917856333.00	14007124647.00	927127789.00	1514034779.00	877499899.00	3089915861.00	5022372783.00	5486503785.00	118124350814.00	36771812079.00	84980201716.00	9504862469.00	6482582579.00	6279609581.00
N-heptanal	111-71-7	Aldehyde	11.17	431992578.00	1214321856.00	1312491811.00	107258705.00	142391289.00	138544122.00	54593030.00	70356022.00	504163930.00	122585666.00	131917200.00	146465065.00	1083784994.00	1249750597.00	618224813.00	702263688.00	875353801.00	1256673216.00
l-limonene	5989-54-8	Terpenoids	11.51	60434328148.00	9140700423.00	34030448890.00	137241000000.00	33127450110.00	31765910227.00	44598826180.00	47798697016.00	34299346219.00	0.00	22754857379.00	10301756235.00	340655347111.00	0.00	212881000000.00	0.00	0.00	0.00
Beta-phellandrene	555-10-2	Terpenoids	11.77	7885340058.00	0.00	3140544570.00	0.00	4773873461.00	4315840815.00	1169943616.00	1338627006.00	942806763.00	0.00	0.00	0.00	0.00	0.00	0.00	0.00	0.00	0.00
N-dodecane	112-40-3	Hydrocarbons	11.79	0.00	0.00	0.00	0.00	0.00	0.00	0.00	0.00	0.00	3670518484.00	1409972198.00	918452842.00	0.00	0.00	0.00	0.00	0.00	0.00
Ocimene trans-beta-form	3779-61-1	Terpenoids	12.79	6781264978.00	4123556040.00	6693903631.00	33100824505.00	4328382959.00	5201116385.00	501012524.00	407003775.00	383925865.00	2135518730.00	2675496366.00	747992882.00	23792013436.00	14265651554.00	8627189260.00	0.00	228835988.00	330792420.00
Gamma-terpinene	99-85-4	Terpenoids	13.01	6257940165.00	2567972478.00	3199647109.00	11764797470.00	7532984342.00	4373638752.00	4322058249.00	3711672274.00	1999184559.00	2365349799.00	1949281682.00	749054179.00	25947674587.00	9012998679.00	11059272644.00	97100157.00	110378968.00	110683904.00
Alpha-ocimene	502-99-8	Terpenoids	13.29	54909682072.00	12146437533.00	0.00	281310000000.00	0.00	96052885087.00	0.00	0.00	0.00	0.00	0.00	0.00	0.00	0.00	90809112851.00	0.00	0.00	0.00
O-cymene	527-84-4	Terpenoids	13.74	0.00	3589957063.00	2046962599.00	0.00	0.00	0.00	0.00	0.00	0.00	0.00	0.00	0.00	0.00	0.00	0.00	0.00	0.00	0.00
Terpinolene	586-	Terpenoi	14.13	90746390	72416440	0.00	0.00	16457007	11383895	0.00	54639205	25293446	67767486	0.00	17708843	0.00	0.00	0.00	0.00	0.00	0.00

	62-9	ds		7.00	7.00			10.00	74.00		18.00	59.00	4.00		9.00						
Acetoin	513-86-0	Ketones	14.20	867075519.00	155399022.00	0.00	0.00	0.00	0.00	871006503.00	1176286534.00	651330735.00	396649203.00	178153250.00	505435603.00	0.00	400409917.00	304708365.00	57143080.00	49025598.00	44090660.00
Octanal	124-13-0	Aldehyde	14.40	77176249691.00	261928000000.00	0.00	0.00	0.00	0.00	1630329159.00	986175414.00	0.00	0.00	0.00	0.00	6602760941.00	15860937546.00	6067921730.00	0.00	0.00	0.00
Guanidine	113-00-8	Others	13.68	0.00	0.00	0.00	0.00	0.00	0.00	0.00	40823122.00	135602.00	0.00	0.00	0.00	0.00	0.00	0.00	0.00	0.00	0.00
3-o-methylgallic acid	3934-84-7	Phenols	21.73	0.00	0.00	0.00	0.00	0.00	0.00	0.00	0.00	0.00	0.00	1300011732.00	1065283062.00	0.00	0.00	0.00	0.00	0.00	0.00
1,3,7-nonatriene, 4,8-dimethyl-, (e)-	19945-61-0	Terpenoids	14.96	33393481.00	3687386.00	0.00	374122473.00	125517505.00	226693318.00	26566114084.00	26470747164.00	8657706811.00	14404834409.00	11027275906.00	4698456494.00	2062888081.00	107743467.00	243121618.00	0.00	61348886.00	69302062.00
Methyl lactate	2155-30-8	Carboxylic acids	15.27	139992610.00	19300199.00	69931096.00	0.00	0.00	0.00	553630655.00	796337151.00	279063723.00	271008406.00	136040240.00	258981649.00	0.00	0.00	0.00	0.00	0.00	0.00
Methylheptenone	110-93-0	Ketones	15.81	222028409.00	103200987.00	142235772.00	0.00	0.00	0.00	275461002.00	269884431.00	361052378.00	0.00	1800817218.00	849938985.00	1509222700.00	4357902265.00	4570131323.00	2486224242.00	1289232816.00	0.00
Trans-2-decenal	3913-81-3	Aldehyde	22.96	0.00	4336695900.00	6579166194.00	0.00	0.00	0.00	0.00	0.00	0.00	0.00	0.00	0.00	0.00	420188000000.00	1500786533.00	0.00	0.00	0.00
1,1-dimethoxyoctane	10022-28-3	Aldehyde	16.72	176244354.00	1983641832.00	3699270195.00	0.00	0.00	0.00	0.00	0.00	0.00	0.00	0.00	0.00	0.00	6964691.00	10596967.00	0.00	0.00	0.00
Tridecane, 3-methyl-	6418-41-3	Hydrocarbons	16.75	0.00	0.00	0.00	221805262.00	548360766.00	676464196.00	0.00	0.00	0.00	0.00	0.00	0.00	0.00	0.00	0.00	437298366.00	266022690.00	296228742.00
Alloocimene, (4e,6z)-	7216-56-0	Terpenoids	16.80	0.00	0.00	0.00	1178678362.00	548360766.00	676464196.00	0.00	0.00	0.00	0.00	0.00	0.00	6659202778.00	162909357.00	388224526.00	0.00	0.00	0.00

Methyl glycolate	96-35-5	Carboxylic acids	17.12	12083237.00	9903396.00	16100326.00	0.00	0.00	0.00	11749613.00	11258324.00	35140275.00	1714476679.00	0.00	252371591.00	0.00	0.00	0.00	3806422.00	3335470.00	1854012.00
Octanoic acid, methyl ester	111-11-5	Carboxylic acids	17.33	91558230557.00	132004000000.00	98544724661.00	22893573004.00	35009302817.00	38235439833.00	0.00	0.00	0.00	19174364999.00	9215250297.00	14750207841.00	2294532310.00	2810978800.00	995263883.00	40763541250.00	27390941376.00	14589993776.00
Perillene	539-52-6	Terpenoids	18.07	0.00	0.00	0.00	52447179.00	2213526.00	2839589.00	0.00	0.00	0.00	0.00	0.00	0.00	210232575.00	92073996.00	244984344.00	0.00	0.00	0.00
Ethyl caprylate	106-32-1	Carboxylic acids	18.56	0.00	21311088.00	29030380.00	0.00	0.00	0.00	0.00	0.00	0.00	0.00	0.00	0.00	0.00	0.00	0.00	0.00	0.00	0.00
Cosmene	460-01-5	Hydrocarbons	18.75	991863478.00	283640544.00	560189594.00	7609163486.00	2006843855.00	2041567335.00	0.00	0.00	0.00	0.00	0.00	0.00	4381833398.00	2564197684.00	1967699869.00	0.00	0.00	0.00
Alpha-cubebene	17699-14-8	Terpenoids	19.11	6581558976.00	4479201736.00	4896113542.00	29552644519.00	17780083579.00	18252283298.00	0.00	0.00	0.00	0.00	0.00	0.00	14326366044.00	0.00	12600478380.00	0.00	0.00	0.00
1-methyloctyl acetate	14936-66-4	Carboxylic acids	19.28	0.00	0.00	0.00	0.00	0.00	0.00	481474294.00	1034351222.00	23190613727.00	0.00	0.00	0.00	238938430.00	1634737281.00	314622585.00	0.00	0.00	0.00
Octyl acetate	112-14-1	Carboxylic acids	19.64	16667347897.00	166864000000.00	34714894937.00	0.00	30731981.00	18942642.00	1414186127.00	711864562.00	3516960118.00	0.00	0.00	0.00	426467133.00	0.00	171080843.00	716229261.00	75352781.00	55169851.00
Copaene	3856-25-5	Terpenoids	19.98	40157215356.00	15445745305.00	26385609475.00	184216000000.00	107339206987.00	113350336607.00	571002776.00	448466512.00	563447470.00	481453277.00	0.00	780724681.00	86091418076.00	90955391774.00	59543346027.00	40716601954.00	21982378886.00	47249951131.00
Delorazepam [inn]	2894-67-9	Others	20.01	0.00	0.00	0.00	0.00	22697923492.00	25648574255.00	0.00	0.00	0.00	0.00	0.00	0.00	0.00	0.00	0.00	0.00	0.00	0.00
Methyl pelargonate	1731-84-6	Carboxylic acids	20.05	1533203186.00	7767069065.00	0.00	28643565442.00	12887385616.00	15237939000.00	92416816.00	93315561.00	290702413.00	1256784986.00	450169675.00	554039306.00	8858085532.00	0.00	5632257367.00	39591700278.00	52474745920.00	48045291003.00
2-Decanone	693-54-9	Ketones	19.60	0.00	0.00	0.00	0.00	0.00	0.00	4182865879.00	5290450113.00	4780830317.00	0.00	0.00	0.00	0.00	0.00	0.00	0.00	0.00	0.00

Decanal	112-31-2	Aldehyde	20.20	189255549639.00	180274000000.00	623947045284.00	4124405158.00	3854215740.00	4644260449.00	3624167773.00	1080785471.00	886008843.00	754282781.00	257589852.00	253827435.00	18215848819.00	24186921646.00	4114568477.00	8396659524.00	901095669.00	1226994989.00
Methyl malonate	108-59-8	Carboxylic acids	20.43	0.00	0.00	0.00	0.00	0.00	0.00	6045373.00	24275084.00	49632024.00	461391502.00	10324365.00	11747629.00	0.00	0.00	0.00	0.00	0.00	0.00
l-camphor	464-48-2	Terpenoids	20.53	0.00	0.00	0.00	0.00	0.00	0.00	44082808183.00	46389320339.00	22835453663.00	936677165.00	660250756.00	461259397.00	0.00	0.00	0.00	5274196167.00	4635042114.00	4866517333.00
Ethyl pyruvate	617-35-6	Carboxylic acids	19.54	0.00	0.00	0.00	0.00	0.00	0.00	0.00	0.00	0.00	0.00	0.00	0.00	0.00	29701862.00	22775.00	0.00	0.00	0.00
Beta-copaene	18252-44-3	Terpenoids	25.18	0.00	1808778313.00	3185552400.00	187511000000.00	103083777620.00	113293208759.00	0.00	0.00	0.00	0.00	0.00	0.00	116051214577.00	0.00	32308627347.00	0.00	0.00	0.00
Linalool	78-70-6	Terpenoids	21.50	3848059224.00	1486041789.00	0.00	19483845676.00	17916324743.00	31423493658.00	0.00	318156838639.00	227520311108.00	167850000000.00	191200823645.00	148863006400.00	0.00	92283246768.00	8379931757.00	52587100613.00	38976840093.00	33332963337.00
Alpha-pinocarvone	30460-92-5	Terpenoids	21.86	4463317873.00	144834000000.00	0.00	0.00	0.00	0.00	0.00	0.00	0.00	94100.00	117160259.00	527316173.00	0.00	5261799571.00	16264377389.00	3247777973.00	9225980820.00	9557834131.00
Beta-ylangene	20479-06-5	Terpenoids	22.03	0.00	0.00	0.00	5212456716.00	9222089.00	0.00	0.00	0.00	0.00	0.00	0.00	0.00	151536315.00	3799990673.00	182787929.00	0.00	2374413.00	5807204.00
Bornyl acetate	92618-89-8	Terpenoids	22.22	0.00	0.00	0.00	0.00	0.00	0.00	0.00	0.00	0.00	100864751.00	185657288.00	0.00	0.00	0.00	3025039983.00	20856948365.00	21026476841.00	28989667060.00
Beta-caryophyllene	87-44-5	Terpenoids	22.58	2238113474.00	0.00	1992645960.00	59480556038.00	35412273135.00	34980909287.00	0.00	0.00	0.00	72253256826.00	51397322222.00	13718242870.00	66856885919.00	58143941960.00	14427345104.00	3385875799.00	754711317.00	553288146.00
2-undecanone	112-12-9	Ketones	22.69	0.00	0.00	0.00	0.00	0.00	0.00	0.00	0.00	0.00	0.00	0.00	0.00	0.00	3941985986.00	1315409898.00	0.00	0.00	0.00

(+)-Aromadendrene	489-39-4	Terpenoids	23.21	0.00	0.00	0.00	32724166616.00	26121209216.00	19609146358.00	0.00	0.00	0.00	0.00	0.00	0.00	0.00	0.00	0.00	11557302231.00	0.00	6431357235.00
Beta-cubebene	13744-15-5	Terpenoids	21.17	0.00	0.00	0.00	0.00	0.00	0.00	0.00	0.00	0.00	0.00	0.00	0.00	0.00	29327027428.00	14232845351.00	0.00	0.00	0.00
2-Decanol	1120-06-5	Alcohols	20.29	0.00	0.00	0.00	0.00	546529772.00	252746507.00	0.00	0.00	0.00	0.00	0.00	0.00	0.00	0.00	0.00	0.00	0.00	0.00
Myrtenal, (-)-	18486-69-6	Terpenoids	23.31	0.00	0.00	0.00	137929860.00	0.00	164053337.00	0.00	0.00	0.00	0.00	0.00	0.00	0.00	0.00	0.00	0.00	0.00	0.00
Butanedioic acid, 1-ethyl 4-methyl ester	627-73-6	Carboxylic acids	23.51	0.00	0.00	0.00	0.00	0.00	0.00	84169168.00	73220448.00	257364578.00	0.00	0.00	0.00	0.00	0.00	0.00	0.00	0.00	0.00
l-Pinocarveol	547-61-5	Alcohols	24.04	637312301.00	1277549162.00	1356826495.00	0.00	0.00	0.00	0.00	0.00	0.00	0.00	272575768.00	339926680.00	9587742943.00	0.00	15601488019.00	10904442205.00	15303839594.00	16894606697.00
Humulene	6753-98-6	Terpenoids	24.28	3025496401.00	1612441433.00	2337768218.00	14837243318.00	6410085441.00	5916271619.00	151237542.00	117705020.00	278187526.00	3269499563.00	2310807683.00	1695516653.00	26637715390.00	16903734397.00	5035079835.00	3062219326.00	959080752.00	0.00
Butanedioic acid, diethyl ester	123-25-1	Carboxylic acids	24.50	0.00	0.00	0.00	0.00	0.00	0.00	0.00	0.00	0.00	6942888.00	4159508.00	5713353.00	0.00	0.00	0.00	55608993.00	60161388.00	94535183.00
Decyl acetate	112-17-4	Carboxylic acids	24.62	7856077037.00	83750166422.00	18446848649.00	0.00	0.00	0.00	0.00	0.00	0.00	0.00	0.00	0.00	0.00	0.00	0.00	0.00	0.00	0.00
.Gamma.-muurolene	30021-74-0	Terpenoids	24.74	4533914461.00	3323808333.00	3698191546.00	51108598273.00	31362462557.00	30218987132.00	0.00	0.00	0.00	857699949.00	456227203.00	266669397.00	132549921986.00	133785000000.00	97201381733.00	0.00	2685688143.00	3872176369.00
Ledene	2174	Terpenoi	24.89	0.00	0.00	0.00	0.00	0.00	0.00	35950915	43507174	0.00	0.00	0.00	0.00	0.00	0.00	0.00	0.00	0.00	0.00

	7-46-6	ds								4.00	8.00										
Alpha-terpineol	98-55-5	Terpenoids	25.02	7753407078.00	2400386173.00	3245967956.00	23903823996.00	6003277587.00	5370523070.00	11362596203.00	4292329793.00	2747817660.00	0.00	0.00	0.00	187697562314.00	0.00	112878000000.00	11175654999.00	12184557848.00	1606778669.00
Beta-selinene	17066-67-0	Terpenoids	25.42	0.00	0.00	0.00	0.00	0.00	0.00	47816348.00	0.00	147815781.00	531626475.00	0.00	31027163.00	0.00	0.00	0.00	0.00	0.00	0.00
Bicyclogermacren	24703-35-3	Terpenoids	25.75	0.00	0.00	0.00	72788985779.00	29429767653.00	33300508371.00	23503135040.00	26267459712.00	0.00	0.00	0.00	0.00	0.00	0.00	0.00	0.00	0.00	0.00
Alpha-farnesene	502-61-4	Terpenoids	26.10	648804757.00	607360691.00	0.00	0.00	0.00	0.00	0.00	0.00	0.00	2743596735.00	3518479272.00	926922454.00	0.00	0.00	0.00	0.00	0.00	0.00
1-Decanol	112-30-1	Alcohols	26.53	4639620913.00	87011258615.00	43155257764.00	5832997.00	0.00	49934119.00	2034194562.00	0.00	939592172.00	0.00	0.00	0.00	0.00	0.00	0.00	1152830800.00	346157848.00	0.00
Cuparene	16982-00-6	Terpenoids	26.62	0.00	0.00	0.00	0.00	0.00	0.00	0.00	0.00	0.00	0.00	80319611.00	227261435.00	0.00	0.00	0.00	0.00	0.00	0.00
N,N-Dibutylformamide	761-65-9	Carboxylic acids	26.73	0.00	0.00	0.00	26806867.00	46580938.00	44391939.00	0.00	25930115.00	84865965.00	0.00	6312896.00	324456370.00	0.00	0.00	0.00	401511178.00	450859916.00	495111894.00
Myrtenol	515-00-4	Terpenoids	27.15	224156817.00	163095697.00	394786523.00	0.00	0.00	0.00	460878688.00	554534420.00	0.00	0.00	0.00	0.00	14139276946.00	2113590453.00	6745237520.00	0.00	0.00	0.00
Methyl dodecanoate	111-82-0	Carboxylic acids	27.32	9300879192.00	6493068716.00	6221619805.00	1765632459.00	2714648776.00	2341558826.00	502432274.00	407809028.00	755678680.00	3723073358.00	377328235.00	480008435.00	0.00	0.00	0.00	7239583172.00	8968094783.00	5849320090.00
1,3,7,11-Tridecatetraene, 4,8,12-trimeth	62235-06-7	Terpenoids	27.39	0.00	0.00	0.00	0.00	0.00	0.00	338210076.00	349786376.00	0.00	0.00	1017536682.00	621867053.00	0.00	0.00	0.00	0.00	0.00	0.00

yl-, (3e,7e)-																					
1,6-Dimethyl-4-isopropyltetralin	483-77-2	Terpenoids	27.90	0.00	0.00	0.00	0.00	0.00	0.00	15621652.00	18042495.00	11194091.00	0.00	0.00	0.00	0.00	0.00	0.00	679597871.00	0.00	267224220.00
Methyl 3-phenylpropionate	103-25-3	Carboxylic acids	28.16	185032747.00	40068185.00	42164720.00	0.00	0.00	0.00	0.00	0.00	0.00	9348560.00	174005.00	0.00	0.00	0.00	0.00	0.00	0.00	0.00
Nerol	106-25-2	Terpenoids	28.32	0.00	1121690630.00	2213820350.00	0.00	0.00	0.00	0.00	1428299411.00	1135071787.00	0.00	0.00	0.00	4270724895.00	7222426301.00	8663735885.00	0.00	0.00	0.00
2-Dodecenal	4826-62-4	Aldehyde	28.53	0.00	6204005799.00	4103727430.00	0.00	0.00	0.00	0.00	0.00	0.00	0.00	0.00	0.00	0.00	189411000000.00	36045566842.00	0.00	0.00	0.00
2,2,4-Trimethyl-1,3-pentanediol diisobutyrate	6846-50-0	Alcohols	28.91	0.00	95458338.00	100367329.00	33044031.00	70794601.00	74267755.00	0.00	0.00	0.00	0.00	0.00	0.00	0.00	6966524.00	4857552.00	1727005.00	2841626.00	0.00
Lauryl acetate	112-66-3	Carboxylic acids	29.10	0.00	401504639.00	101943089.00	0.00	0.00	0.00	0.00	0.00	0.00	0.00	0.00	0.00	0.00	0.00	0.00	419013976.00	228009506.00	11114891.00
2-Phenylethanol	60-12-8	Alcohols	29.58	822582301.00	584909689.00	637842529.00	0.00	0.00	0.00	378293523.00	668635079.00	646245491.00	2048367913.00	556764937.00	543379325.00	0.00	0.00	0.00	38222955.00	52713527.00	552144279.00
3,4-Dihydrocadalene	21391-99-1	Hydrocarbons	29.63	164147596.00	118159093.00	137645247.00	880142462.00	616287438.00	592039550.00	0.00	0.00	0.00	408354007.00	119808178.00	99258283.00	698935354.00	1010855834.00	747408799.00	1422487282.00	0.00	1566706414.00
(3R,3aR,3bR,4S,7R,7aR)-4-Isopropyl-3,7-dimethylocta	38230-60-3	Alcohols	30.26	1622933573.00	0.00	1935543364.00	21709094561.00	7072595155.00	8438870538.00	0.00	0.00	0.00	0.00	0.00	0.00	52326164785.00	53576003144.00	24485892951.00	0.00	0.00	0.00

hydro-1H-cyclopenta[1,3]cyclopropa[1,2]benzen-3-ol																					
7-Methyl-4-methylidene-1-propan-2-yl-2,3-dihydro-1h-naphthalene	50277-34-4	Hydrocarbons	30.50	43220897.00	25514575.00	41234893.00	295960708.00	371628067.00	72116643.00	0.00	202013520.00	11507054.00	0.00	0.00	0.00	1026674.00	66053328.00	240216278.00	269264605.00	47796083.00	0.00
Cubebol	23445-02-5	Terpenoids	29.19	1183428119.00	0.00	1632667121.00	7984697465.00	4503723344.00	4738150394.00	0.00	0.00	0.00	0.00	0.00	0.00	23344831387.00	23386459249.00	6862928667.00	503297676.00	394212955.00	572704489.00
(-)-Beta-caryophyllene epoxide	1139-30-6	Terpenoids	31.05	0.00	0.00	0.00	1399543773.00	0.00	761765173.00	0.00	0.00	0.00	5553792128.00	3393222276.00	5723278025.00	3535946801.00	2141161771.00	3371469044.00	0.00	0.00	0.00
Methyl myristate	124-10-7	Carboxylic acids	31.59	2318381937.00	2896682370.00	2187281987.00	409810909.00	520217411.00	448379729.00	151684367.00	80920718.00	95811290.00	259589767.00	124636644.00	87493145.00	1152408512.00	1594272119.00	198534071.00	615279671.00	901362626.00	816516889.00
Nerolidol	7212-44-4	Terpenoids	32.19	5544523683.00	0.00	7183585055.00	7802547786.00	1366394903.00	2387491249.00	3048218997.00	2648651183.00	901995618.00	599068774.00	686140529.00	434768392.00	128898794118.00	47773832920.00	27141712937.00	643517538.00	522519597.00	419464067.00
Methyl cis-9-tetradecenoate	56219-06-8	Carboxylic acids	32.32	5286909538.00	5695703992.00	3765668583.00	0.00	0.00	0.00	260035206.00	163481134.00	87319711.00	0.00	0.00	0.00	0.00	0.00	0.00	90485312.00	126711294.00	95820765.00
3-Methyl-2-butanol	598-75-4	Alcohols	25.03	0.00	0.00	0.00	827393893.00	0.00	0.00	0.00	0.00	0.00	0.00	0.00	0.00	0.00	0.00	0.00	0.00	0.00	0.00
Methyl trans-cinnama	1754-62-7	Carboxylic acids	32.79	14805046139.00	3580140130.00	4588249227.00	683607186.00	794377189.00	456819194.00	825142451.00	595635089.00	530338899.00	0.00	0.00	0.00	1265756634.00	1013400952.00	0.00	0.00	0.00	0.00

te																					
Octanoic acid	124-07-2	Alcohols	32.87	1585373247.00	263159713.00	0.00	0.00	569295372.00	599543627.00	190721983.00	147995199.00	166337185.00	0.00	0.00	0.00	0.00	0.00	0.00	0.00	0.00	0.00
Methyl pentadecanoate	7132-64-1	Carboxylic acids	33.58	271064113.00	359344781.00	560604863.00	218312251.00	244462670.00	248070927.00	114469172.00	0.00	54499921.00	97816883.00	57023628.00	925129314.00	686923569.00	474601835.00	331000629.00	192064152.00	287306729.00	236958326.00
Spathulenol, (-)-	77171-55-2	Terpenoids	34.72	0.00	0.00	0.00	96827491.00	29209437.00	0.00	0.00	345874547.00	469991976.00	0.00	0.00	0.00	0.00	0.00	0.00	0.00	0.00	0.00
Ethyl cinnamate	103-36-6	Carboxylic acids	33.83	1083237766.00	506329683.00	593535158.00	0.00	0.00	0.00	31958686.00	0.00	463920220.00	113367028.00	0.00	763294770.00	0.00	0.00	0.00	504639449.00	398227181.00	0.00
2-Phenoxyethanol	122-99-6	Alcohols	34.07	14722344.00	0.00	3768716.00	0.00	0.00	0.00	0.00	0.00	0.00	0.00	0.00	0.00	0.00	0.00	0.00	0.00	49627072.00	66127045.00
T-Cadinol	5937-11-1	Terpenoids	34.65	0.00	0.00	0.00	3765239992.00	2214500728.00	2057386046.00	0.00	33176169.00	41520540.00	65670727.00	30079628.00	125864940.00	0.00	0.00	0.00	0.00	0.00	0.00
Methyl palmitate	112-39-0	Carboxylic acids	35.50	4269639323.00	5739217304.00	9904342904.00	2014589516.00	2088841137.00	3804236991.00	1148117761.00	751974335.00	860308379.00	1081654108.00	643869109.00	463155019.00	4465554579.00	2248787987.00	990002721.00	1532307893.00	3437238569.00	2664872251.00
T-muurolol	19912-62-0	Terpenoids	34.96	0.00	0.00	0.00	0.00	2949918583.00	2670305605.00	0.00	0.00	0.00	0.00	0.00	0.00	0.00	0.00	0.00	0.00	0.00	0.00
(*Z*)-methyl hexadec-11-enoate	822-05-9	Carboxylic acids	35.98	174985727.00	309641368.00	205082776.00	0.00	104814609.00	91422335.00	0.00	40055384.00	172771553.00	0.00	0.00	0.00	0.00	0.00	0.00	194368327.00	101854820.00	142824267.00
Methyl stearate	112-61-8	Carboxylic acids	39.09	94234005.00	131312898.00	177279310.00	46650344.00	48977666.00	69333641.00	43078518.00	17178119.00	41444838.00	31590303.00	18950967.00	0.00	333285283.00	370445631.00	126520303.00	45022387.00	124987262.00	116613986.00
Methyl oleate	112-	Carboxyli	39.43	19746494	0.00	20384369	10652181	90747920	0.00	11875016	0.00	14201835	0.00	0.00	0.00	0.00	0.00	0.00	0.00	0.00	0.00

	62-9	c acids		8.00		6.00	6.00	.00		7.00		5.00									
Methyl octadeca-9,12-dienoate	2462-85-3	Carboxylic acids	40.22	0.00	0.00	0.00	113950851.00	154201194.00	0.00	0.00	85250305.00	119815653.00	125630983.00	70071762.00	0.00	0.00	0.00	0.00	0.00	0.00	0.00
hydroquinone	123-31-9	Phenols	46.66	0.00	0.00	0.00	0.00	0.00	0.00	2026849018.00	2622121743.00	2112091871.00	372008801.00	405666312.00	244795467.00	0.00	0.00	0.00	70635575.00	59842655.00	0.00
Methyl formate	107-31-3	Carboxylic acids	2.18	222161936.00	253850214.00	248060356.00	216763189.00	113745478.00	157858489.00	61452363.00	97906827.00	154087767.00	1531219037.00	820769258.00	996051019.00	193694356.00	265274178.00	236720428.00	933194737.00	804042055.00	1016419600.00
1-Methoxy-2-methylbutane	62016-48-2	Ethers	2.36	0.00	0.00	0.00	126015938.00	25878838.00	12795733.00	185511713.00	286635312.00	76502505.00	81227384.00	74180972.00	38373312.00	0.00	0.00	0.00	0.00	0.00	0.00
Nitroethane	79-24-3	Others	3.36	4183169.00	197342329.00	207684638.00	0.00	0.00	0.00	256572662522.00	0.00	281774439761.00	0.00	0.00	0.00	0.00	0.00	0.00	0.00	0.00	0.00
Pentanal	110-62-3	Aldehyde	4.77	370531404.00	41548845.00	92148697.00	419971054.00	661594584.00	560761888.00	234300804.00	0.00	191876175.00	1233685155.00	608429876.00	1811279113.00	250553241.00	298851129.00	278434494.00	0.00	2804648850.00	3636074066.00
CrotonAldehyde	4170-30-3	Aldehyde	6.43	0.00	0.00	0.00	0.00	0.00	0.00	0.00	0.00	0.00	0.00	0.00	0.00	0.00	0.00	0.00	0.00	386513244.00	376783210.00
Camphene, (+)-	5794-03-6	Terpenoids	6.92	232893452.00	157615446.00	0.00	0.00	0.00	0.00	0.00	0.00	0.00	0.00	0.00	0.00	1264615764.00	520715720.00	585448510.00	0.00	0.00	0.00
Beta-pinene	127-91-3	Terpenoids	8.51	0.00	0.00	0.00	216885000000.00	112671740704.00	0.00	0.00	0.00	0.00	0.00	0.00	0.00	0.00	221629000000.00	369380000000.00	0.00	0.00	0.00
Methyl caproate	106-70-7	Carboxylic acids	11.29	5155439789.00	1895461813.00	2061115568.00	26317296164.00	47153638124.00	50166321615.00	196240299.00	115089343.00	731652094.00	23985817224.00	9540555617.00	9175707556.00	718989210.00	635920572.00	1799019883.00	0.00	0.00	0.00
Phenol, 3-(1,1-dimethylethyl)-	585-34-2	Hydrocarbons	12.23	0.00	0.00	0.00	0.00	0.00	0.00	0.00	0.00	0.00	9296216038.00	10415057343.00	0.00	0.00	0.00	0.00	0.00	0.00	0.00

Isoterpinolene	586-63-0	Hydrocarbons	14.14	0.00	0.00	0.00	0.00	0.00	0.00	0.00	0.00	0.00	0.00	0.00	0.00	9656920886.00	4425997199.00	4514344638.00	0.00	0.00	0.00
Heptyl acetate	112-06-1	Carboxylic acids	16.90	0.00	0.00	0.00	0.00	0.00	0.00	94164339.00	64493186.00	49371137.00	0.00	0.00	0.00	0.00	0.00	0.00	0.00	0.00	0.00
2-Octenal, (2e)-	2548-87-0	Aldehyde	18.33	6593702212.00	52208126631.00	47009274100.00	404448858.00	930495536.00	2091797066.00	433139062.00	405308121.00	550385753.00	233779851.00	132582732.00	297281888.00	43214630132.00	75839187221.00	21507492943.00	3801634103.00	5739488097.00	9993122150.00
Benzene, 4-ethenyl-1,2-dimethyl-	27831-13-6	Hydrocarbons	18.48	0.00	0.00	0.00	0.00	0.00	0.00	0.00	0.00	0.00	0.00	0.00	0.00	1377995711.00	1687351838.00	0.00	0.00	0.00	0.00
Vinyl acetate	108-05-4	Carboxylic acids	4.75	0.00	0.00	0.00	0.00	0.00	0.00	0.00	286262410.00	191876175.00	0.00	0.00	0.00	0.00	0.00	0.00	0.00	0.00	0.00
2-Nonanol	628-99-9	Alcohols	20.86	0.00	0.00	0.00	242526603.00	0.00	63638735.00	3592795745.00	6715851653.00	25257374126.00	0.00	0.00	0.00	3888371846.00	3743943887.00	818777042.00	0.00	0.00	0.00
Bicyclo(3.1.1)heptan-2-one, 6,6-dimethyl-	24903-95-5	Terpenoids	22.11	0.00	0.00	0.00	0.00	0.00	0.00	0.00	0.00	0.00	0.00	0.00	0.00	0.00	0.00	0.00	0.00	254883754.00	258644448.00
(-)-Beta-elemene	515-13-9	Terpenoids	22.44	0.00	0.00	0.00	0.00	0.00	0.00	4517087.00	0.00	104035650.00	0.00	0.00	0.00	0.00	0.00	0.00	0.00	0.00	0.00
Trinitrosotrimethylenetriamine	13980-04-6	Others	22.89	0.00	0.00	0.00	0.00	0.00	0.00	1226206992.00	0.00	535370023.00	0.00	0.00	0.00	0.00	0.00	0.00	0.00	0.00	0.00
Myrtenal	564-94-3	Terpenoids	23.31	195157905.00	0.00	787795652.00	0.00	0.00	0.00	0.00	0.00	0.00	0.00	0.00	0.00	15565401844.00	3719550761.00	10710375017.00	1963763408.00	0.00	5315391153.00
(1S,4S,4aS)-1-Isopropyl-4,7-dimethyl-1,2,	267665-20-3	Terpenoids	23.42	334089222.00	36172801.00	114184086.00	6216127612.00	1973640462.00	1911262107.00	0.00	0.00	0.00	0.00	0.00	0.00	2504083397.00	331423978.00	2178436072.00	0.00	0.00	0.00

3,4,4a,5-hexahydronaphthalene																					
Alloaromadendrene	25246-27-9	Terpenoids	23.73	30064075572.00	176043000000.00	0.00	70947621358.00	42403055737.00	38991102286.00	3697517267.00	2883654648.00	1952973670.00	3827183267.00	1795548747.00	272686278.00	0.00	0.00	0.00	0.00	0.00	0.00
1-Nonanol	143-08-8	Alcohols	24.23	392571031.00	1657336215.00	1429434012.00	0.00	0.00	0.00	0.00	0.00	0.00	0.00	0.00	0.00	0.00	0.00	0.00	0.00	0.00	0.00
N-Heptadecane	629-78-7	Hydrocarbons	25.06	1647765455.00	908467515.00	0.00	0.00	0.00	0.00	0.00	0.00	0.00	0.00	0.00	0.00	0.00	0.00	0.00	0.00	0.00	0.00
2-Dodecen-1-ol, (2e)	69064-37-5	Alcohols	31.89	0.00	0.00	0.00	0.00	0.00	0.00	0.00	0.00	0.00	0.00	0.00	0.00	0.00	0.00	0.00	0.00	1372719891.00	1613179608.00
Bicyclosesquiphellandrene	54324-03-7	Terpenoids	23.09	0.00	0.00	0.00	0.00	0.00	0.00	0.00	0.00	0.00	0.00	0.00	0.00	126757010.00	603884721.00	3547843592.00	0.00	0.00	0.00
(-)-Alpha-cadinene	24406-05-1	Terpenoids	27.04	173117352.00	90862835.00	113645307.00	6983232993.00	3602690694.00	3659154518.00	0.00	0.00	0.00	0.00	0.00	0.00	649448053.00	489769820.00	578058769.00	0.00	0.00	0.00
p-Cymen-8-ol	1197-01-9	Terpenoids	28.29	146355362.00	50573858.00	79760407.00	0.00	0.00	0.00	353601315.00	1428299411.00	142762829.00	1187752297.00	464878565.00	0.00	630797507.00	78554392.00	1129482334.00	4251993760.00	8433931437.00	7692819734.00
Methyl tridecanoate	1731-88-0	Carboxylic acids	29.50	63212635.00	50795869.00	31108383.00	5859235.00	20924335.00	16348499.00	0.00	0.00	0.00	14314541.00	15974923.00	0.00	0.00	0.00	0.00	684438495.00	898343090.00	955832232.00
Perillyl alcohol	536-59-4	Terpenoids	31.51	0.00	0.00	0.00	0.00	0.00	0.00	0.00	0.00	0.00	0.00	0.00	0.00	2226637404.00	0.00	1989882941.00	0.00	0.00	0.00
Cinnamaldeh	1437	Aldehyde	32.03	0.00	0.00	0.00	0.00	0.00	0.00	9832414.	12671660	8968064.	0.00	0.00	0.00	68524867	9032541.	1193734.	21889559	0.00	25835688

yde	1-10-9									00	.00	00				.00	00	00	.00		.00
(1,3-Dioxaindan-5-ylmethyl)(methyl)amine	15205-27-3	Others	33.08	69384141.00	0.00	123865296.00	0.00	0.00	0.00	174605757.00	191074503.00	0.00	0.00	0.00	0.00	0.00	176431687.00	9933079.00	0.00	0.00	0.00
Spathulenol	6750-60-3	Terpenoids	33.78	0.00	0.00	0.00	8980398708.00	5815587722.00	4709919933.00	0.00	0.00	0.00	279058824.00	0.00	651374345.00	0.00	0.00	0.00	522006340.00	398227181.00	0.00
Carvacrol	499-75-2	Terpenoids	35.35	0.00	0.00	0.00	0.00	0.00	0.00	0.00	0.00	0.00	0.00	0.00	0.00	0.00	0.00	0.00	111179633.00	106623056.00	116367775.00
Alpha-cadinol, (-)-	481-34-5	Terpenoids	35.76	0.00	0.00	0.00	0.00	0.00	0.00	0.00	0.00	0.00	225759151.00	101631344.00	1807888236.00	0.00	0.00	0.00	0.00	0.00	0.00
Methyl palmitoleate	1120-25-8	Carboxylic acids	35.97	2455775641.00	4356206164.00	3031603413.00	0.00	0.00	0.00	0.00	0.00	0.00	103796729.00	65774786.00	0.00	3842847141.00	164260480.00	0.00	0.00	0.00	0.00
11,11-Dimethyl-4,8-dimethylenebicyclo[7.2.0]undecan-3-ol	79580-01-1	Alcohols	36.92	0.00	0.00	0.00	0.00	0.00	0.00	0.00	0.00	0.00	206760110.00	0.00	258201289.00	0.00	0.00	0.00	0.00	0.00	0.00
Shyobunol	35727-45-8	Terpenoids	37.40	0.00	0.00	0.00	314862723.00	161811376.00	156195616.00	0.00	0.00	0.00	0.00	0.00	0.00	0.00	0.00	0.00	0.00	0.00	0.00
(1R,7S,E)-7-Isopropyl-4,10-dimethylenecyclodec-5-eno	81968-62-9	Terpenoids	38.01	0.00	0.00	0.00	889290396.00	748591180.00	392592118.00	0.00	0.00	0.00	0.00	0.00	0.00	0.00	621345940.00	376939870.00	0.00	0.00	0.00

l																					
Methyl elaidate	1937-62-8	Carboxylic acids	39.43	0.00	296697653.00	365474808.00	0.00	0.00	0.00	0.00	0.00	0.00	0.00	0.00	0.00	0.00	0.00	0.00	0.00	0.00	0.00
Diisobutyl phthalate	84-69-5	Carboxylic acids	40.95	56595438.00	103765705.00	80082761.00	0.00	65646551.00	66496442.00	20076655.00	0.00	31862943.00	43565471.00	49407578.00	56879592.00	47439965.00	23564796.00	0.00	43365403.00	59963495.00	37329951.00
2,2-Bis(4-hydroxy-3-isopropylphenyl)propane	127-54-8	Phenols	42.70	0.00	0.00	0.00	0.00	0.00	0.00	0.00	584869177.00	464731595.00	757479832.00	830504480.00	0.00	639635568.00	840438346.00	622459450.00	0.00	608700883.00	800312605.00
Alanylglycine	687-69-4	Others	1.65	424853592.00	6093319249.00	6317459832.00	0.00	0.00	0.00	0.00	0.00	0.00	0.00	7442655258.00	7667130176.00	0.00	0.00	0.00	0.00	0.00	0.00
Pipamperone [usan:inn:ban]	1893-33-0	Ketones	4.06	0.00	0.00	0.00	0.00	0.00	0.00	0.00	4770194729.00	3175742134.00	0.00	0.00	0.00	0.00	0.00	0.00	0.00	0.00	0.00
Ethyl isovalerate	108-64-5	Carboxylic acids	7.46	0.00	0.00	0.00	0.00	0.00	0.00	943595712.00	1750877357.00	990084272.00	0.00	0.00	0.00	0.00	0.00	0.00	0.00	0.00	0.00
methyl valerate	624-24-8	Carboxylic acids	8.01	402544707.00	196592556.00	66508145.00	1592870272.00	3914546086.00	4101744908.00	123110273.00	3551088109.00	272762063.00	3473497881.00	338762109.00	532460591.00	45368390.00	564547987.00	88119138.00	4991137096.00	4895111102.00	5238388962.00
2-Butenal, 2-methyl-	1115-11-3	Aldehyde	8.09	0.00	0.00	0.00	0.00	0.00	0.00	0.00	23129923.00	44623877.00	1891282.00	0.00	124094177.00	61428790.00	0.00	60523225.00	0.00	0.00	0.00
2-Heptanone	110-43-0	Ketones	11.09	1984352074.00	91719041.00	191908732.00	56817871.00	39898588.00	56745517.00	17606826.00	21968786.00	97732981.00	0.00	0.00	0.00	153048714.00	258546308.00	186203840.00	1495617518.00	1848433276.00	2085915566.00
2-Amylfuran	3777-69-3	Others	12.67	47479020.00	22637424.00	30331464.00	50445593.00	115558004.00	73677091.00	7651443.00	0.00	6096823.00	81309679.00	55706094.00	110439075.00	41515491.00	0.00	272383692.00	1071004964.00	1090996051.00	1390893582.00
Beta-ocimene, (3z)-	3338-55-4	Terpenoids	13.29	0.00	0.00	0.00	278981000000.00	79298002110.00	0.00	513688924.00	457867491.00	0.00	4894823060.00	6128581739.00	1255947992.00	0.00	0.00	0.00	0.00	1313016296.00	2529649364.00
m-Cymene	535-	Terpenoi	13.76	0.00	0.00	0.00	0.00	16945180	15432759	0.00	0.00	0.00	67278299	0.00	42417764	52216184	74276038	55055592	16956688	19510497	22430117

	77-3	ds						693.00	892.00				70.00		63.00	396.00	19.00	329.00	168.00	602.00	027.00
2-Heptenal, (*Z*)-	57266-86-1	Aldehyde	15.37	0.00	0.00	0.00	140306400.00	124527303.00	175896692.00	243908740.00	631792991.00	270900690.00	54030398.00	0.00	71329421.00	0.00	113093251.00	287455005.00	0.00	0.00	0.00
Alpha-campholenAldehyde	4501-58-0	Aldehyde	19.89	0.00	0.00	0.00	0.00	0.00	0.00	0.00	0.00	0.00	0.00	0.00	0.00	4356726603.00	0.00	4157775080.00	0.00	10170863248.00	19643155238.00
Nopinone	38651-65-9	Terpenoids	22.12	0.00	0.00	0.00	0.00	0.00	0.00	0.00	0.00	0.00	0.00	0.00	0.00	5194048841.00	0.00	4599906250.00	0.00	0.00	0.00
2-Methoxy ethyl acetate	3938-96-3	Carboxylic acids	22.23	0.00	0.00	0.00	0.00	0.00	0.00	0.00	79441044.00	6376344.00	0.00	0.00	0.00	0.00	0.00	0.00	0.00	0.00	0.00
Beta-elemene	33880-83-0	Terpenoids	22.44	0.00	0.00	0.00	0.00	0.00	0.00	0.00	0.00	0.00	0.00	0.00	0.00	11973519361.00	7731530719.00	0.00	0.00	0.00	0.00
1,4-Dimethyl (2r)-2-hydroxybutanedioate	70681-41-3	Carboxylic acids	23.16	0.00	0.00	0.00	0.00	0.00	0.00	0.00	0.00	0.00	163134014.00	91729287.00	0.00	0.00	0.00	0.00	0.00	0.00	0.00
cis-Muurola-4(15),5-diene	157477-72-0	Terpenoids	25.99	0.00	0.00	0.00	262385252.00	170182009.00	156605114.00	0.00	0.00	0.00	0.00	0.00	0.00	4361465870.00	6250054877.00	1530543737.00	0.00	0.00	0.00
Methyl benzoate	93-58-3	Carboxylic acids	23.12	419385652.00	0.00	309117483.00	566591360.00	764227064.00	863320641.00	16066780.00	7751204.00	4360902229.00	0.00	0.00	0.00	0.00	316086998.00	0.00	0.00	0.00	0.00
Ehyl benzoate	93-89-0	Carboxylic acids	24.20	0.00	0.00	0.00	0.00	0.00	0.00	1482181.00	12800592.00	2921717397.00	0.00	0.00	0.00	0.00	0.00	0.00	0.00	0.00	0.00
Naphthalene, 1,2,3,4,4a,7-h	16728-99-	Hydrocarbons	26.82	538565863.00	590323101.00	0.00	6076759491.00	3033153905.00	3197617001.00	0.00	0.00	0.00	0.00	0.00	0.00	5440731069.00	4177607056.00	3955953794.00	0.00	0.00	0.00

exahydro-1,6-dimethyl-4-(1-methylethyl)-	7																				
Carveol, trans-(+/-)-	1197-07-5	Terpenoids	28.04	0.00	0.00	0.00	0.00	0.00	0.00	0.00	0.00	0.00	0.00	0.00	0.00	0.00	0.00	0.00	3617324614.00	6175399419.00	9682931064.00
Nerylacetone	3879-26-3	Ketones	28.41	0.00	0.00	0.00	0.00	0.00	0.00	0.00	0.00	0.00	641914232.00	132003161.00	0.00	0.00	0.00	0.00	0.00	0.00	0.00
o-Methoxyphenol	90-05-1	Phenols	28.43	0.00	0.00	0.00	0.00	0.00	0.00	40136121.00	32257368.00	87225546.00	0.00	0.00	0.00	0.00	0.00	0.00	0.00	0.00	0.00
2-Phenylpropanal	93-53-8	Aldehyde	28.95	0.00	0.00	0.00	0.00	0.00	0.00	2755382.00	4606234.00	0.00	283273425.00	0.00	626218395.00	0.00	0.00	0.00	0.00	0.00	0.00
2-Phenyl-2-butenal	4411-89-6	Hydrocarbons	29.90	25201217.00	46171299.00	51688958.00	14597029.00	5835221.00	1709110.00	75657223.00	157087022.00	86123008.00	0.00	0.00	0.00	135461025.00	0.00	535954.00	0.00	0.00	0.00
2,3-Dihydro-1H-indene-4-carbaldehyde	51932-70-8	Aldehyde	30.93	76766707.00	122065181.00	118040112.00	60367690.00	855046267.00	101964178.00	21378429.00	20285264.00	41858451.00	0.00	0.00	0.00	4868619743.00	16234750797.00	5781553968.00	703811415.00	1075807567.00	782880204.00
Phenol	108-95-2	Phenols	31.40	75235019.00	154398034.00	96485532.00	0.00	0.00	0.00	52398515.00	44598297.00	98510504.00	0.00	0.00	0.00	0.00	0.00	0.00	0.00	0.00	0.00
2,4-Di-tert-butylphenol	96-76-4	Phenols	37.12	0.00	0.00	0.00	15028080.00	39455672.00	21162299.00	0.00	0.00	0.00	0.00	0.00	0.00	0.00	0.00	0.00	16634811.00	20708120.00	0.00
Methyl linoleate	112-63-0	Carboxylic acids	40.23	431991654.00	500944595.00	1269717229.00	0.00	0.00	0.00	0.00	0.00	0.00	0.00	0.00	0.00	595911244.00	324552957.00	0.00	136314559.00	224794735.00	185849531.00
Linolenic acid, methyl ester	301-00-8	Carboxylic acids	41.29	233296960.00	271942002.00	409237196.00	0.00	73093048.00	185509079.00	0.00	0.00	0.00	165033778.00	0.00	50636112.00	348229746.00	225093508.00	0.00	91663181.00	185822829.00	150829770.00
Phosphoryleth	1071	Others	1.65	0.00	0.00	0.00	62880069	70289234	67127099	0.00	0.00	0.00	0.00	0.00	0.00	0.00	62008945	62628107	0.00	0.00	0.00

anolamine	-23-4						42.00	62.00	47.00								44.00	86.00			
Carbamylhydrazine	57-56-7	Carboxylic acids	5.96	0.00	0.00	0.00	0.00	0.00	0.00	0.00	0.00	0.00	0.00	0.00	0.00	11020170465.00	9703513741.00	7919560220.00	0.00	0.00	0.00
Methyl butenol	115-18-4	Alcohols	6.76	0.00	0.00	0.00	76084692.00	45998883.00	1182503946.00	0.00	0.00	0.00	6054675602.00	4651123083.00	2641641907.00	84162369.00	0.00	133080080.00	220538287.00	127040727.00	71945030.00
Bicyclo[2.2.1]heptane, 2,2-dimethyl-3-methylene-, (1S)-	1422353.00	Terpenoids	7.19	0.00	0.00	0.00	0.00	649183551.00	492699857.00	0.00	0.00	0.00	0.00	3078413670.00	1738516207.00	0.00	0.00	0.00	0.00	0.00	0.00
(-)-Beta-pinene	18172-67-3	Terpenoids	8.47	159533948814.00	128023000000.00	0.00	0.00	0.00	0.00	17356666637.00	2594882508.00	2472163785.00	18025381187.00	11431979630.00	5659235804.00	411110651048.00	413922000000.00	0.00	7025939946.00	9606813067.00	9337154807.00
1-Isopropyl-4-methylenebicyclo[3.1.0]hex-2-ene	36262-09-6	Terpenoids	9.09	0.00	0.00	0.00	0.00	0.00	0.00	0.00	0.00	0.00	0.00	0.00	0.00	0.00	0.00	0.00	1165461461.00	2763993875.00	4413153833.00
Dehydro-1,8-cineole	92760-25-3	Terpenoids	11.35	521280690.00	186707562.00	191398912.00	0.00	0.00	0.00	258840247.00	375566802.00	148001378.00	2575940231.00	0.00	1297298394.00	13074224.00	41371157.00	87650788.00	0.00	0.00	0.00
1,5,8-p-Menthatriene	21195-59-5	Terpenoids	18.17	0.00	0.00	0.00	0.00	0.00	0.00	125713171.00	104341987.00	41152929.00	0.00	0.00	0.00	0.00	0.00	0.00	0.00	0.00	0.00
Isobutenylbenzene	768-49-0	Hydrocarbons	14.48	0.00	0.00	0.00	0.00	0.00	0.00	0.00	0.00	0.00	0.00	0.00	0.00	0.00	0.00	0.00	2041400762.00	2069120690.00	0.00
3-methyl-2-(3	1518	Others	17.61	0.00	0.00	0.00	14773169	10797413	13104642	50198433	24803336	11722582	0.00	0.00	0.00	12329806	0.00	20552329	0.00	0.00	0.00

-Methylbut-2-enyl)-furan	6-51-3						60.00	8.00	8.00	.00	6.00	.00				09.00		34.00			
linalool oxide, (z)-	5989-33-3	Alcohols	18.80	0.00	0.00	0.00	0.00	0.00	0.00	1135246733.00	654720917.00	749215248.00	20866715958.00	25234642072.00	23321105124.00	0.00	0.00	0.00	2375777117.00	1705236631.00	1711710302.00
(+)-Cyclosativene	22469-52-9	Terpenoids	19.89	0.00	0.00	0.00	0.00	0.00	0.00	0.00	0.00	0.00	0.00	0.00	0.00	0.00	0.00	0.00	1770343355.00	0.00	267651607.00
Fenchol	1632-73-1	Terpenoids	22.35	0.00	0.00	0.00	0.00	0.00	0.00	0.00	207118384.00	254932159.00	318805460.00	328628625.00	319657187.00	313638867.00	10980040.00	596224107.00	37869838.00	0.00	1751926831.00
4-Terpineol	562-74-3	Terpenoids	22.80	19216826197.00	12901182384.00	12628603446.00	0.00	0.00	0.00	15235556251.00	14417451062.00	5575097579.00	3358368936.00	3098444584.00	1548538276.00	78253181814.00	41818655853.00	35221615831.00	4052930877.00	4620740932.00	8773283034.00
N-Tetradecylamine	2016-42-4	Others	11.32	0.00	0.00	0.00	13522857709.00	0.00	2209949390.00	0.00	0.00	0.00	0.00	0.00	0.00	0.00	0.00	0.00	0.00	0.00	0.00
4-Isopropyl-2-cyclohexenone	500-02-7	Ketones	24.36	0.00	0.00	0.00	0.00	0.00	0.00	0.00	0.00	0.00	0.00	0.00	0.00	0.00	0.00	0.00	0.00	485296345.00	1723583252.00
Acetic acid--(1r,2r,5r)-4,6,6-trimethylbicyclo[3.1.1]hept-3-en-2-ol (1/1)	29135-27-1	Carboxylic acids	24.64	0.00	0.00	0.00	0.00	0.00	0.00	0.00	0.00	0.00	0.00	0.00	0.00	0.00	0.00	0.00	14654936994.00	29901362125.00	46886489626.00
rac-2-Despiperidyl-2-amino Repaglinide	874908-11-9	Carboxylic acids	18.15	0.00	0.00	0.00	0.00	0.00	0.00	0.00	0.00	0.00	9227220426.00	398707290.00	0.00	0.00	0.00	0.00	0.00	0.00	0.00
(-)-Carvone	6485	Terpenoi	25.78	0.00	0.00	0.00	0.00	0.00	0.00	0.00	0.00	0.00	0.00	0.00	0.00	0.00	0.00	0.00	0.00	43951388	61012334

	-40-1	ds																		48.00	82.00
CuminAldehyde	122-03-2	Terpenoids	26.75	0.00	0.00	0.00	0.00	0.00	0.00	0.00	0.00	0.00	0.00	0.00	0.00	272934890.00	0.00	512922492.00	401511178.00	450859916.00	495111894.00
(1r,4s,6s)-1,3,3-Trimethyl-2-oxabicyclo[2.2.2]octan-6-yl acetate	57709-95-2	Carboxylic acids	28.59	0.00	0.00	0.00	3679348339.00	0.00	788487251.00	0.00	0.00	0.00	0.00	0.00	0.00	0.00	0.00	0.00	0.00	1569564930.00	1666669545.00
Talamenene, trans-(+/-)-	73209-42-4	Terpenoids	27.90	0.00	122361443.00	106720265.00	3401008984.00	2743536625.00	2546825137.00	0.00	0.00	0.00	0.00	0.00	0.00	1413514424.00	0.00	1240605727.00	0.00	0.00	0.00
Cubenol	21284-22-0	Terpenoids	34.79	0.00	134695488.00	189336167.00	0.00	0.00	0.00	0.00	0.00	0.00	0.00	0.00	0.00	0.00	0.00	0.00	0.00	0.00	0.00
Elemol	639-99-6	Terpenoids	32.96	703302008.00	0.00	3270806157.00	0.00	0.00	0.00	0.00	0.00	0.00	0.00	0.00	0.00	31482452597.00	17206470423.00	3040824286.00	0.00	0.00	0.00
Guaiol	489-86-1	Terpenoids	33.15	1544719300.00	1846076130.00	3220164137.00	0.00	0.00	0.00	0.00	0.00	0.00	0.00	0.00	0.00	4410923397.00	0.00	815941465.00	0.00	0.00	0.00
Cumic alcohol	536-60-7	Terpenoids	33.33	0.00	0.00	0.00	0.00	0.00	0.00	0.00	0.00	0.00	0.00	0.00	0.00	631500688.00	0.00	313098003.00	229952045.00	107403754.00	190754734.00
Beta-eudesmol	473-15-4	Terpenoids	35.71	387821802.00	167014945.00	438126653.00	0.00	0.00	0.00	0.00	0.00	0.00	0.00	0.00	0.00	22383907242.00	14890543227.00	3187992132.00	0.00	0.00	0.00
(4aS,8R)-4a,8-Dimethyl-4,4a,5,6,7,8-hexahydronaphthal	69460-62-4	Ketones	34.49	0.00	0.00	0.00	0.00	0.00	0.00	0.00	0.00	0.00	0.00	0.00	0.00	0.00	0.00	0.00	0.00	57651010.00	68232732.00

en-2(3H)-one																					
N-Acetylcysteine	616-91-1	Others	18.39	0.00	0.00	0.00	0.00	0.00	0.00	0.00	0.00	0.00	0.00	0.00	0.00	6788304561.00	253575748.00	0.00	0.00	0.00	0.00
1,2-cyclohexanediol, 1-methyl-4-(1-methylethenyl)-	1946-00-5	Terpenoids	36.49	0.00	0.00	0.00	0.00	0.00	0.00	0.00	0.00	0.00	0.00	0.00	0.00	420038519.00	0.00	157245529.00	487968264.00	653500199.00	447299817.00
Alpha-terpinene	99-86-5	Terpenoids	10.90	946460542.00	356919430.00	738071739.00	3568411859.00	3160597770.00	1686481737.00	0.00	0.00	0.00	890791533.00	0.00	99933745.00	5143659587.00	2638403988.00	0.00	0.00	177438691.00	439336043.00
Thujone	546-80-5	Terpenoids	18.60	0.00	0.00	0.00	0.00	0.00	0.00	0.00	0.00	0.00	0.00	112198309.00	92441198.00	0.00	0.00	0.00	856621945.00	0.00	1000035950.00
Isopinocamphone	15358-88-0	Terpenoids	21.29	0.00	0.00	0.00	456105153.00	138769538.00	146682523.00	0.00	0.00	0.00	0.00	0.00	0.00	10656767356.00	0.00	6298990803.00	0.00	0.00	0.00
2-Cyclohexen-1-ol, 1-methyl-4-(1-methylethyl)-, (1r,4r)-rel-	29803-82-5	Alcohols	23.45	0.00	0.00	0.00	0.00	0.00	0.00	582101271.00	605423126.00	0.00	0.00	0.00	0.00	2504083397.00	0.00	2178436072.00	0.00	0.00	0.00
Isonicotinic acid diethylamide	530-40-5	Carboxylic acids	11.15	1984352074.00	1302995115.00	1312491811.00	0.00	0.00	0.00	0.00	0.00	0.00	0.00	0.00	0.00	0.00	0.00	0.00	0.00	0.00	0.00
1-Octen-3-ol	3391-86-4	Alcohols	19.04	0.00	0.00	0.00	0.00	0.00	0.00	2289484095.00	2457568978.00	0.00	276748467.00	9875983.00	262723001.00	1663094095.00	0.00	1823981450.00	3741862418.00	6458903448.00	9140350965.00
4a(2H)-Napht	7336	Terpenoi	32.68	0.00	0.00	0.00	40084703	81277542	56002627	0.00	0.00	0.00	0.00	0.00	0.00	70002227	81681125	77043411	0.00	91548535	68622656

halenol, 1,3,4,5,6,8a-hexahydro-4,7-dimethyl-1-(1-methylethyl)-, (1S,4S,4aS,8aR)-	5-77-2	ds					2.00	8.00	3.00							2.00	4.00	9.00		.00	.00
Methyl propionate	554-12-1	Carboxylic acids	3.41	0.00	0.00	0.00	0.00	0.00	0.00	0.00	0.00	0.00	7541456540.00	0.00	85012868078.00	0.00	0.00	0.00	92236187390.00	78348190266.00	0.00
Methyl vinyl ketone	78-94-4	Ketones	4.06	0.00	0.00	0.00	707455412.00	409376456.00	535740035.00	0.00	0.00	0.00	0.00	318690423.00	268891195.00	1242383750.00	0.00	1394408115.00	0.00	0.00	0.00
Trans-piperitol	16721-39-4	Terpenoids	26.13	0.00	0.00	0.00	94890503.00	86381333.00	0.00	0.00	0.00	0.00	0.00	0.00	0.00	0.00	0.00	0.00	0.00	0.00	0.00
Myrtenol, (-)-	19894-97-4	Terpenoids	27.15	0.00	0.00	0.00	0.00	0.00	0.00	0.00	0.00	0.00	0.00	0.00	0.00	0.00	0.00	0.00	681419841.00	0.00	2262862250.00
Ledol	577-27-5	Terpenoids	31.99	0.00	0.00	0.00	362513040.00	232203866.00	202454802.00	61070879.00	70040061.00	0.00	0.00	0.00	0.00	0.00	0.00	0.00	0.00	0.00	0.00
2-Ethylfuran	3208-16-0	Others	4.21	0.00	0.00	0.00	1384148044.00	1435250343.00	1528907391.00	0.00	0.00	0.00	4202820774.00	2839002207.00	3561813321.00	0.00	0.00	0.00	2652006355.00	1965688028.00	1799866686.00
Methyl n-butyrate	623-42-7	Carboxylic acids	4.97	929012302.00	64900334.00	140432020.00	274871327.00	452945205.00	639931273.00	45564931.00	15272954.00	143546668.00	552750816.00	98954369.00	146844397.00	49425088.00	325443272.00	21453139.00	511299861.00	447032090.00	234941878.00
4,7-Dimethyl undecane	17301-32-	Hydrocarbons	6.50	0.00	0.00	0.00	0.00	0.00	0.00	0.00	0.00	0.00	0.00	11191009.00	24792852.00	0.00	0.00	0.00	0.00	0.00	0.00

	5																				
Undecane, 3-methyl-	1002-43-3	Hydrocarbons	10.75	0.00	0.00	0.00	956637.00	10692850.00	266211726.00	30555706.00	0.00	9693115.00	0.00	335632121.00	213011498.00	0.00	0.00	0.00	0.00	0.00	0.00
Methyl 3-hexenoate, (3e)-	13894-61-6	Carboxylic acids	13.54	4504501.00	42201357.00	0.00	671516256.00	2636835926.00	0.00	0.00	0.00	0.00	14450309482.00	0.00	4646020561.00	0.00	0.00	0.00	0.00	0.00	0.00
4-MethyltetrAdecane	25117-24-2	Hydrocarbons	16.62	0.00	0.00	0.00	0.00	2253980.00	5940257.00	0.00	0.00	0.00	0.00	0.00	0.00	0.00	0.00	0.00	128187174.00	0.00	39284208.00
N-Methylpropanamide	1187-58-2	Carboxylic acids	19.93	0.00	0.00	0.00	0.00	0.00	0.00	0.00	0.00	0.00	9484932.00	62435770.00	345662.00	0.00	0.00	0.00	0.00	0.00	0.00
Methyl decanoate	110-42-9	Carboxylic acids	22.62	74008007622.00	146179000000.00	109429220981.00	15781791550.00	9377224888.00	10248684040.00	625772378.00	0.00	375061086.00	19295143593.00	51397322222.00	3468099435.00	20748133295.00	17520720829.00	2031010426.00	23025944883.00	15732068181.00	6909640685.00
Borneol	507-70-0	Terpenoids	25.12	0.00	0.00	0.00	0.00	0.00	0.00	36696111914.00	0.00	22687974345.00	0.00	0.00	0.00	0.00	0.00	0.00	0.00	0.00	0.00
(*E*)-Dec-2-enyl acetate	2497-23-6	Carboxylic acids	25.90	1769466243.00	8803387868.00	2179418243.00	0.00	0.00	0.00	0.00	0.00	0.00	0.00	0.00	0.00	1183690092.00	0.00	4823430609.00	2974250824.00	1820708658.00	2185745322.00
Hexanoic acid	142-62-1	Carboxylic acids	28.44	0.00	0.00	0.00	3294777554.00	6335793269.00	4755626427.00	0.00	0.00	0.00	9406783977.00	1216229522.00	9171570050.00	0.00	0.00	0.00	30959904395.00	18364096689.00	29252514520.00
trans-beta-ionone	79-77-6	Terpenoids	30.16	408200207.00	145964039.00	61425057.00	624513364.00	0.00	547775351.00	0.00	0.00	0.00	466801815.00	235114360.00	87163332.00	1233421519.00	568255128.00	870254371.00	1521163859.00	0.00	1453230186.00
Benzothiazole	95-16-9	Others	30.42	0.00	0.00	0.00	221579043.00	0.00	35376078.00	0.00	0.00	0.00	66494253.00	28701025.00	20218680.00	0.00	0.00	0.00	19111087.00	9138290.00	0.00
Hexadecanal	629-80-1	Aldehyde	31.93	0.00	0.00	0.00	0.00	0.00	0.00	0.00	0.00	0.00	0.00	0.00	0.00	1425234495.00	0.00	391307715.00	0.00	0.00	0.00
Ethyl	628-	Carboxyli	36.18	18832088	35159726	20508277	26362239	14698711	17081745	55696360	40055384	37420358	72006181	68049743	0.00	28972143	16426048	0.00	17841906	15925895	15577202

palmitate	97-7	c acids		7.00	9.00	6.00	3.00	5.00	9.00	.00	.00	.00	.00	.00		8.00	0.00		7.00	1.00	5.00
Sobrerol	498-71-5	Terpenoids	37.31	0.00	0.00	0.00	0.00	0.00	0.00	0.00	0.00	0.00	0.00	0.00	0.00	0.00	0.00	0.00	1315447579.00	2009160173.00	0.00
Methyl Margarate	1731-92-6	Carboxylic acids	37.33	62914680.00	92286485.00	204742705.00	0.00	32358929.00	53200975.00	0.00	0.00	0.00	0.00	0.00	0.00	354512528.00	53106957.00	0.00	0.00	0.00	0.00
Dihydroactinidiolide	17092-92-1	Carboxylic acids	37.66	141889686.00	0.00	38794884.00	353011029.00	547951424.00	451401397.00	33943392.00	0.00	17008519.00	221993115.00	220062758.00	207493492.00	538103309.00	0.00	167263058.00	495450284.00	370252164.00	2508210837.00
Vanillin	121-33-5	Aldehyde	41.23	0.00	0.00	0.00	267179736.00	0.00	29951154.00	0.00	0.00	0.00	0.00	0.00	0.00	0.00	0.00	0.00	0.00	0.00	0.00
N,N-Diphenylamine	122-39-4	Others	41.79	0.00	0.00	0.00	47694735.00	38823484.00	41042999.00	0.00	0.00	0.00	29570579.00	39358850.00	45919439.00	0.00	0.00	0.00	0.00	47566000.00	53838686.00
2-Methylpropanal	78-84-2	Aldehyde	2.47	134582898.00	116076283.00	88154555.00	222435416.00	420364614.00	281590591.00	624724062.00	0.00	633868177.00	5814578546.00	1780848340.00	2329940971.00	0.00	0.00	0.00	0.00	0.00	0.00
2-Propyl-1-heptanol	10042-59-8	Alcohols	16.49	35266900.00	8359720.00	0.00	0.00	0.00	0.00	0.00	0.00	0.00	0.00	0.00	0.00	0.00	0.00	0.00	0.00	0.00	0.00
Methyl 3-hexenoate, (3z)-	13894-62-7	Carboxylic acids	13.54	0.00	0.00	0.00	39129270002.00	82848159775.00	80056201427.00	8240928.00	3438604.00	0.00	0.00	87488251472.00	105884666729.00	0.00	0.00	0.00	8424984039.00	5932467399.00	0.00
1-Hexanol	111-27-3	Alcohols	16.45	0.00	0.00	0.00	0.00	234889645.00	447987865.00	52530538.00	58480809.00	88024055.00	14815116620.00	7362152149.00	8780058051.00	0.00	0.00	0.00	5784294702.00	6811325463.00	4217749236.00
2-Methoxyethanol	109-86-4	Alcohols	17.70	0.00	0.00	0.00	0.00	0.00	0.00	0.00	0.00	0.00	0.00	0.00	0.00	0.00	0.00	0.00	0.00	560243142.00	360979067.00
p-Alpha-dimethyl styrene	1195-32-0	Hydrocarbons	18.49	0.00	1045746680.00	1127454214.00	0.00	0.00	0.00	0.00	0.00	0.00	496222454.00	0.00	48464892.00	0.00	0.00	0.00	0.00	0.00	0.00

4-Thujanol, cis-(+/-)-	15537-55-0	Terpenoids	19.44	4982401185.00	2531055077.00	3556875892.00	15742544580.00	5387193314.00	6453953228.00	1277484971.00	1587712433.00	1690481202.00	0.00	178135236.00	113212851.00	28088996835.00	7321516250.00	15247707885.00	1565061631.00	1596277736.00	1683756475.00
p-Bromochlorobenzene	106-39-8	Others	22.09	0.00	0.00	0.00	1202084548.00	802386098.00	566869496.00	0.00	0.00	0.00	0.00	0.00	0.00	0.00	0.00	0.00	0.00	26077678.00	39920399.00
2-Decen-1-ol, (*Z*)-	4194-71-2	Alcohols	27.69	0.00	0.00	0.00	0.00	0.00	0.00	0.00	0.00	0.00	0.00	0.00	0.00	0.00	0.00	0.00	1011212304.00	711932340.00	0.00
6-Methyl-6-(5-methylfuran-2-yl)heptan-2-one	50464-95-4	Ketones	31.22	46167229.00	1431200602.00	0.00	0.00	0.00	0.00	0.00	0.00	0.00	0.00	0.00	0.00	0.00	0.00	0.00	0.00	0.00	0.00
Methyl cinnamate	103-26-4	Carboxylic acids	32.79	0.00	0.00	0.00	0.00	0.00	0.00	0.00	0.00	0.00	0.00	509095172.00	1222736086.00	0.00	0.00	0.00	2007117755.00	1839057709.00	0.00
N-Octane	111-65-9	Hydrocarbons	2.39	67174764.00	0.00	12312037.00	0.00	0.00	0.00	0.00	0.00	0.00	0.00	0.00	0.00	2880064.00	34480429.00	6467654.00	110190491.00	88093534.00	67283323.00
3-Methylbutanal	590-86-3	Aldehyde	3.56	31387097.00	2144570.00	7158584.00	15585310.00	28105572.00	21460588.00	5127283.00	19952924.00	308319255.00	4239205171.00	254544438.00	387051697.00	0.00	0.00	0.00	5276703.00	40323011.00	124654466.00
2-Hexenal	505-57-7	Aldehyde	12.16	56254716.00	31428473.00	6895479.00	371067903.00	3445145628.00	3749645664.00	0.00	0.00	0.00	85616519997.00	74180946281.00	114230551499.00	0.00	0.00	0.00	6205649267.00	7232216291.00	5751269129.00
Hexadecylamine	143-27-1	Others	12.58	54909682072.00	1968209057.00	0.00	0.00	54458409.00	442692241.00	0.00	0.00	0.00	0.00	0.00	0.00	0.00	0.00	0.00	0.00	0.00	0.00
Beta-bourbonene	5208-59-3	Terpenoids	20.57	0.00	0.00	0.00	907541090.00	2426607524.00	9980978522.00	0.00	0.00	0.00	0.00	0.00	0.00	0.00	0.00	0.00	0.00	0.00	0.00
(-)-Bornyl acetate	5655-61-8	Terpenoids	22.21	0.00	0.00	0.00	1039058680.00	125655362.00	0.00	0.00	5465442136.00	1331280047.00	0.00	0.00	0.00	0.00	0.00	0.00	0.00	0.00	0.00
(R)-(+)-alpha-	7785	Terpenoi	25.03	0.00	0.00	0.00	0.00	0.00	0.00	0.00	0.00	0.00	44178455	43029204	0.00	0.00	0.00	0.00	0.00	0.00	0.00

Terpineol	-53-7	ds											17.00	37.00							
5-Azulenemethanol, 1,2,3,4,5,6,7,8-octahydro-alpha,alpha,3,8-tetramethyl-	13822-35-0	Alcohols	33.15	0.00	0.00	0.00	3087156405.00	700014218.00	950708927.00	0.00	0.00	0.00	0.00	0.00	0.00	0.00	0.00	0.00	0.00	0.00	0.00
Trimethylamine	75-50-3	Others	2.32	0.00	0.00	0.00	612871.00	0.00	0.00	0.00	0.00	0.00	0.00	0.00	0.00	0.00	0.00	0.00	0.00	0.00	0.00
ButyrAldehyde	123-72-8	Aldehyde	3.02	4179796.00	17028838.00	2092719.00	24363746.00	28835225.00	40121799.00	0.00	0.00	0.00	274154826.00	280087414.00	330976023.00	4806716.00	12122264.00	4563648.00	162118661.00	127541374.00	208554376.00
Methyl 2-methylbutyrate	868-57-5	Carboxylic acids	5.63	38819619.00	12623603.00	22365993.00	449299969.00	512411246.00	674221502.00	103997892.00	244291935.00	372278965.00	1738581468.00	77399905.00	63673424.00	88849730.00	260016289.00	27899107.00	231855059.00	244717939.00	322753695.00
Sec-butyl alcohol	78-92-2	Alcohols	6.30	9710785.00	0.00	12221137.00	4779302.00	51955173.00	0.00	86472932.00	0.00	41869259.00	156903287.00	44229547.00	0.00	6251531.00	617792920.00	950302.00	0.00	0.00	0.00
Trans-2-pentenal	1576-87-0	Aldehyde	9.27	4221004.00	5342771.00	0.00	1375214915.00	780792435.00	707095215.00	0.00	0.00	0.00	776540560.00	1022777265.00	1229448352.00	0.00	0.00	0.00	1341765134.00	830130527.00	1123295038.00
Methyl pyruvate	600-22-6	Carboxylic acids	12.76	0.00	0.00	0.00	0.00	0.00	0.00	53254337.00	0.00	35372211.00	0.00	2675496366.00	747992882.00	0.00	0.00	0.00	48874916.00	27833184.00	42786793.00
Methyl 3-Hexenoate	2396-78-3	Carboxylic acids	13.52	0.00	0.00	0.00	0.00	0.00	0.00	0.00	0.00	0.00	0.00	0.00	0.00	0.00	0.00	0.00	170147282.00	116915689523.00	109014399352.00
2,4-Heptadienal, (*E,E*)-	4313/3/5	Aldehyde	19.26	629313860.00	760943007.00	0.00	0.00	0.00	0.00	0.00	0.00	0.00	481453277.00	0.00	780724681.00	0.00	0.00	0.00	1380382853.00	740301397.00	1396347417.00
Gamma-amorphene	6980-46-7	Terpenoids	22.11	0.00	0.00	0.00	3430170755.00	9550062859.00	9760747613.00	0.00	0.00	0.00	0.00	0.00	0.00	0.00	0.00	0.00	0.00	0.00	0.00

Phenylacetaldehyde	122-78-1	Aldehyde	23.54	0.00	0.00	0.00	0.00	0.00	0.00	0.00	214761536.00	117988495.00	0.00	0.00	0.00	0.00	0.00	0.00	0.00	0.00	0.00
2-Decenal, (2z)-	2497-25-8	Aldehyde	23.73	30064075572.00	176043000000.00	0.00	0.00	0.00	0.00	0.00	0.00	0.00	0.00	0.00	0.00	399816221512.00	13651315848.00	144080000000.00	0.00	0.00	0.00
Isovaleric acid	503-74-2	Carboxylic acids	24.60	0.00	0.00	0.00	863674518.00	1173789238.00	1031004371.00	0.00	0.00	0.00	0.00	0.00	0.00	0.00	0.00	0.00	0.00	0.00	0.00
4-Hexanolide	695-06-7	Carboxylic acids	24.98	0.00	0.00	0.00	0.00	6915429561.00	6822867412.00	0.00	0.00	0.00	932070528.00	738954762.00	676469585.00	0.00	0.00	0.00	2493004859.00	2051992263.00	2172562653.00
N-Pentanoic acid	109-52-4	Carboxylic acids	26.20	0.00	0.00	0.00	63692674.00	274671146.00	193027162.00	0.00	0.00	0.00	0.00	0.00	0.00	0.00	0.00	0.00	168599110.00	0.00	333373475.00
4-Oxohex-2-enal	20697-55-6	Aldehyde	26.24	0.00	0.00	0.00	0.00	0.00	0.00	0.00	0.00	0.00	4490097729.00	3165335549.00	2462459754.00	0.00	0.00	0.00	1802914783.00	1115855669.00	1375050630.00
Methyl salicylate	119-36-8	Carboxylic acids	26.61	0.00	0.00	0.00	353235478.00	334697751.00	220400447.00	0.00	0.00	0.00	2220678123.00	145051660.00	347128851.00	0.00	0.00	0.00	0.00	0.00	0.00
Octyl hexanoate	4887-30-3	Carboxylic acids	27.56	192512710.00	1999121336.00	4961907011.00	170246274.00	64700399.00	53831645.00	0.00	0.00	0.00	0.00	0.00	0.00	0.00	0.00	0.00	500108470.00	672039982.00	0.00
Alpha-ionone	127-41-3	Terpenoids	28.33	0.00	0.00	0.00	1696952450.00	1236979703.00	933034091.00	0.00	0.00	0.00	0.00	0.00	0.00	0.00	0.00	0.00	0.00	0.00	0.00
3-Buten-2-one, 4-(2,2,6-trimethyl-7-oxabicyclo(4.1.0)hept-1-yl)-	23267-57-4	Terpenoids	31.22	0.00	0.00	0.00	686496411.00	1568595570.00	1760647585.00	0.00	0.00	0.00	999296630.00	699023192.00	1081555151.00	0.00	0.00	0.00	3350921166.00	2180319622.00	2508963302.00
Cedrol	77-5	Terpenoi	33.68	0.00	0.00	0.00	0.00	60788621	38401640	0.00	0.00	0.00	0.00	2298885.	49580630	0.00	0.00	0.00	0.00	0.00	0.00

	3-2	ds						.00	.00					00	3.00						
Methyl 8-(2-furyl)octanoate	38199-50-7	Carboxylic acids	34.88	0.00	0.00	0.00	9413855.00	4626014.00	6288596.00	0.00	0.00	0.00	27966297.00	11231836.00	0.00	0.00	0.00	0.00	359166761.00	20519315.00	25278498.00
3-Ethyl-4-methyl-1h-pyrrole-2,5-dione	20189-42-8	Carboxylic acids	36.36	0.00	0.00	0.00	224818452.00	331224204.00	236109709.00	0.00	0.00	0.00	166444346.00	83083877.00	2581483831.00	0.00	0.00	0.00	239060851.00	169633426.00	373538804.00
Methyl cyclohexylamine	100-60-7	Others	11.69	0.00	0.00	0.00	0.00	0.00	0.00	0.00	0.00	0.00	0.00	0.00	0.00	0.00	0.00	0.00	136487.00	0.00	185888750.00
Methyl heptanoate	106-73-0	Carboxylic acids	14.41	0.00	0.00	0.00	5531555290.00	11197789444.00	13036707891.00	0.00	0.00	0.00	0.00	0.00	0.00	0.00	0.00	0.00	15741926910.00	16943203634.00	13705936837.00
4-Heptenoic acid, methyl ester, (*E*)-	54004-29-4	Carboxylic acids	15.74	0.00	0.00	0.00	8454906464.00	16218125185.00	21279678822.00	0.00	0.00	0.00	0.00	0.00	0.00	0.00	0.00	0.00	2836281384.00	8223173716.00	0.00
3,4-Dimethyl-2,4,6-octatriene	57396-75-5	Hydrocarbons	16.79	207064015.00	376937242.00	0.00	0.00	0.00	0.00	0.00	0.00	0.00	0.00	0.00	0.00	0.00	0.00	0.00	0.00	0.00	0.00
Ylangene	14912-44-8	Terpenoids	19.75	16014106267.00	4525903599.00	4637142634.00	8038118143.00	40531718873.00	43676430758.00	0.00	0.00	0.00	0.00	0.00	0.00	3111877825.00	0.00	8191573118.00	0.00	0.00	0.00
PerillAldehyde	2111-75-3	Terpenoids	26.85	0.00	0.00	0.00	0.00	0.00	0.00	0.00	0.00	0.00	0.00	0.00	0.00	5440731069.00	4177607056.00	3955953794.00	0.00	0.00	0.00
Melilotic acid	495-78-3	Carboxylic acids	43.02	0.00	0.00	0.00	427778696.00	0.00	231305213.00	0.00	0.00	0.00	0.00	0.00	0.00	0.00	0.00	0.00	0.00	0.00	0.00
2-Hexylfuran	3777	Others	15.64	19618694	43829831	60205760	45710740	13584167	14819780	0.00	0.00	0.00	0.00	0.00	0.00	43915887	42622512	12750708	0.00	0.00	0.00

	-70-6			0.00	0.00	4.00	1.00	04.00	77.00							71.00	16.00	77.00			
1,5,5,6-Tetramethyl-1,3-cyclohexadiene	514-94-3	Hydrocarbons	17.29	0.00	0.00	0.00	0.00	0.00	0.00	0.00	0.00	0.00	0.00	0.00	0.00	0.00	2610342436.00	5795074554.00	0.00	0.00	0.00
2-Propylfuran	4229-91-8	Others	19.25	0.00	0.00	0.00	1064986442.00	0.00	1062762497.00	0.00	0.00	0.00	0.00	0.00	0.00	0.00	0.00	0.00	0.00	0.00	0.00
Dihydro-alpha-ionone	31499-72-6	Terpenoids	27.60	0.00	0.00	0.00	53446909.00	19621577.00	17516090.00	0.00	0.00	0.00	0.00	0.00	0.00	0.00	0.00	0.00	0.00	0.00	0.00
Heptanoic acid	111-14-8	Carboxylic acids	30.78	0.00	0.00	0.00	106383506.00	83332195.00	0.00	0.00	200963482.00	375590151.00	0.00	0.00	0.00	0.00	0.00	0.00	270725391.00	0.00	270497179.00
2-Indolinone, 1,3,3-trimethyl-	20200-86-6	Ketones	32.83	0.00	0.00	0.00	0.00	58765216.00	66883933.00	825142451.00	595635089.00	0.00	1000535522.00	1104644866.00	387503860.00	0.00	0.00	0.00	287117607.00	251321278.00	0.00
Methyl t-butyl ether	1634-04-4	Ethers	1.94	0.00	0.00	0.00	0.00	0.00	0.00	0.00	0.00	0.00	416381809.00	1907819.00	18898267.00	0.00	0.00	0.00	4497539.00	8197294.00	0.00
2-MethylbutyrAldehyde	96-17-3	Aldehyde	3.49	0.00	0.00	0.00	32861343.00	34764804.00	23952058.00	0.00	0.00	0.00	4111774454.00	77296579.00	168450580.00	453052.00	0.00	879500.00	0.00	1322549.00	9479489.00
2-Heptanol	543-49-7	Alcohols	15.53	1834946839.00	687046956.00	730143679.00	2848243731.00	418074107.00	584819496.00	72661665.00	0.00	1108728132.00	0.00	0.00	0.00	1820638684.00	1162171554.00	334265942.00	0.00	0.00	0.00
4-Decenal, (4z)-	21662-09-9	Aldehyde	21.16	958039708.00	13227852283.00	9043243164.00	0.00	0.00	0.00	0.00	0.00	0.00	0.00	0.00	0.00	0.00	0.00	0.00	0.00	451509806.00	1203427398.00
Synephrine	1994/7/5	Others	21.64	0.00	0.00	0.00	0.00	0.00	0.00	0.00	0.00	0.00	3926313465.00	3538804024.00	0.00	0.00	0.00	0.00	0.00	0.00	0.00
Epicubenol	1991	Terpenoi	32.52	0.00	0.00	0.00	0.00	10215989	88728863	0.00	0.00	0.00	0.00	0.00	0.00	0.00	0.00	0.00	0.00	0.00	0.00

	2-67-5	ds						55.00	8.00												
Nonanoic acid	112-05-0	Carboxylic acids	34.90	0.00	0.00	0.00	0.00	0.00	0.00	0.00	0.00	0.00	0.00	0.00	0.00	0.00	0.00	0.00	191967636.00	267513103.00	331364074.00
7-Hexadecenoic acid, methyl ester, (7z)	56875-67-3	Carboxylic acids	35.97	0.00	0.00	0.00	0.00	0.00	0.00	0.00	0.00	0.00	0.00	0.00	0.00	0.00	1728484557.00	561546573.00	0.00	0.00	0.00
Decanoic acid	334-48-5	Carboxylic acids	36.83	95861883.00	0.00	280108250.00	0.00	0.00	0.00	0.00	0.00	0.00	0.00	0.00	0.00	0.00	0.00	0.00	0.00	0.00	0.00
Methyl 9,12-hexadecadienoate, (9z,12z)-	2462-80-8	Carboxylic acids	37.04	305423820.00	739628823.00	522415483.00	0.00	0.00	0.00	0.00	0.00	0.00	0.00	0.00	0.00	0.00	0.00	0.00	0.00	0.00	0.00
Methyl isopropyl ketone	563-80-4	Ketones	4.74	0.00	0.00	0.00	0.00	495631.00	196314.00	0.00	0.00	0.00	0.00	0.00	0.00	0.00	0.00	0.00	7456528.00	12729913.00	12780192.00
1-Methoxy-2-hydroxypropane	107-98-2	Alcohols	9.44	0.00	0.00	0.00	20699164.00	0.00	233768368.00	0.00	0.00	0.00	0.00	0.00	0.00	0.00	0.00	0.00	0.00	0.00	0.00
3-Hexenal	4440-65-7	Aldehyde	9.64	0.00	0.00	0.00	0.00	0.00	0.00	0.00	0.00	0.00	154786169.00	339918091.00	605274191.00	0.00	0.00	0.00	0.00	68593017.00	60249101.00
1,3,5-Trichlorobenzene	108-70-3	Hydrocarbons	23.51	0.00	0.00	0.00	0.00	0.00	0.00	0.00	0.00	0.00	0.00	0.00	0.00	68833631.00	0.00	10010680.00	0.00	0.00	0.00
Delta-cadinene	483-76-1	Terpenoids	26.28	5502647323.00	2928083727.00	3134809017.00	134949000000.00	92663902563.00	90530495582.00	6024191282.00	8899918352.00	6207485339.00	0.00	0.00	0.00	68772927704.00	64384514068.00	0.00	0.00	0.00	0.00

2-dodecenal, (2e)-	20407-84-5	Aldehyde	28.52	0.00	0.00	0.00	0.00	0.00	0.00	0.00	0.00	0.00	0.00	0.00	0.00	0.00	0.00	0.00	5528332509.00	4788917162.00	0.00
N-Butyrylho Moserine lactone	67605-85-0	Carboxylic acids	25.86	0.00	0.00	0.00	0.00	0.00	0.00	0.00	693623950.00	24262847.00	0.00	0.00	0.00	0.00	0.00	0.00	0.00	0.00	0.00
Farnesal	19317-11-4	Alcohols	36.32	0.00	0.00	0.00	33319037.00	0.00	236109709.00	0.00	0.00	0.00	0.00	0.00	0.00	0.00	0.00	0.00	0.00	0.00	0.00
2-Octyl acetate	2051-50-5	Carboxylic acids	16.59	0.00	0.00	0.00	0.00	0.00	0.00	0.00	21440685.00	307249199.00	0.00	0.00	0.00	0.00	0.00	0.00	0.00	0.00	0.00
1-Nonene	124-11-8	Hydrocarbons	4.01	1558645628.00	1514225995.00	450542517.00	0.00	0.00	0.00	0.00	0.00	0.00	0.00	0.00	0.00	1470449346.00	865137131.00	1714296767.00	0.00	0.00	0.00
Gamma-bisabolene, (*E*)-	53585-13-0	Terpenoids	29.85	0.00	218553983.00	27681532.00	0.00	0.00	0.00	0.00	0.00	0.00	0.00	109020646.00	1687923396.00	0.00	0.00	0.00	0.00	0.00	0.00
3-Pentanol	584-02-1	Alcohols	8.92	0.00	0.00	0.00	0.00	0.00	0.00	0.00	0.00	0.00	653003237.00	198658152.00	587867927.00	0.00	0.00	0.00	0.00	0.00	0.00
2,3-Dimethyl pyrazine	5910-89-4	Others	16.07	0.00	0.00	0.00	0.00	0.00	0.00	14071408.00	22265161.00	20101268.00	0.00	0.00	0.00	0.00	0.00	0.00	80848308.00	109499386.00	148390018.00
2,3,5-Trimethylpyrazine	14667-55-1	Others	17.73	0.00	0.00	0.00	0.00	0.00	0.00	30341651.00	24731457.00	16623071.00	0.00	0.00	0.00	0.00	0.00	0.00	0.00	0.00	0.00
Trans-linalool oxide	34995-77-2	Others	19.54	0.00	0.00	0.00	0.00	0.00	0.00	0.00	0.00	0.00	0.00	0.00	0.00	0.00	0.00	0.00	561266090.00	475452788.00	0.00

Beta-cadinene	523-47-7	Terpenoids	26.28	0.00	0.00	0.00	0.00	0.00	0.00	0.00	0.00	0.00	2688193450.00	0.00	801741905.00	0.00	0.00	0.00	0.00	0.00	0.00
Acetic acid, 2-phenylethyl ester	103-45-7	Carboxylic acids	27.53	0.00	0.00	0.00	0.00	0.00	0.00	150230700.00	242012187.00	304981721.00	0.00	350652982.00	1269763936.00	0.00	0.00	0.00	0.00	0.00	0.00
5-Azulenemethanol, 1,2,3,3a,4,5,6,7-octahydro-alpha,alpha,3,8-tetramethyl-, (3s,3as,5r)-	22451-73-6	Terpenoids	35.38	742741617.00	525541884.00	3868115776.00	2889789054.00	679304962.00	991289377.00	0.00	0.00	0.00	0.00	0.00	0.00	1081117775.00	1703168376.00	0.00	0.00	0.00	0.00
Alpha-eudesmol	473-16-5	Terpenoids	35.57	0.00	0.00	0.00	140635033.00	101498817.00	251019081.00	0.00	0.00	0.00	0.00	0.00	0.00	24997617193.00	15862391630.00	2754212913.00	0.00	0.00	0.00
Isovanillin	621-59-0	Aldehyde	41.23	14453516.00	0.00	28205856.00	0.00	0.00	0.00	0.00	11546298.00	5662209.00	42893173.00	11634731.00	0.00	32864150.00	29369743.00	13847468.00	10301340.00	0.00	12313227.00
Methyl isobutyrate	547-63-7	Carboxylic acids	3.67	120456.00	574833.00	432867.00	6131096.00	14916962.00	30067691.00	4172665.00	907897.00	4164789.00	11723095.00	0.00	370261.00	0.00	0.00	0.00	6839345.00	24418298.00	4051065.00
2-Pentanone	107-87-9	Ketones	4.74	0.00	0.00	0.00	0.00	0.00	0.00	0.00	0.00	0.00	0.00	0.00	0.00	250553241.00	298851129.00	0.00	8020226152.00	2804648850.00	0.00
2-Pentanol	6032-29-7	Alcohols	9.36	0.00	0.00	0.00	0.00	0.00	0.00	0.00	6444927.00	365410918.00	388616231.00	0.00	1174958961.00	382302069.00	186651086.00	0.00	0.00	0.00	0.00
3,4-Dimethoxyphenol	2033-89-8	Hydrocarbons	25.80	0.00	0.00	0.00	0.00	0.00	0.00	2145617725.00	0.00	1228676833.00	0.00	0.00	0.00	0.00	0.00	0.00	0.00	0.00	0.00
Noralfentanil	61086-18-	Hydrocarbons	14.44	0.00	0.00	0.00	0.00	0.00	0.00	11016931.00	4398269.00	0.00	0.00	0.00	0.00	0.00	0.00	0.00	0.00	0.00	0.00

	8																				
Neral	106-26-3	Terpenoids	24.56	0.00	0.00	0.00	0.00	0.00	0.00	4754392084.00	9026550494.00	0.00	0.00	0.00	0.00	63307717880.00	229180000000.00	44071321041.00	0.00	0.00	0.00
1-Heptanol	111-70-6	Alcohols	19.21	1082274890.00	1300676731.00	0.00	0.00	0.00	0.00	0.00	0.00	0.00	0.00	0.00	0.00	0.00	0.00	0.00	0.00	1407653392.00	1396347417.00
Methyl dec-4-enoate	1191-02-2	Carboxylic acids	23.33	3738181902.00	1212523072.00	787795652.00	29780092.00	97431333.00	53104797.00	0.00	450254303.00	462153102.00	0.00	0.00	0.00	0.00	0.00	0.00	0.00	0.00	0.00
(-)-Borneol	464-45-9	Terpenoids	25.13	0.00	0.00	0.00	0.00	0.00	0.00	0.00	0.00	0.00	5624939286.00	0.00	1757056564.00	0.00	0.00	0.00	5142071513.00	0.00	5801314098.00
4-Hydroxychalcone	2657-25-2	Ketones	25.52	100194613.00	0.00	93954439.00	0.00	0.00	0.00	0.00	0.00	0.00	0.00	0.00	0.00	0.00	0.00	0.00	0.00	0.00	0.00
Pyranoid linalool oxide, trans	39028-58-5	Alcohols	26.48	0.00	0.00	0.00	0.00	0.00	0.00	2034194562.00	1281360248.00	817081585.00	7597984291.00	5638538278.00	5485065172.00	0.00	0.00	0.00	1516646105.00	520327730.00	440493599.00
Isobutyl alcohol	78-83-1	Alcohols	8.37	0.00	0.00	0.00	0.00	0.00	0.00	57730661.00	83796515.00	156440488.00	2118737960.00	318006016.00	293617500.00	0.00	0.00	0.00	0.00	50977615.00	55850637.00
3-Penten-2-one	625-33-2	Ketones	9.18	0.00	0.00	0.00	0.00	197265655.00	303302970.00	0.00	0.00	0.00	0.00	0.00	0.00	0.00	0.00	0.00	0.00	0.00	0.00
Indan	496-11-7	Hydrocarbons	16.52	0.00	0.00	0.00	0.00	0.00	0.00	0.00	0.00	0.00	0.00	0.00	0.00	1723661.00	0.00	4505752.00	0.00	0.00	0.00
4-Decenol, (4z)-	57074-37-0	Alcohols	27.24	0.00	852101451.00	576671731.00	0.00	0.00	0.00	0.00	0.00	0.00	0.00	0.00	0.00	0.00	0.00	0.00	0.00	0.00	0.00
Machilol	1209-71-8	Terpenoids	34.62	353600859.00	80888927.00	412192755.00	0.00	0.00	0.00	0.00	0.00	0.00	0.00	0.00	0.00	7688777530.00	7228042993.00	952079656.00	0.00	0.00	0.00
2-Methylpent	565-	Ketones	5.23	0.00	0.00	0.00	0.00	0.00	0.00	0.00	0.00	0.00	0.00	0.00	0.00	0.00	0.00	0.00	25044032	0.00	0.00

an-3-one	69-5																		.00		
2-Methylheptan-2-ol	625-25-2	Alcohols	5.16	0.00	0.00	0.00	0.00	0.00	0.00	0.00	0.00	0.00	0.00	0.00	0.00	190507268.00	1737642547.00	0.00	0.00	0.00	0.00
3-Octanone	106-68-3	Ketones	13.31	0.00	0.00	0.00	0.00	0.00	0.00	0.00	0.00	0.00	128375727.00	90847887.00	0.00	0.00	0.00	0.00	0.00	0.00	0.00
Methyl 3-hydroxy-3-methylbutanoate	6149-45-7	Carboxylic acids	16.85	0.00	0.00	0.00	0.00	0.00	0.00	26453155.00	0.00	2948894.00	0.00	0.00	0.00	0.00	0.00	0.00	0.00	0.00	0.00
Hexyl hexanoate	6378-65-0	Carboxylic acids	23.01	102381288.00	4336695900.00	7748441015.00	0.00	0.00	0.00	0.00	0.00	0.00	0.00	0.00	0.00	0.00	0.00	0.00	0.00	0.00	0.00
Alpha,alpha-dimethyl-4-methylenecyclohexanemethanol	7299-42-5	Terpenoids	24.45	0.00	0.00	0.00	4055938779.00	612159731.00	446871641.00	542939184.00	407707494.00	346453821.00	0.00	0.00	0.00	0.00	3391776135.00	5411805132.00	1429513491.00	1345564114.00	0.00
Trans-Geranic acid methyl ester	1189-09-9	Terpenoids	24.89	394677644.00	18130852.00	256377544.00	0.00	0.00	0.00	0.00	0.00	0.00	0.00	0.00	0.00	0.00	0.00	0.00	0.00	0.00	0.00
Dodecanal	112-54-9	Aldehyde	25.28	3163562636.00	54643145787.00	25914376926.00	0.00	0.00	0.00	0.00	0.00	0.00	0.00	0.00	0.00	0.00	0.00	0.00	0.00	0.00	0.00
alpha-Muurolene	31983-22-9	Terpenoids	25.58	2426307265.00	0.00	1518258887.00	29518300995.00	16044932027.00	15006746437.00	0.00	0.00	0.00	3046936721.00	0.00	313899192.00	54736241394.00	57544970613.00	32782017854.00	754260545.00	790529479.00	0.00
(3*E*,5*E*)-2,6-Dimethylocta-3,5,7-trien-2-ol	206115-88-0	Alcohols	27.75	0.00	0.00	0.00	1945393290.00	195211754.00	297025821.00	0.00	0.00	0.00	0.00	0.00	0.00	6684030059.00	2331198413.00	1417494618.00	0.00	0.00	0.00

Rosifoliol	63891-61-2	Alcohols	33.77	823515436.00	1776732302.00	0.00	8980398708.00	9554084.00	12686560.00	0.00	0.00	0.00	0.00	0.00	0.00	1673433905.00	0.00	300914190.00	0.00	0.00	0.00
Methyl linolelaidate	2566-97-4	Carboxylic acids	34.17	304340848.00	0.00	348108835.00	0.00	0.00	0.00	0.00	0.00	0.00	0.00	0.00	0.00	0.00	0.00	0.00	0.00	0.00	0.00
Agarospirol	1460-73-7	Terpenoids	34.79	0.00	0.00	0.00	0.00	0.00	0.00	0.00	0.00	0.00	0.00	0.00	0.00	0.00	4674090234.00	704000049.00	0.00	0.00	0.00
Valproic acid	99-66-1	Carboxylic acids	11.82	0.00	0.00	0.00	0.00	0.00	0.00	0.00	0.00	0.00	0.00	0.00	0.00	0.00	0.00	0.00	815853524.00	0.00	1229766346.00
N-Hexyl acetate	142-92-7	Carboxylic acids	13.98	211730.00	106772284.00	3551757.00	0.00	0.00	0.00	91124998.00	71326582.00	140531767.00	292157252.00	278575276.00	0.00	0.00	0.00	0.00	22921258.00	36263965.00	84357442.00
2-Octen-1-ol, (2e)-	18409-17-1	Alcohols	23.10	0.00	0.00	0.00	0.00	0.00	0.00	0.00	0.00	0.00	0.00	0.00	0.00	0.00	0.00	121467699.00	0.00	0.00	0.00
Cis-4,10-Epoxyamorphane	178737-43-4	Terpenoids	24.96	1240909495.00	269237735.00	1608054613.00	12849161875.00	6915429561.00	0.00	0.00	0.00	0.00	0.00	0.00	0.00	21842850769.00	19871322453.00	16539671395.00	994015141.00	924133336.00	1034544534.00
3-Decen-1-ol, (3*Z*)-	10340-22-4	Carboxylic acids	27.01	0.00	651323649.00	738628218.00	0.00	0.00	0.00	0.00	0.00	0.00	0.00	0.00	0.00	0.00	0.00	0.00	0.00	0.00	0.00
4-Amino-3-phenylbutyric acid	1078-21-3	Carboxylic acids	27.28	0.00	183568222.00	1090659362.00	0.00	0.00	0.00	0.00	0.00	0.00	0.00	0.00	0.00	0.00	0.00	0.00	0.00	0.00	0.00
2,4-Decadienal	2363-88-4	Aldehyde	27.44	0.00	1174834138.00	1386358446.00	0.00	0.00	0.00	0.00	0.00	0.00	0.00	0.00	0.00	0.00	0.00	0.00	0.00	0.00	0.00
2-Decenol,	1840	Alcohols	27.69	0.00	69107267	37603587	0.00	0.00	0.00	0.00	0.00	0.00	0.00	0.00	0.00	0.00	0.00	0.00	0.00	0.00	0.00

(2*E*)-	9-18-2				78.00	50.00															
11-Tetradecenyl acetate, (11*Z*)-	20711-10-8	Others	29.53	0.00	118471848.00	71288247.00	0.00	0.00	0.00	0.00	0.00	0.00	0.00	0.00	0.00	0.00	0.00	0.00	0.00	0.00	0.00
1-Dodecanol	112-53-8	Alcohols	30.81	0.00	1793035492.00	1088354727.00	0.00	0.00	0.00	0.00	0.00	0.00	0.00	0.00	0.00	0.00	0.00	0.00	0.00	0.00	0.00
2-Undecen-1-ol, (2*E*)-	75039-84-8	Alcohols	31.90	46655310.00	246398834.00	0.00	0.00	0.00	0.00	0.00	0.00	0.00	0.00	0.00	0.00	0.00	0.00	0.00	0.00	0.00	0.00
Methyl 9,15-linoleate, (9*Z*,15*Z*)-	17309-05-6	Carboxylic acids	41.63	19732448.00	60849612.00	0.00	0.00	0.00	0.00	0.00	0.00	0.00	0.00	0.00	0.00	0.00	0.00	0.00	0.00	0.00	0.00
Methyl (e)oct-3-enoate	35234-16-3	Carboxylic acids	19.08	0.00	2542867.00	408921753.00	0.00	0.00	0.00	0.00	0.00	0.00	0.00	0.00	0.00	0.00	0.00	0.00	0.00	0.00	0.00
Decanal dimethyl acetal	7779-41-1	Aldehyde	22.04	0.00	845174628.00	3780474901.00	0.00	0.00	0.00	0.00	0.00	0.00	0.00	0.00	0.00	0.00	0.00	0.00	0.00	0.00	0.00
Octyl butyrate	110-39-4	Carboxylic acids	23.20	0.00	1064503963.00	758585639.00	0.00	0.00	0.00	0.00	0.00	0.00	0.00	0.00	0.00	0.00	0.00	0.00	0.00	0.00	0.00
4,7-Dimethyl-1-(propan-2-yl)-1,2,3,4,5,6-hexahydronaphthalene	16729-00-3	Carboxylic acids	24.09	196625595.00	55279322.00	112485173.00	0.00	0.00	0.00	0.00	0.00	0.00	0.00	0.00	0.00	179861183.00	677147224.00	0.00	0.00	0.00	0.00

3,5-Di-tert-butyl-4-hydroxypropiophenone	14035-34-8	Ketones	29.84	220024058.00	0.00	112912771.00	0.00	0.00	0.00	28154939.00	502209.00	42162881.00	0.00	0.00	0.00	0.00	1862465656.00	27199499.00	0.00	0.00	0.00
Tetraethylene glycol	112-60-7	Alcohols	43.74	0.00	0.00	0.00	0.00	0.00	0.00	0.00	0.00	0.00	53661599.00	0.00	174734292.00	184925345.00	0.00	415312434.00	0.00	0.00	0.00
Malonamic acid	2345-56-4	Carboxylic acids	3.32	0.00	0.00	0.00	0.00	0.00	0.00	14871110876.00	16595418449.00	0.00	0.00	0.00	0.00	0.00	0.00	0.00	0.00	0.00	0.00
Oxypinocamphone	10136-65-9	Ketones	30.53	0.00	0.00	0.00	0.00	0.00	0.00	0.00	0.00	0.00	0.00	0.00	0.00	0.00	0.00	0.00	269264605.00	361797968.00	330240613.00
(5S,6R,7S,10R)-7-Isopropyl-2,10-dimethylspiro[4.5]dec-1-en-6-ol	72203-99-7	Terpenoids	32.08	0.00	0.00	0.00	301573349.00	216664096.00	177245840.00	0.00	0.00	0.00	0.00	0.00	0.00	21421248.00	30588874.00	0.00	5368225.00	5807852.00	11499338.00
Scoparone	120-08-1	Phenols	8.93	0.00	0.00	0.00	0.00	0.00	0.00	0.00	0.00	0.00	0.00	0.00	0.00	0.00	9040131823.00	30242816482.00	0.00	0.00	0.00
Methyl heptyl ketone	821-55-6	Ketones	17.29	0.00	0.00	0.00	0.00	0.00	0.00	6887935449.00	8291548764.00	18457083710.00	0.00	0.00	0.00	0.00	0.00	0.00	0.00	0.00	0.00
(*E*)-15,16-Dinorlabda-8(17),11-dien-13-one	76497-69-3	Ketones	41.76	0.00	0.00	0.00	0.00	0.00	0.00	0.00	0.00	0.00	0.00	0.00	0.00	152973744.00	156286627.00	0.00	0.00	0.00	0.00
Bornyl formate	7492-41-3	Carboxylic acids	22.05	0.00	0.00	0.00	0.00	0.00	0.00	0.00	0.00	0.00	0.00	0.00	0.00	0.00	0.00	0.00	0.00	53751406.00	227093897.00

Methacrolein	78-85-3	Aldehyde	3.07	0.00	0.00	0.00	29047367.00	0.00	8906003.00	0.00	0.00	0.00	37252339.00	0.00	28429579.00	0.00	2782617.00	1162974.00	103255216.00	46565141.00	25483819.00
3-Octen-2-one	1669-44-9	Ketones	17.76	10566004.00	233073.00	0.00	10897450.00	16112235.00	16487598.00	0.00	0.00	0.00	0.00	0.00	0.00	197321662.00	0.00	776544026.00	0.00	4681776501.00	5008751773.00
Methyl 2-octenoate, (e)-	7367-81-9	Carboxylic acids	20.23	0.00	0.00	0.00	0.00	0.00	0.00	0.00	0.00	0.00	0.00	0.00	0.00	4997942684.00	0.00	766868946.00	0.00	0.00	0.00
(+)-T-cadinol	133645-25-7	Terpenoids	35.17	0.00	0.00	0.00	1036816928.00	821734738.00	787071342.00	0.00	0.00	0.00	0.00	0.00	0.00	871424847.00	1036045653.00	651546235.00	0.00	0.00	0.00
1-Undecene	821-95-4	Hydrocarbons	9.94	0.00	0.00	0.00	0.00	0.00	0.00	0.00	0.00	0.00	0.00	0.00	0.00	464355517.00	76466738.00	0.00	299184496.00	377505089.00	466165217.00
5-Methyl-2-furylmethylketone	1193-79-9	Ketones	22.49	0.00	0.00	0.00	2734512103.00	1934226069.00	0.00	0.00	0.00	0.00	0.00	63541.00	17094679.00	1517137697.00	0.00	346969303.00	0.00	0.00	0.00
Methylenecyclobutane	1120-56-5	Hydrocarbons	1.86	0.00	0.00	0.00	11918356082.00	0.00	73297989.00	0.00	0.00	0.00	0.00	0.00	0.00	0.00	0.00	0.00	0.00	0.00	0.00
Propionaldehyde	123-38-6	Aldehyde	2.33	0.00	0.00	0.00	196619482.00	65804138.00	39499307.00	0.00	0.00	0.00	261531155.00	211169285.00	262660549.00	673606341.00	101674294.00	0.00	700088523.00	449588264.00	570412130.00
Butane, 1-methoxy-3-methyl-	626-91-5	Alcohols	2.41	0.00	0.00	0.00	39385277.00	0.00	2256433.00	81253883.00	31245560.00	73523810.00	0.00	0.00	0.00	0.00	0.00	0.00	0.00	0.00	0.00
Beta-ocimene epoxide, (*E*)-	28977-57-3	Terpenoids	19.89	0.00	0.00	0.00	1942416052.00	35338019.00	47847473.00	0.00	0.00	0.00	0.00	0.00	0.00	0.00	0.00	0.00	0.00	0.00	0.00
Alpha-gurjun	489-	Terpenoi	20.94	0.00	0.00	0.00	35708189	19706476	19567882	0.00	0.00	0.00	10366171	16431249	0.00	0.00	0.00	0.00	0.00	0.00	0.00

ene	40-7	ds					99.00	68.00	20.00				5.00	6.00							
5-O-Desmethylomeprazole sulfide	103877-02-7	Others	22.78	0.00	0.00	0.00	3446138677.00	0.00	19609146358.00	0.00	1415770939.00	513346574.00	0.00	0.00	0.00	7317755289.00	813257637.00	0.00	0.00	0.00	0.00
4-Terpineol, (-)-	20126-76-5	Terpenoids	22.81	0.00	0.00	0.00	32724166616.00	26121209216.00	0.00	0.00	0.00	0.00	0.00	0.00	0.00	0.00	0.00	0.00	0.00	0.00	0.00
Salvial-4(14)-en-1-one	73809-82-2	Terpenoids	31.56	0.00	0.00	0.00	547410740.00	105529419.00	72356397.00	0.00	0.00	0.00	0.00	0.00	0.00	0.00	1594272119.00	546940818.00	0.00	0.00	0.00
Benzoic acid, 2-(acetylamino)-, methyl ester	2719/8/6	Carboxylic acids	39.92	0.00	0.00	0.00	136557663.00	107796296.00	79366505.00	0.00	0.00	0.00	21657547.00	12043805.00	0.00	0.00	0.00	0.00	0.00	0.00	0.00
Oplopanone	1911-78-0	Others	40.80	0.00	0.00	0.00	115967273.00	84028389.00	65158631.00	0.00	0.00	0.00	0.00	0.00	0.00	0.00	0.00	0.00	0.00	0.00	0.00
Ethylene glycol	107-21-1	Alcohols	8.37	0.00	0.00	0.00	37803954.00	77294880.00	0.00	0.00	0.00	0.00	0.00	0.00	0.00	0.00	0.00	0.00	0.00	0.00	0.00
2-Butylfuran	4466-24-4	Others	9.50	874553.00	13381726.00	13922852.00	14732717.00	64703261.00	174994329.00	0.00	0.00	0.00	0.00	0.00	0.00	42168124.00	619653770.00	61108526.00	347034302.00	113190707.00	32457876.00
1-Penten-3-ol	616-25-1	Alcohols	10.57	0.00	0.00	0.00	0.00	0.00	0.00	0.00	0.00	0.00	3846745757.00	5022372783.00	5486503785.00	0.00	0.00	0.00	9504862469.00	2474725060.00	6279609581.00
2,2,6-Trimethylcyclohexanone	2408-37-9	Carboxylic acids	15.12	0.00	0.00	0.00	59917683.00	67941215.00	54274989.00	0.00	0.00	0.00	0.00	0.00	0.00	0.00	0.00	0.00	43881698.00	49667367.00	46441041.00
Ethametsulfur	9778	Carboxyli	20.09	0.00	0.00	0.00	0.00	52483954	60361200	0.00	0.00	0.00	0.00	0.00	0.00	0.00	0.00	0.00	0.00	0.00	0.00

on-methyl [iso]	0-06-8	c acids						68.00	43.00												
Propanoic acid, 2-methyl-	156564-41-9	Carboxylic acids	22.25	0.00	0.00	0.00	0.00	125655362.00	177554433.00	0.00	0.00	0.00	0.00	0.00	0.00	0.00	0.00	0.00	0.00	0.00	0.00
Isocaryophyllene	118-65-0	Terpenoids	22.05	0.00	0.00	0.00	0.00	0.00	0.00	0.00	0.00	0.00	548202104.00	375941226.00	260516027.00	0.00	0.00	0.00	0.00	0.00	0.00
3-Methyl-2-vinylmaleimide	21494-57-5	Carboxylic acids	38.75	14961659.00	10521711.00	0.00	4142683.00	22053910.00	21569746.00	0.00	0.00	0.00	49935275.00	28084717.00	36596557.00	0.00	0.00	0.00	32280453.00	41290182.00	53988732.00
Camphor	76-22-2	Terpenoids	20.54	0.00	0.00	0.00	907541090.00	483406087.00	907911312.00	0.00	0.00	0.00	0.00	0.00	0.00	0.00	0.00	0.00	0.00	0.00	0.00
3-Hexenoic acid	1577-18-0	Carboxylic acids	30.78	0.00	0.00	0.00	0.00	1961863999.00	753880219.00	0.00	0.00	0.00	6147170264.00	0.00	10113163870.00	0.00	0.00	0.00	0.00	0.00	0.00
Methyl 4-decenoate, (4*Z*)-	7367-83-1	Carboxylic acids	23.97	0.00	37765947.00	26436336.00	0.00	0.00	0.00	0.00	0.00	0.00	0.00	0.00	0.00	0.00	0.00	0.00	0.00	0.00	0.00
Geranial	141-27-5	Terpenoids	25.73	0.00	0.00	0.00	0.00	0.00	0.00	0.00	0.00	0.00	0.00	0.00	0.00	115593500141.00	90551043078.00	0.00	0.00	0.00	0.00
2-Trans-4-trans-decadienal	25152-84-5	Aldehyde	27.44	0.00	0.00	0.00	0.00	0.00	0.00	0.00	0.00	0.00	0.00	0.00	0.00	7858896931.00	4941368725.00	2789917532.00	0.00	0.00	0.00
4-Ethyltoluene	622-96-8	Hydrocarbons	13.40	0.00	0.00	0.00	0.00	0.00	0.00	0.00	0.00	0.00	0.00	0.00	0.00	0.00	0.00	0.00	10533351.00	0.00	2317548.00
Citral	5392-40-5	Terpenoids	25.73	0.00	0.00	0.00	0.00	0.00	0.00	0.00	0.00	0.00	0.00	0.00	0.00	0.00	315602000000.00	71544207502.00	0.00	0.00	0.00

2-Undecenal	2463-77-6	Hydrocarbons	26.18	0.00	0.00	0.00	0.00	0.00	0.00	0.00	0.00	0.00	0.00	0.00	0.00	1679965796.00	238867056.00	0.00	0.00	0.00	0.00
3,5-Octadien-2-one, (3*E*,5*E*)-	30086-02-3	Ketones	20.69	0.00	0.00	0.00	0.00	0.00	0.00	0.00	0.00	0.00	0.00	0.00	0.00	564546773.00	488040936.00	0.00	11561347555.00	9267412754.00	12711916233.00
2-Octylfuran	4179-38-8	Others	21.10	0.00	0.00	0.00	0.00	0.00	0.00	0.00	0.00	0.00	0.00	0.00	0.00	1815618810.00	1637739130.00	1311732601.00	0.00	0.00	0.00
Methyl geranate	2349-14-6	Carboxylic acids	24.90	0.00	0.00	0.00	0.00	0.00	0.00	0.00	0.00	0.00	0.00	0.00	0.00	13097061773.00	33023763998.00	21532381471.00	0.00	0.00	0.00
Nerol acetate	141-12-8	Carboxylic acids	26.29	0.00	0.00	0.00	0.00	0.00	0.00	5991227120.00	8875655050.00	6207485339.00	0.00	0.00	0.00	0.00	0.00	0.00	0.00	0.00	0.00
Methyl nicotinate [usan]	93-60-7	Carboxylic acids	26.71	0.00	0.00	0.00	0.00	0.00	0.00	2633274.00	25930115.00	84865965.00	680988653.00	6312896.00	0.00	0.00	0.00	0.00	0.00	0.00	0.00
Delta-hexalactone	823-22-3	Carboxylic acids	26.98	0.00	0.00	0.00	0.00	0.00	0.00	0.00	0.00	0.00	0.00	0.00	0.00	0.00	0.00	0.00	51135128.00	0.00	22940539.00
Dimethyl azelate	1732-10-1	Carboxylic acids	34.00	14058881.00	12111769.00	7304578.00	152103558.00	67745559.00	57273266.00	0.00	0.00	0.00	99989011.00	30145795.00	51793731.00	0.00	0.00	0.00	0.00	0.00	0.00
Methyl 3,4-dimethoxybenzoate	2150-38-1	Carboxylic acids	39.37	0.00	0.00	0.00	0.00	0.00	0.00	0.00	0.00	0.00	0.00	0.00	0.00	53027394.00	64103012.00	44917171.00	0.00	0.00	0.00
11-Octadecenoic acid, methyl ester	52380-33-3	Carboxylic acids	39.55	0.00	0.00	0.00	0.00	0.00	0.00	0.00	0.00	0.00	0.00	0.00	0.00	304362061.00	180650873.00	196532419.00	0.00	0.00	0.00
Guanidinopropionic acid	353-09-3	Carboxylic acids	14.75	37089.00		0.00	0.00	0.00	0.00	0.00	0.00	0.00	0.00	0.00	0.00	0.00	0.00	0.00	0.00	0.00	0.00

Pentanoic acid, 2-pentyl ester	89155-38-4	Carboxylic acids	15.04	0.00	0.00	0.00	0.00	0.00	0.00	0.00	0.00	0.00	0.00	0.00	0.00	143406410.00	2410167808.00	0.00	0.00	0.00	0.00
4,7,7-Trimethylbicyclo[4.1.0]hept-3-en-2-one	81800-50-2	Terpenoids	32.47	0.00	0.00	0.00	0.00	0.00	0.00	52366902.00	76728659.00	0.00	0.00	0.00	0.00	0.00	0.00	0.00	0.00	0.00	0.00
Thymol	89-83-8	Terpenoids	34.85	0.00	0.00	0.00	0.00	0.00	0.00	0.00	0.00	0.00	0.00	37460957.00	27259426.00	0.00	0.00	0.00	0.00	0.00	0.00
2-Octen-1-ol, (2*Z*)-	26001-58-1	Alcohols	23.09	419385652.00	0.00	998181588.00	0.00	0.00	0.00	0.00	0.00	0.00	0.00	0.00	0.00	0.00	0.00	0.00	0.00	2008837552.00	2259700549.00
4-Thujanol	17699-16-0	Terpenoids	22.67	0.00	0.00	0.00	0.00	0.00	0.00	0.00	0.00	0.00	0.00	85055589.00	7702494.00	0.00	0.00	0.00	0.00	0.00	0.00
Norquetiapine	5747-48-8	Others	24.73	0.00	0.00	0.00	3305890871.00	0.00	0.00	0.00	0.00	0.00	0.00	0.00	0.00	0.00	0.00	0.00	0.00	0.00	0.00
3-Nonen-1-ol, (3*Z*)-	10340-23-5	Alcohols	16.43	0.00	0.00	0.00	0.00	0.00	227755756.00	0.00	0.00	0.00	0.00	0.00	0.00	0.00	0.00	0.00	0.00	0.00	0.00
2-Octanone	111-13-7	Ketones	14.31	0.00	0.00	0.00	0.00	0.00	0.00	0.00	0.00	0.00	0.00	0.00	0.00	61069259.00	20094144.00	219829553.00	1050859126.00	1238669188.00	1355143606.00
Methyl phenethyl ether	3558-60-9	Ethers	19.86	0.00	0.00	0.00	0.00	0.00	0.00	108760601.00	75945793.00	0.00	0.00	0.00	0.00	0.00	0.00	0.00	0.00	0.00	0.00
Methyl	74-9	Others	1.92	291391.0	2619435.	0.00	50263.00	1788830.	502873.0	0.00	0.00	0.00	23595400	24566072	979649.0	0.00	0.00	0.00	26752076	1330491.	9864548.

mercaptan	3-1			0	00			00	0				16.00	114.00	0				.00	00	00
Isoamyl acetate	123-92-2	Alcohols	9.17	0.00	0.00	0.00	0.00	0.00	0.00	696871201.00	1079658078.00	826708113.00	0.00	0.00	0.00	0.00	0.00	0.00	0.00	0.00	0.00
N-Amyl acetate	628-63-7	Alcohols	10.89	0.00	0.00	0.00	0.00	0.00	0.00	0.00	0.00	0.00	0.00	0.00	0.00	0.00	0.00	0.00	113221523.00	177438691.00	439336043.00
N,N-Dimethyl formamide	1968-12-02 00:00:00	Carboxylic acids	17.44	0.00	0.00	0.00	0.00	0.00	0.00	0.00	0.00	0.00	1628799291.00	1322894602.00	2022445533.00	0.00	0.00	0.00	0.00	0.00	0.00
ethyl-2-methyl butyrate	7452-79-1	Carboxylic acids	6.96	0.00	0.00	0.00	0.00	0.00	0.00	46588317.00	446391811.00	259710831.00	0.00	0.00	0.00	0.00	0.00	0.00	0.00	0.00	0.00
Hexanal, 4-methyl-	41065-97-8	Aldehyde	10.25	0.00	0.00	0.00	0.00	0.00	0.00	30085766.00	42817092.00	40280305.00	0.00	0.00	0.00	0.00	0.00	0.00	0.00	0.00	0.00
1-Hexanol, 4-methyl-, acetate	91367-59-8	Carboxylic acids	15.97	0.00	0.00	0.00	0.00	0.00	0.00	230246440.00	279621405.00	388177456.00	0.00	0.00	0.00	0.00	0.00	0.00	0.00	0.00	0.00
Hexanoic acid, 4-methylene-, methyl ester	73805-48-8	Others	16.24	0.00	0.00	0.00	0.00	0.00	0.00	39036646.00	93525732.00	43645220.00	0.00	0.00	0.00	0.00	0.00	0.00	0.00	0.00	0.00
Alpha-farnesene, (3*Z*,6*E*)-	26560-14-5	Terpenoids	25.59	0.00	0.00	0.00	0.00	0.00	0.00	1974056679.00	1842875737.00	0.00	0.00	0.00	0.00	0.00	0.00	0.00	0.00	0.00	0.00
2,6-Dimethyl	1374	Alcohols	30.37	0.00	0.00	0.00	0.00	0.00	0.00	13502719	10726466	54302861	88153113	30592896	28382642	0.00	0.00	0.00	0.00	0.00	0.00

octa-3,7-diene-2,6-diol	1-21-4									09.00	01.00	7.00	.00	4.00	3.00						
Dimethyl dl-malate	38115-87-6	Carboxylic acids	31.31	0.00	0.00	0.00	0.00	0.00	0.00	55184818.00	44586166.00	0.00	0.00	0.00	0.00	0.00	0.00	0.00	0.00	0.00	0.00
1,7-Octadiene-3,6-diol, 2,6-dimethyl-	51276-33-6	Alcohols	33.88	0.00	0.00	0.00	0.00	0.00	0.00	576258932.00	466149361.00	463920220.00	38339681.00	234249382.00	1654441190.00	0.00	0.00	0.00	0.00	0.00	0.00
2,7-Octadiene-1,6-diol, 2,6-dimethyl-	64142-78-5	Alcohols	37.17	0.00	0.00	0.00	0.00	0.00	0.00	153587954.00	0.00	151629258.00	0.00	0.00	0.00	0.00	0.00	0.00	0.00	0.00	0.00
Isoprene	78-79-5	Terpenoids	1.86	0.00	0.00	0.00	0.00	0.00	0.00	398376067.00	1389998737.00	0.00	0.00	0.00	0.00	0.00	0.00	0.00	0.00	0.00	0.00
Methyl isovalerate	556-24-1	Carboxylic acids	5.91	0.00	0.00	0.00	0.00	0.00	0.00	26211210797.00	4435308920.00	3102920930.00	0.00	0.00	0.00	0.00	0.00	0.00	0.00	0.00	0.00
3,7-Dimethyl octa-1,5,7-trien-3-ol	29957-43-5	Hydrocarbons	22.98	0.00	0.00	0.00	0.00	0.00	0.00	0.00	39412421.00	23461473.00	0.00	0.00	0.00	0.00	0.00	0.00	0.00	0.00	0.00
Hydroquinone dimethyl ether	150-78-7	Phenols	25.82	0.00	0.00	0.00	0.00	0.00	0.00	63193353.00	2767761159.00	50057055.00	0.00	0.00	0.00	0.00	0.00	0.00	0.00	0.00	0.00
p-Mentha-1,5-dien-8-ol	1686-20-0	Terpenoids	26.88	0.00	0.00	0.00	0.00	0.00	0.00	611107719.00	423244145.00	307389040.00	0.00	0.00	0.00	0.00	0.00	0.00	0.00	0.00	0.00
1,3,3-Trimethyl-2-oxabicyclo[2.2.2]octan-6-ol	18679-48-6	Terpenoids	28.58	0.00	0.00	0.00	0.00	0.00	0.00	0.00	639414075.00	1191323992.00	0.00	0.00	0.00	0.00	0.00	0.00	0.00	0.00	0.00

4-Acetamidobenzaldehyde	122-85-0	Aldehyde	31.77	0.00	0.00	0.00	0.00	0.00	0.00	0.00	0.00	0.00	0.00	0.00	0.00	0.00	0.00	0.00	7499155.00	0.00	1424848.00
1-Hepten-3-one	2918-13-0	Ketones	14.77	0.00	0.00	0.00	0.00	0.00	0.00	9413654.00	0.00	22563151.00	0.00	0.00	0.00	0.00	0.00	0.00	78350125.00	0.00	415202008.00
Benzylacetone	2550-26-7	Ketones	28.40	0.00	0.00	0.00	0.00	0.00	0.00	77557274.00	0.00	338396506.00	0.00	0.00	0.00	0.00	0.00	0.00	0.00	0.00	0.00
4-Phenyl-2-butanol	2344-70-9	Alcohols	31.24	0.00	0.00	0.00	0.00	0.00	0.00	0.00	40853835.00	522727321.00	0.00	0.00	0.00	0.00	0.00	0.00	0.00	0.00	0.00
Alpha,alpha-dimethoxytoluene	1125-88-8	Ethers	21.14	0.00	0.00	0.00	0.00	0.00	0.00	0.00	0.00	0.00	0.00	38709674.00	16679376.00	0.00	0.00	0.00	0.00	0.00	0.00
Dimethyl adipate	627-93-0	Carboxylic acids	27.63	0.00	0.00	0.00	0.00	0.00	0.00	410858.00	103175.00	7977154.00	20833715.00	65195774.00	1000233905.00	0.00	0.00	0.00	0.00	0.00	0.00
Nonane, 4,5-dimethyl-	17302-23-7	Hydrocarbons	10.18	0.00	0.00	0.00	0.00	0.00	0.00	0.00	0.00	0.00	26400059.00	102265079.00	540824.00	0.00	0.00	0.00	0.00	0.00	0.00
Brevicomin	20290-99-7	Others	14.72	0.00	0.00	0.00	0.00	0.00	0.00	821785.00	14657514.00	26940663.00	0.00	0.00	0.00	0.00	0.00	0.00	0.00	0.00	0.00
2-Methylnaphthalene	91-57-6	Hydrocarbons	28.94	19889913.00	3594738.00	0.00	0.00	9254753.00	16512085.00	0.00	0.00	0.00	0.00	0.00	0.00	0.00	0.00	0.00	15862.00	0.00	1518634.00
8-Chlorotheophylline	85-18-7	Others	13.67	17260601.00	16341109.00	0.00	0.00	0.00	0.00	0.00	0.00	0.00	0.00	0.00	0.00	0.00	0.00	0.00	0.00	0.00	0.00
2-Amino-4-methylphenol	95-84-1	Phenols	13.42	0.00	0.00	0.00	0.00	0.00	0.00	0.00	0.00	0.00	810171.00	0.00	70058847.00	0.00	0.00	0.00	0.00	0.00	0.00
N-Pentane	109-	Hydrocar	1.70	0.00	0.00	0.00	0.00	0.00	0.00	0.00	0.00	0.00	0.00	0.00	0.00	0.00	0.00	0.00	0.00	66810612	72502497

	66-0	bons																		13.00	48.00
1,3-Pentadiene	504-60-9	Hydrocarbons	1.91	0.00	0.00	0.00	0.00	0.00	0.00	0.00	0.00	0.00	5802675715.00	3218478.00	0.00	0.00	0.00	0.00	0.00	0.00	0.00
Meglutol [usan:inn]	503-49-1	Carboxylic acids	8.83	0.00	0.00	0.00	0.00	0.00	0.00	0.00	0.00	0.00	230332197.00	27844035.00	0.00	0.00	0.00	0.00	0.00	0.00	0.00
2-Methyl-2-pentenal	623-36-9	Hydrocarbons	9.91	0.00	0.00	0.00	0.00	0.00	0.00	0.00	0.00	0.00	116315102.00	266888626.00	0.00	0.00	0.00	0.00	0.00	0.00	0.00
Methyl 2-hexenoate, (2e)-	13894-63-8	Carboxylic acids	14.43	0.00	0.00	0.00	0.00	0.00	0.00	0.00	0.00	0.00	8674209425.00	2517305395.00	2590550631.00	0.00	0.00	0.00	0.00	0.00	0.00
3-Hexen-1-ol	544-12-7	Alcohols	16.71	0.00	0.00	0.00	0.00	0.00	0.00	0.00	0.00	0.00	2738231723.00	2618089362.00	3817311897.00	0.00	0.00	0.00	0.00	0.00	0.00
3-Hexenol	928-96-1	Alcohols	17.27	0.00	0.00	0.00	0.00	0.00	0.00	0.00	0.00	0.00	77622245267.00	58326079456.00	106047374083.00	0.00	0.00	0.00	2356709237.00	2923025129.00	3258259606.00
2,4-Hexadienal	142-83-6	Hydrocarbons	17.56	0.00	0.00	0.00	0.00	0.00	0.00	0.00	0.00	0.00	3360595950.00	1902318480.00	0.00	0.00	0.00	0.00	0.00	0.00	0.00
Methyl (methylthio)acetate	16630-66-3	Carboxylic acids	17.71	0.00	0.00	0.00	0.00	0.00	0.00	345359055.00	301079200.00	0.00	121714334.00	74344206.00	0.00	0.00	0.00	0.00	0.00	0.00	0.00
Pentandioic acid, (p-t-butylphenyl) ester	212762-88-4	Carboxylic acids	17.90	0.00	0.00	0.00	0.00	0.00	0.00	0.00	0.00	0.00	2298021228.00	0.00	1310372863.00	0.00	0.00	0.00	0.00	0.00	0.00
Cycloheptane, bromo-	2404-35-5	Others	18.24	0.00	0.00	0.00	0.00	0.00	0.00	0.00	0.00	0.00	55639737.00	0.00	112350032.00	0.00	0.00	0.00	0.00	0.00	0.00
Methyl	2106	Carboxyli	18.41	0.00	0.00	0.00	0.00	0.00	0.00	0.00	0.00	0.00	26730634	78102269	21171249	0.00	0.00	0.00	66929935	15723486	0.00

4-Octenoate, (*Z*)-	3-71-8	c acids											1.00	.00	2.00				3.00	60.00	
6-Deisopropyl atrazine	1007-28-9	Others	19.20	0.00	0.00	0.00	0.00	0.00	0.00	0.00	0.00	0.00	134349528.00	72992904.00	0.00	0.00	0.00	0.00	0.00	0.00	0.00
beta-cyclocitral	432-25-7	Terpenoids	23.15	0.00	0.00	0.00	0.00	0.00	0.00	0.00	0.00	0.00	1348168868.00	0.00	557066000.00	0.00	0.00	0.00	1078093645.00	468287599.00	1874089356.00
Methyl 4-oxohexanoate	2955-62-6	Carboxylic acids	23.65	0.00	0.00	0.00	0.00	0.00	0.00	0.00	0.00	0.00	239181490.00	218698467.00	142944574.00	0.00	0.00	0.00	552524551.00	273347328.00	50072916.00
Nonanoic acid, 9-oxo-, methyl ester	1931-63-1	Aldehyde	32.43	0.00	0.00	0.00	0.00	0.00	0.00	0.00	0.00	0.00	47419341.00	0.00	19061526.00	0.00	0.00	0.00	0.00	203668624.00	24130625.00
Sinomenine	115-53-7	Hydrocarbons	34.92	0.00	0.00	0.00	0.00	0.00	0.00	0.00	0.00	0.00	21057613.00	4855293.00	0.00	0.00	0.00	0.00	0.00	0.00	0.00
Trans-phytol	150-86-7	Terpenoids	41.98	0.00	0.00	0.00	0.00	0.00	0.00	0.00	0.00	0.00	32095825.00	20371507.00	24163120.00	0.00	0.00	0.00	0.00	0.00	0.00
Tetradecyltrimethylammonium	10182-92-0	Others	1.67	0.00	0.00	0.00	0.00	0.00	0.00	0.00	0.00	0.00	0.00	870425514.00	1541259569.00	0.00	0.00	0.00	0.00	0.00	0.00
Bis(dichloromethyl) ether	20524-86-1	Ethers	5.93	0.00	0.00	0.00	0.00	0.00	0.00	0.00	0.00	0.00	5853967917.00	5279712639.00	0.00	0.00	0.00	0.00	0.00	5769993170.00	11393732731.00
3-Hexenylacetate	3681-71-8	Carboxylic acids	15.27	0.00	0.00	0.00	0.00	0.00	0.00	0.00	0.00	0.00	0.00	136040240.00	258981649.00	0.00	0.00	0.00	0.00	99679589.00	128633406.00
2-Hexen-1-ol, (2*Z*)-	928-94-9	Alcohols	18.00	0.00	0.00	0.00	0.00	0.00	0.00	0.00	0.00	0.00	12182413.00	2326657210.00	5615222342.00	0.00	0.00	0.00	0.00	0.00	0.00

3-Methylcyclopentyl acetate	24070-70-0	Carboxylic acids	10.45	0.00	0.00	0.00	0.00	0.00	0.00	0.00	0.00	0.00	3097650626.00	0.00	170506774.00	0.00	0.00	0.00	575159534.00	335299296.00	324880543.00
2H-Pyran-3(4H)-one, 6-ethenyldihydro-2,2,6-trimethyl-	33933-72-1	Others	19.43	0.00	0.00	0.00	0.00	0.00	0.00	0.00	0.00	0.00	853002099.00	751031058.00	351622889.00	0.00	0.00	0.00	0.00	0.00	0.00
2-Pentenoic acid, (2*E*)-	13991-37-2	Carboxylic acids	29.33	0.00	0.00	0.00	0.00	0.00	0.00	0.00	0.00	0.00	1064897080.00	0.00	1783695767.00	0.00	0.00	0.00	0.00	0.00	0.00
Citronellol	106-22-9	Terpenoids	26.59	0.00	0.00	0.00	0.00	0.00	0.00	0.00	0.00	0.00	0.00	0.00	0.00	0.00	6600349455.00	1346363898.00	0.00	0.00	0.00
Dihydrocarveol	38049-26-2	Alcohols	28.19	0.00	0.00	0.00	0.00	0.00	0.00	64031176.00	497920162.00	0.00	0.00	0.00	0.00	0.00	0.00	0.00	0.00	0.00	0.00
7-Methylguanine	578-76-7	Hydrocarbons	23.75	0.00	0.00	0.00	0.00	0.00	0.00	0.00	0.00	0.00	0.00	0.00	0.00	94215023473.00	0.00	1478377208.00	0.00	0.00	0.00
Sobrerol, (+)-trans-	38235-58-4	Terpenoids	37.31	0.00	0.00	0.00	0.00	0.00	0.00	0.00	0.00	0.00	0.00	0.00	0.00	354512528.00	19254058.00	640836966.00	0.00	0.00	0.00
Ylangenol	41610-69-9	Terpenoids	37.00	0.00	0.00	0.00	0.00	0.00	0.00	0.00	0.00	0.00	0.00	0.00	0.00	0.00	0.00	0.00	352875338.00	77223216.00	2537840953.00
1,2,4-Trimethoxybenzene	135-77-3	Ethers	32.93	0.00	0.00	0.00	0.00	0.00	0.00	0.00	0.00	0.00	0.00	0.00	0.00	0.00	2256880779.00	3040824286.00	0.00	0.00	0.00

5-Ethylcyclopent-1-enecarboxAldehyde	36431-60-4	Aldehyde	17.93	0.00	0.00	0.00	0.00	0.00	0.00	0.00	0.00	0.00	0.00	0.00	0.00	32235211.00	17806592.00	139439498.00	928526133.00	693547585.00	934443174.00
2-Nitrophenol	88-75-5	Phenols	21.38	0.00	0.00	0.00	0.00	0.00	0.00	0.00	0.00	0.00	0.00	0.00	0.00	250713134.00	0.00	192080463.00	0.00	0.00	0.00
1-Cyclobutylpiperidin-4-amine	1048921-27-2	Others	25.12	0.00	0.00	0.00	20565703.00	532578962.00	0.00	0.00	0.00	0.00	0.00	0.00	0.00	0.00	0.00	0.00	0.00	0.00	0.00
2-Pinene oxide	1686-14-2	Terpenoids	16.41	0.00	0.00	0.00	0.00	23014867.00	49588952.00	0.00	0.00	0.00	0.00	0.00	0.00	0.00	0.00	0.00	0.00	0.00	0.00
(1S,3aS,4S,5S,7aR,8R)-5-Isopropyl-1,7a-dimethyloctahydro-1H-1,4-methanoinden-8-ol	21966-93-8	Alcohols	34.83	0.00	0.00	0.00	0.00	0.00	0.00	0.00	0.00	0.00	0.00	0.00	0.00	0.00	0.00	0.00	971897298.00	277253027.00	260296540.00
Alpha-corocalene	20129-39-9	Terpenoids	32.51	0.00	0.00	0.00	0.00	0.00	0.00	0.00	0.00	0.00	0.00	0.00	0.00	618267058.00	0.00	397526900.00	0.00	0.00	0.00
Alpha-pvt	1400742-66-6	Others	15.68	0.00	0.00	0.00	0.00	0.00	0.00	0.00	0.00	0.00	131937307.00	545663365.00	907380354.00	0.00	0.00	0.00	0.00	0.00	0.00
Glabridin	59870-68-7	Phenols	33.24	0.00	0.00	0.00	49985704.00	74568473.00	90029435.00	0.00	0.00	0.00	0.00	0.00	0.00	0.00	0.00	0.00	0.00	0.00	0.00

1-Heptene	592-76-7	Hydrocarbons	2.11	0.00	0.00	0.00	0.00	0.00	0.00	0.00	0.00	0.00	0.00	0.00	0.00	0.00	0.00	0.00	173622021.00	12483360.00	11562347.00
2-Methylfuran	534-22-5	Others	2.96	4169973.00	32918.00	0.00	1235635.00	2556521.00	475366.00	0.00	0.00	0.00	7344263.00	7559163.00	18378832.00	800.00	23188997.00	0.00	35799433.00	23567932.00	0.00
2-Isooctanone	928-68-7	Ketones	12.87	0.00	0.00	0.00	0.00	0.00	0.00	0.00	0.00	0.00	0.00	0.00	0.00	0.00	0.00	0.00	30684279.00	0.00	12820707.00
4-Heptenal, (4*Z*)-	6728-31-0	Aldehyde	12.95	0.00	0.00	0.00	0.00	0.00	0.00	0.00	0.00	0.00	0.00	0.00	0.00	0.00	0.00	0.00	91482544.00	65726215.00	72197097.00
Cyclopentanol	96-41-3	Alcohols	14.94	0.00	0.00	0.00	0.00	0.00	0.00	0.00	0.00	0.00	0.00	0.00	0.00	0.00	0.00	0.00	167379735.00	118075579.00	0.00
Chrysanthenone	473-06-3	Terpenoids	20.33	0.00	0.00	0.00	0.00	0.00	0.00	0.00	0.00	0.00	0.00	0.00	0.00	0.00	0.00	0.00	1902445654.00	1563582143.00	856112150.00
Diamyl ketone	927-49-1	Ketones	20.94	0.00	0.00	0.00	0.00	0.00	0.00	0.00	0.00	0.00	0.00	0.00	0.00	0.00	0.00	0.00	958799543.00	900828398.00	1095348019.00
Ketoisophorone	1125-21-9	Ketones	24.80	0.00	0.00	0.00	0.00	0.00	0.00	0.00	0.00	0.00	0.00	0.00	0.00	0.00	0.00	0.00	502017878.00	0.00	693720052.00
3-Methylacetophenone	585-74-0	Ketones	26.61	0.00	0.00	0.00	0.00	0.00	0.00	0.00	0.00	0.00	0.00	0.00	0.00	0.00	0.00	0.00	219712522.00	0.00	0.00
Pyocyanine	85-66-5	Terpenoids	28.02	0.00	0.00	0.00	0.00	0.00	0.00	0.00	0.00	0.00	0.00	0.00	0.00	0.00	0.00	0.00	292270457.00	938821763.00	0.00
1H-Purine	120-73-0	Hydrocarbons	28.15	0.00	0.00	0.00	0.00	0.00	0.00	0.00	0.00	0.00	0.00	0.00	0.00	0.00	0.00	0.00	66245335.00	0.00	0.00
1,1,3,3-Tetramethylguanidine	80-70-6	Others	28.63	0.00	0.00	0.00	0.00	0.00	0.00	0.00	0.00	0.00	0.00	0.00	0.00	0.00	0.00	0.00	419707542.00	0.00	923434933.00
(S)-(+)-5-(1-	6059	Ketones	37.56	0.00	0.00	0.00	0.00	0.00	0.00	0.00	0.00	0.00	0.00	0.00	0.00	0.00	0.00	0.00	55665296	66067165	25862170

Hydroxy-1-methylethyl)-2-methyl-2-cyclohexen-1-one	3-11-5																		3.00	5.00	08.00
1,3,3-Trimethyl-6-hydroxy-2-oxabicyclo[2.2.2]octan-7-one	81968-80-1	Others	39.48	0.00	0.00	0.00	0.00	0.00	0.00	0.00	0.00	0.00	0.00	0.00	0.00	0.00	0.00	0.00	178122065.00	254900326.00	205858252.00
Furan	110-00-9	Hydrocarbons	2.36	0.00	0.00	0.00	0.00	0.00	0.00	0.00	0.00	0.00	0.00	0.00	0.00	0.00	8168152.00	31172609.00	202317170.00	114934063.00	152858987.00
Cyclopentene, 1-isopropyl-2,3-dimethyl-	7712-73-4	Hydrocarbons	16.31	0.00	0.00	0.00	0.00	0.00	0.00	0.00	0.00	0.00	0.00	0.00	0.00	0.00	0.00	0.00	0.00	639855390.00	983525169.00
Methyl 3-nonenoate	13481-87-3	Carboxylic acids	21.40	0.00	0.00	0.00	0.00	0.00	0.00	0.00	0.00	0.00	0.00	0.00	0.00	0.00	0.00	0.00	195683978.00	1409735266.00	1191475651.00
4-Tert-butyl phenol	98-54-4	Phenols	25.60	0.00	0.00	0.00	0.00	0.00	0.00	0.00	0.00	0.00	0.00	0.00	0.00	0.00	0.00	0.00	127353982.00	106883998.00	0.00
Pinanediol	22422-34-0	Alcohols	29.38	0.00	0.00	0.00	0.00	0.00	0.00	0.00	0.00	0.00	0.00	0.00	0.00	0.00	0.00	0.00	2135872430.00	4386921926.00	0.00
2-Pentanoylfuran	31940-17-0	Ketones	29.57	0.00	0.00	0.00	0.00	0.00	0.00	0.00	0.00	0.00	0.00	0.00	0.00	0.00	0.00	0.00	194683829.00	298946734.00	0.00
5,7-Dimethyl-8-hydroxyqui	37873-29-	Hydrocarbons	31.10	0.00	0.00	0.00	0.00	0.00	0.00	0.00	0.00	0.00	0.00	0.00	0.00	0.00	0.00	0.00	7784905.00	2107829625.00	0.00

noline	3																				
3-Methylfuran	930-27-8	Hydrocarbons	2.96	0.00	0.00	0.00	0.00	0.00	0.00	0.00	0.00	0.00	8897782.00	123374.00	4333058.00	0.00	0.00	0.00	2046644.00	124160.00	33991506.00
Hexane, 1,1-dimethoxy-	1599-47-9	Hydrocarbons	10.63	0.00	538965473.00	621855652.00	0.00	0.00	0.00	0.00	0.00	0.00	0.00	0.00	0.00	0.00	0.00	0.00	0.00	1048970680.00	813679713.00
3,6-Octadienal, 3,7-dimethyl-	55722-59-3	Aldehyde	21.96	0.00	0.00	0.00	0.00	0.00	0.00	0.00	0.00	0.00	0.00	0.00	0.00	167161360.00	0.00	851562855.00	0.00	0.00	0.00
Cinnamaldehyde	104-55-2	Aldehyde	32.03	8289438.00	5692164.00	0.00	0.00	0.00	0.00	0.00	0.00	0.00	0.00	0.00	0.00	0.00	0.00	0.00	0.00	0.00	0.00
Alpha-methyl cinnamAldehyde	101-39-3	Aldehyde	32.12	0.00	0.00	0.00	0.00	0.00	0.00	0.00	0.00	0.00	0.00	0.00	0.00	0.00	1083325411.00	394495747.00	0.00	0.00	0.00
Hexanoic acid, decyl ester	52363-43-6	Carboxylic acids	31.72	0.00	92174045.00	168125410.00	0.00	0.00	0.00	0.00	0.00	0.00	0.00	0.00	0.00	0.00	0.00	0.00	0.00	0.00	0.00
Ethyl octadecanoate	111-61-5	Carboxylic acids	39.69	0.00	24741477.00	27442002.00	0.00	0.00	0.00	0.00	0.00	0.00	0.00	0.00	0.00	25866708.00	23102141.00	0.00	0.00	0.00	0.00
2,5-Dimethyl pyrazine	123-32-0	Hydrocarbons	15.37	233466494.00	38174671.00	0.00	0.00	0.00	0.00	0.00	0.00	0.00	0.00	0.00	0.00	0.00	0.00	0.00	0.00	0.00	0.00
7-Methyl-Z-8,10-dodecadienal		Hydrocarbons	38.88	38105383.00	39337174.00	26801180.00	0.00	0.00	0.00	0.00	0.00	0.00	0.00	0.00	0.00	92197684.00	24489673.00	0.00	57410877.00	40548425.00	0.00
Trans-3-Hexenoic acid		Carboxylic acids	30.88	0.00	0.00	0.00	0.00	0.00	0.00	0.00	0.00	0.00	5782540609.00	728508824.00	0.00	0.00	0.00	0.00	0.00	0.00	0.00

2-Furancarboxylic acid, cyclobutyl ester	Carboxylic acids	20.24	0.00	0.00	0.00	0.00	0.00	0.00	0.00	291350994.00	195455033.00	0.00	0.00	0.00	0.00	0.00	0.00	0.00	0.00	0.00
Cis-4-methoxy thujane	Terpenoids	14.68	108477124.00	4774127.00	20715241.00	135803842.00	4021316.00	11004253.00	0.00	0.00	0.00	0.00	0.00	0.00	634037997.00	261215345.00	216771975.00	0.00	0.00	0.00
Trans-13-Octadecenoic acid, methyl ester	Carboxylic acids	39.55	96217322.00	145757032.00	0.00	0.00	0.00	0.00	0.00	0.00	0.00	0.00	0.00	0.00	0.00	0.00	0.00	0.00	0.00	0.00
Tricyclo[4.2.2.0(1,5)]dec-7-ene	Others	16.17	0.00	0.00	0.00	516060571.00	0.00	94662095.00	0.00	0.00	0.00	0.00	0.00	0.00	0.00	0.00	0.00	0.00	0.00	0.00
Cis-5-Dodecenoic acid, methyl ester	Carboxylic acids	27.74	0.00	0.00	0.00	0.00	0.00	0.00	64292162.00	49723802.00	92712113.00	0.00	0.00	0.00	0.00	0.00	0.00	0.00	0.00	0.00
Nerol, methyl ether	Ethers	18.58	0.00	0.00	0.00	0.00	0.00	0.00	0.00	0.00	0.00	0.00	0.00	0.00	1816601639.00	20548027528.00	0.00	0.00	0.00	0.00
β-Citronellol, methyl ether	Ethers	16.18	132802259.00	0.00	10929761.00	0.00	0.00	0.00	0.00	0.00	0.00	0.00	0.00	0.00	791913974.00	4345879362.00	0.00	0.00	0.00	0.00
10-Methylundec-2-en-4-olide	Ketones	38.94	0.00	0.00	0.00	0.00	0.00	0.00	0.00	0.00	0.00	0.00	0.00	0.00	88915991.00	99534261.00	43097283.00	0.00	0.00	0.00
Trans-2-Dodecenyl acetate	Carboxylic acids	30.26	0.00	0.00	0.00	0.00	0.00	0.00	0.00	0.00	0.00	0.00	0.00	0.00	0.00	0.00	0.00	0.00	1121587126.00	1398048410.00

Notes： T: *A. tsaoko*; P: *A. paratsaoko*; K: *A. koenigii*; L: Leaves; F: Fruits

Table S2. The main volatile metabolites in the leaves of three species of genus *Amomum*

Species	Main Volatile Components	Compound	Relative Content
A. tsaoko	Cineole	Terpenoids	40.3754±0.6396
	(-)-β-Pinene	Terpenoids	18.6252±0.0651
	Genraniol	Terpenoids	13.3421±1.2957
	(Z)-2-Decenal	Aldehydes	12.5867±0.6521
	(-)-Limonene	Terpenoids	12.4962±1.8275
	Linalool	Terpenoids	9.5197±0.2003
A. paratsaoko	(Z)-2-Decenal	Aldehydes	4.0542±0.4291
	(-)-β-Pinene	Terpenoids	3.8885±1.7138
	1-Octanol	Alcohols	3.6182±1.2974
	Cineole	Terpenoids	3.5136±0.5618
	Limonene	Terpenoids	1.1237±0.3481
A. koenigii	Linalool	Terpenoids	8.7264±3.9319
	Cineole	Terpenoids	6.3570±0.3483
	Borneol	Terpenoids	4.0692±1.4361
	Camphene	Terpenoids	2.7222±0.3962
	(-)-Limonene	Terpenoids	2.0261±0.1692

The relative content was expressed as the average ± standard deviation of three measurements.

Table S3. The main volatile metabolites in the fruits of three species of genus *Amomum*

Species	Main Volatile Components	Compound	Relative Content
A. tsaoko	Cineole	Terpenoids	10.5816±0.1849
	β-Caryophyllene	Terpenoids	9.1386±0.2399
	α-Pinene	Terpenoids	2.9838±0.1089
	α-Humulene	Terpenoids	2.6123±0.1062
	α-Phellandrene	Terpenoids	2.8129±0.0128
A. paratsaoko	(-)-Limonene	Terpenoids	49.9832±42.5744
	α-Ocimene	Terpenoids	14.3901±12.4622
	β-Pinene	Terpenoids	7.0435±2.0944
	Cineole	Terpenoids	6.7721±3.8136
	β-Copaene	Terpenoids	4.7296±1.7247
A. koenigii	2-Hexenal	Aldehydes	30.6481±0.0107
	Linalool	Terpenoids	15.9882±0.9929
	3-Hexenol	Alcohols	6.0411±0.2344
	α-Humulene	Terpenoids	4.0212±0.2965
	β-Caryophyllene	Terpenoids	3.179±0.2463

The relative content was expressed as the average ± standard deviation of three

A

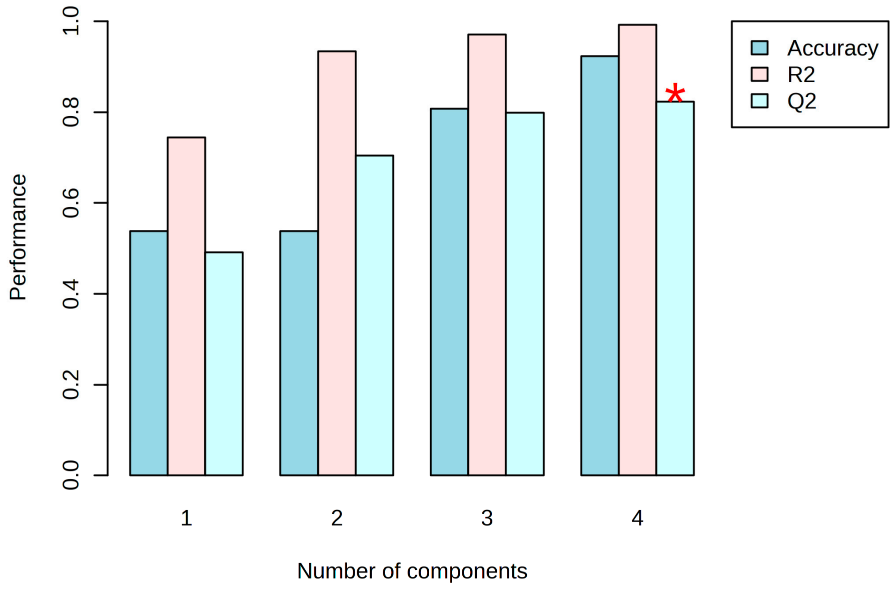

B

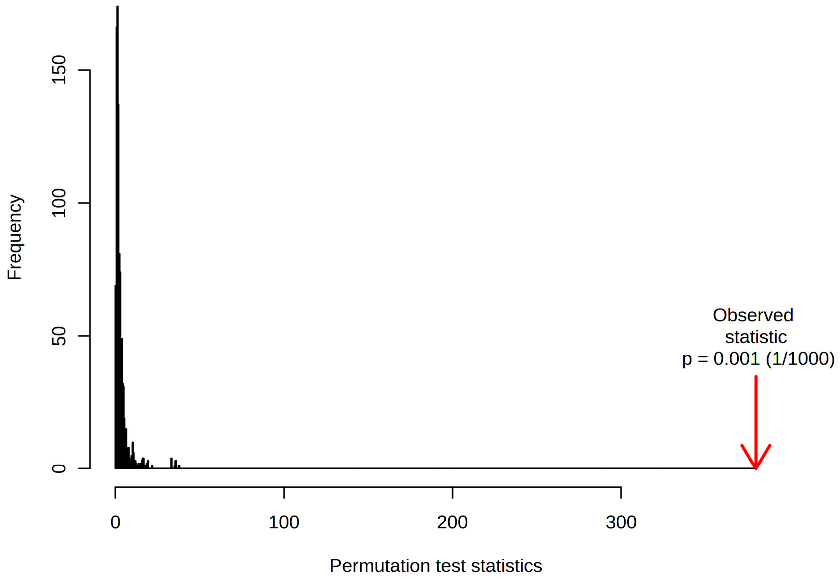

Figure S2. Cross validation and permutation tests of PLS-DA model. A. Cross validation ; B. Permutation tests.
